# Specific histone modifications associate with alternative exon selection during mammalian development

**DOI:** 10.1093/nar/gkaa248

**Published:** 2020-04-22

**Authors:** Qiwen Hu, Casey S Greene, Elizabeth A Heller

**Affiliations:** Department of Systems Pharmacology and Translational Therapeutics, University of Pennsylvania, Philadelphia, PA, USA

## Abstract

Alternative splicing (AS) is frequent during early mouse embryonic development. Specific histone post-translational modifications (hPTMs) have been shown to regulate exon splicing by either directly recruiting splice machinery or indirectly modulating transcriptional elongation. In this study, we hypothesized that hPTMs regulate expression of alternatively spliced genes for specific processes during differentiation. To address this notion, we applied an innovative machine learning approach to relate global hPTM enrichment to AS regulation during mammalian tissue development. We found that specific hPTMs, H3K36me3 and H3K4me1, play a role in skipped exon selection among all the tissues and developmental time points examined. In addition, we used iterative random forest model and found that interactions of multiple hPTMs most strongly predicted splicing when they included H3K36me3 and H3K4me1. Collectively, our data demonstrated a link between hPTMs and alternative splicing which will drive further experimental studies on the functional relevance of these modifications to alternative splicing.

## INTRODUCTION

Alternative splicing (AS) is a regulatory mechanism of gene expression that enables one gene to generate multiple mRNA isoforms that may have different functions or properties. RNA-seq analyses of the whole transcriptome have revealed the high prevalence of AS in the genes of many organisms (human and mouse: 90%, drosophila: 60%) (1,2). AS contributes to cell differentiation, tissue identity and organ development (2). The expression of a specific isoform is often necessary to maintain tissue identity and function, while selection between alternative isoforms drives tissue development and cell differentiation (3). Understanding the role of AS in developmental processes requires the investigation of AS across different tissues during development. A number of studies aimed at revealing the importance of AS during development find that AS and specific isoform expression is frequent during early mouse embryonic development (4–6). In addition, in *Caenorhabditis elegans*, many alternatively spliced isoforms show a dramatic change in relative expression levels during embryonic to adult development (7). Studies targeted at the underlying mechanism of AS regulation have largely identified which splice motifs that interact with the splicing machinery to facilitate and regulate splicing. Several AS regulators that are critical to tissue development have been identified, such as CELF1 in heart development (8), ELAVL, PTBP1 and NOVA1/2 in brain development (9–11) and ESRP1 in liver development (12). However, as these elements are not sufficient to explain all aspects of AS regulation, including specific gene targeting, additional regulatory mechanisms must exist to direct the selection of alternatively spliced isoforms (13).

In addition to specific gene sequences, epigenetic mechanisms function in transcriptional regulation and play important roles in many biological processes (14,15). Genome regulatory elements undergo dynamic changes in the enrichment of histone post-translational modifications (hPTMs), which function during development to direct expression of corresponding genes (16,17). These hPTMs can function either as expressional activators, such as H3K4me3, or as repressors, such as H3K27me3 (18–20). Several hPTMs in a given promoter region act in a coordinated manner to regulate expression of genes necessary for specific cell differentiation during development. For example, co-enrichment of H3K4me3 and H3K27ac at enhancers related to heart development in mouse (21) regulate the expression of genes involved in developmental transitions in the cardiac lineage (22). In addition, computational analysis in several cell lines has found that particular hTPMs, such as H3K4me3, enriched around the transcriptional start sites of expressed genes associate with transcription initiation (23,24), while the levels of H3K4me3, H3K36me3 and H3K79me1 are associated with steady-state expression of particular exons and genes (24–26).

Beyond regulating gene expression, recent evidence suggests that hPTMs also function in the specification of exons spliced into a transcribed gene (27,28). Specific hPTMs regulate exon splicing by either directly recruiting splicing factors and adapters or indirectly modulating the elongation rate of RNA polymerase II (RNAPII), indicating a potential link between hPTMs and alternative splicing (28,29). Studies on human datasets show that distinct hPTMs are associated with exon inclusion or exclusion. A recent study in human stem cells shows that histone H3 lysine 36 trimethylation (H3K36me3) regulates alternative splicing events and is involved in nonsense-mediated mRNA decay of BARD1 (BRCA1-associated RING domain protein 1) (30). The authors also compare the contribution of genomic features and epigenetic features to alternative splicing and find that epigenetic features are more important to differentiate splicing patterns (30).

Due to the critical role of AS in tissue development and the potential link between specific hPTMs and AS in embryonic stem cell differentiation, we hypothesized that hPTMs could also drive development by regulating expression of alternatively spliced genes for specific processes in mammalian tissue development. To address this notion, we utilized a state-of-the-art machine learning approach to conduct a genome-wide analysis that related hPTMs to AS regulation during mammalian tissue development. We integrated ChIP-seq and RNA-seq data from 7 different mouse embryonic tissues at 6 developmental time points to determine (i) which hPTMs associate with alternatively spliced exons, (ii) which hPTM(s) most strongly predict alternative exon selection and (iii) the interaction of multiple hPTMs in exon selection. We analyzed the role of these hPTMs while controlling for cofounding factors originating from constitutive exon selection and gene expression level. We focused on one specific alternative splicing type – skipped exon – because it is the most prevalent alternative splicing event in mammalian tissue and contributes greatly to proteome diversity (31). We categorized two subtypes of skipped exons based on RNA-seq data analysis. Skipped exons were categorized as (i) ‘developmental gain/loss’ if the isoform switch occurred during development or (ii) ‘isoform selected high/low’ if the isoform was in the upper (75%) and lower (25%) quantiles, respectively, and isoform expression did not change over development. Enrichment analysis found these two groups of alternatively spliced genes consisted of different functional categories. We also observed that the number of AS events increased over developmental time, with brain tissue showing the greatest magnitude increase. To infer the relevance of hPTMs to AS events across tissues and development, we analyzed the ChIP-seq signal distribution of eight distinct hPTMs (H3K36me3, H3K4me1, H3K4me2, H3K4me3, H3K27ac, H2K27me3, H3K9me3 and H3K9ac) in the exon-flanking region. Remarkably, we found that only two hPTMs, H3K36me3 and H3K4me1, were differentially enriched with respect to skipped exon category.

We further derived a computational model for predicting skipped exon category using hPTM signal in the exon flanking regions. We found that hPTMs can accurately predict skipped exon category in both developmental gain/loss and isoform selected high/low groups, indicating the potential link between hPTM and skipped exon selection. Our findings indicated that specific histone modifications, H3K36me3 and H3K4me1, played a role in skipped exon selection among all the tissues and developmental time points examined, even when controlling for gene expression level. Furthermore, the contribution of some hPTMs was tissue-specific. In brain tissues and heart, H3K9ac had a relatively higher predictive rank, while in limb, neural tube and liver, the effect of H3K27me3 was higher. We also identified interactions of two or more hPTMs that highly predict AS. For example, the interaction between H3K36me3 and H3K4me1 in the exon flanking region was the top feature in both skipped exon categories. The other top interactions included H3K27me3/H3K36me3, H3K27ac/H3K36me3, H3K27ac/H3K4me1 and H3K36me3/H3K9me3. Collectively, our data demonstrated a link between hPTMs and alternative splicing in mouse tissue development, which will drive further experimental studies on the functional relevance of these modifications to alternative splicing.

## MATERIALS AND METHODS

### Dataset

We chose mouse embryonic tissue developmental data from ENCODE database (32), because both RNA-seq and ChIP-seq are available. We considered 7 tissues (forebrain, hindbrain, midbrain, neural tube, heart, liver and limb) from six time points (E11.5–E16.5 day). Supplemental Table S1 provides the full list of data analyzed. The analysis codes are available through github https://github.com/huqiwen0313/HM_splicing.

### Identification of alternative splicing exons in tissue development

Aligned BAM files (mm10) for all seven tissues from six timepoints were downloaded from ENCODE (32), each with two replicates. rMATS (version 4.0.1) was used to quantify ‘percent spliced in’ (PSI, exon inclusion level) and identify skipped exons that showed differential inclusion level (deltaPSI) between two time points (33). Skipped exons were divided into two different groups based on PSI and ΔPSI values: developmental gain/loss and isoform selected high/low. The developmental gain/loss group contains skipped exons which differ in inclusion level between two different time points in the same tissue. These exons showed an isoform switch behaviour during across developmental timepoints. For this group of skipped exons, we selected exons with ΔPSI ≥ 0.1 and FDR < 5% as gain class and exons with ΔPSI ≤ 0.1 and FDR <5% as loss class. For the isoform selected high/low group, we generated the global PSI distribution for all skipped exons. The upper (75%) and lower (25%) quantiles were used to divide exons into high class and low class. Skipped exons in this group did not change their inclusion across developmental timepoints. Rather, one isoform was consistently expressed higher or lower than the others across all timepoints.

### ChIP-seq data processing and hPTM profiling

ChIP-seq data (aligned BAM files, mm10) were downloaded from ENCODE database (32). For each tissue and time point, eight type of histone modifications, including H3K36me3, H3K4me1, H3K4me2, H3K4me3, H3K27ac, H3K27me3, H3K9ac and H3K9me3 were analysed. The global profiles of hPTMs among different groups of skipped exons were generated in two steps. First, for each exon, the flanking regions were defined as the 300 bp centered at acceptor and donor site, respectively, analysed in 15 bp bins. Second, the ChIP-seq reads were assigned to those binned regions and the normalized reads number for each binned region was calculated.

One caveat of this approach is that ChIP read count could be influenced not only by hPTM enrichment in ChIP-seq data, but by the overall accessibility of the region, antibody cross-reactivity, and many other technical considerations. To address this, we normalized ChIP-seq signals according to its library sizes. Ideally the ChIP data would be normalized to input. However, the related input data is not available from the ENCODE database.

To visualize the ChIP-seq signal pattern for different exon groups, we computed the average ChIP-seq signal and standard deviation across the flanking regions, averaged over all exons that belong to the same group. Constitutive exons of each tissue and timepoint were sampled from the same genes that contain alternatively spliced exons. To analyse the variability of hPTM enrichment by exon type, we generated heatmaps of the flanking regions from a pool of sampled exons that belong to each group in forebrain (Supplemental Figure S74). An ANOVA statistic was used to test if the signal distribution patterns were significantly different among different exon groups.

### Logistic regression and random forest modelling

To extract the features from hPTM distribution patterns, the flanking regions surrounding each splice site were divided into four regions: the intronic region at the acceptor splice site (5′ upstream, left_intron), the exonic region at the acceptor splice site (5′ downstream, left_exon), the exonic region at the donor splice site (3′ upstream, right_exon) and the intronic region at the acceptor splice site (5′ downstream, right_intron). The normalized ChIP-seq signals in those regions were calculated and considered as explanatory features for different types of hPTMs.

To demonstrate a predictive association between ChIP-seq signal and skipped exon groups, we constructed binary classification models. We chose two different models: logistic regression and random forest. Logistic regression is a type of probabilistic statistical classification model that measures the relationship between categorical response variable and explanatory variables, which can be formulated as below:

$$\begin{equation*}y\ = \frac{1}{{1 + {e^{\mathop \sum \nolimits_i {\beta _i}{x_i}}}}}\ \end{equation*}$$

in which *x_i_* is the ChIP-seq signal for certain type of hPTM, *y* is skipped exon groups and *β_i_* is the regression coefficient.

Random forest is an ensemble tree-based algorithm that uses bootstrap resampling to grow multiple decision trees and combines their results. The advantage of logistic regression and random forest over the other models is the interpretability of the model results, that is, we can know the effect of an individual feature to the response variable.

The model performance was measured by 5-fold cross validation, in which the entire dataset was randomly partitioned into five equal-sized subsamples. One subsample was used to evaluate the model performance (test set) and the remaining subsamples (training set) were used to train the model. The whole process was repeated by five times. Average model accuracy and ROC value were then calculated.

To generate statistical robustness, for each training set, the model was further tuned by a grid of parameters based on internal 3-fold cross validation. The model with the lowest error rate was then selected. For logistic regression, in order to achieve better performance, LASSO was applied to reduce the dimension of feature space. When the feature space is large the ordinary least square estimates generated by logistic regression may lead to large variance for the estimates, which will reduce the accuracy of prediction. We estimated the LASSO parameter λ through 3-fold cross validation. For each cross validation, a grid of λs was fed to the model. The corresponding prediction was estimated according to the test set. The λ value that minimized the overall prediction error was selected.

To test the different enrichment patterns of hPTMs in alternatively spliced exons, we built a second random forest model that included constitutive exons. The normalized ChIP-seq signals from the flanking regions of constitutive exons were calculated based on the same criteria as alternatively spliced exons. Constitutive exons were then bootstrapped to match the examples of alternative spliced exons. Model performance was evaluated by 5-fold cross validation with accuracy, macroPrecision, macroRecall and macroF1 scores.

### Controlling for gene expression level

Studies of the relationship between hPTMs, transcriptional regulation and gene expression find that hPTMs are associated with gene expression level (34–36). To control the effect of gene expression level that may cofound our findings, we stratified gene expression of the alternative spliced exons into three categories: high (the upper 25% quartile according to the entire gene expression level in the sample), medium (25–75% quartile) and low (the bottom 25% quartile). We then built a second model, using the random forest approach described above. In this case, for each category, we randomly divided the entire dataset into five subsamples, with one subsample used for testing and the rest of the four subsamples used for training. The ChIP-seq features in the exon franking regions were fed into the model to learn the representative features that differentiate exon splicing patterns. The model was then trained by 3-fold internal cross-validation based on the training set to select the model with the lowest error rate. The selected model was applied to the test set and the importance score for each hPTM was calculated. The entire process was repeated five times and the hPTMs with the top 5 highest average importance scores were selected and plotted.

### Iterative random forest modelling and interaction analysis

Iterative random forest model searched for high-order interaction in three steps: (i) Iteratively re-weighted random forests; (ii) extract decision rules from feature-weighted random forest path and recover interactions; (iii) bagging step to assess the stability of interactions. We trained iterative random forest model using R package iRF (https://github.com/sumbose/iRF), with number of iterations = 10 and number of bootstraps = 30. The stability score was estimated through 5-fold cross validation. Interactions with stability score >0.5 were considered as meaningful interactions.

### Gene ontology and motif enrichment analysis

The Gene Ontology (GO) enrichment analysis was performed using DAVID (37) under default parameters. Overrepresented GO terms for GO domain belong to biological process, cellular component and molecular function were used to generate enrichment datasets based on FDR cutoff 0.05.

We further explored the potential sequence features that may relate to regulation of spliced exons. Enriched motifs in the exon flanking regions of spliced exons in each tissue and timepoint were identified through MAPS2 (http://rmaps.cecsresearch.org/MTool/). Motifs with *P*-values smaller than 0.01 in all flanking regions of spliced exons were extracted. Heatmap of enriched motifs were generated based on log *P*-values using heatmap function in R.

## RESULTS

### Characterization of alternative splicing events in tissue development

**Figure 1.**
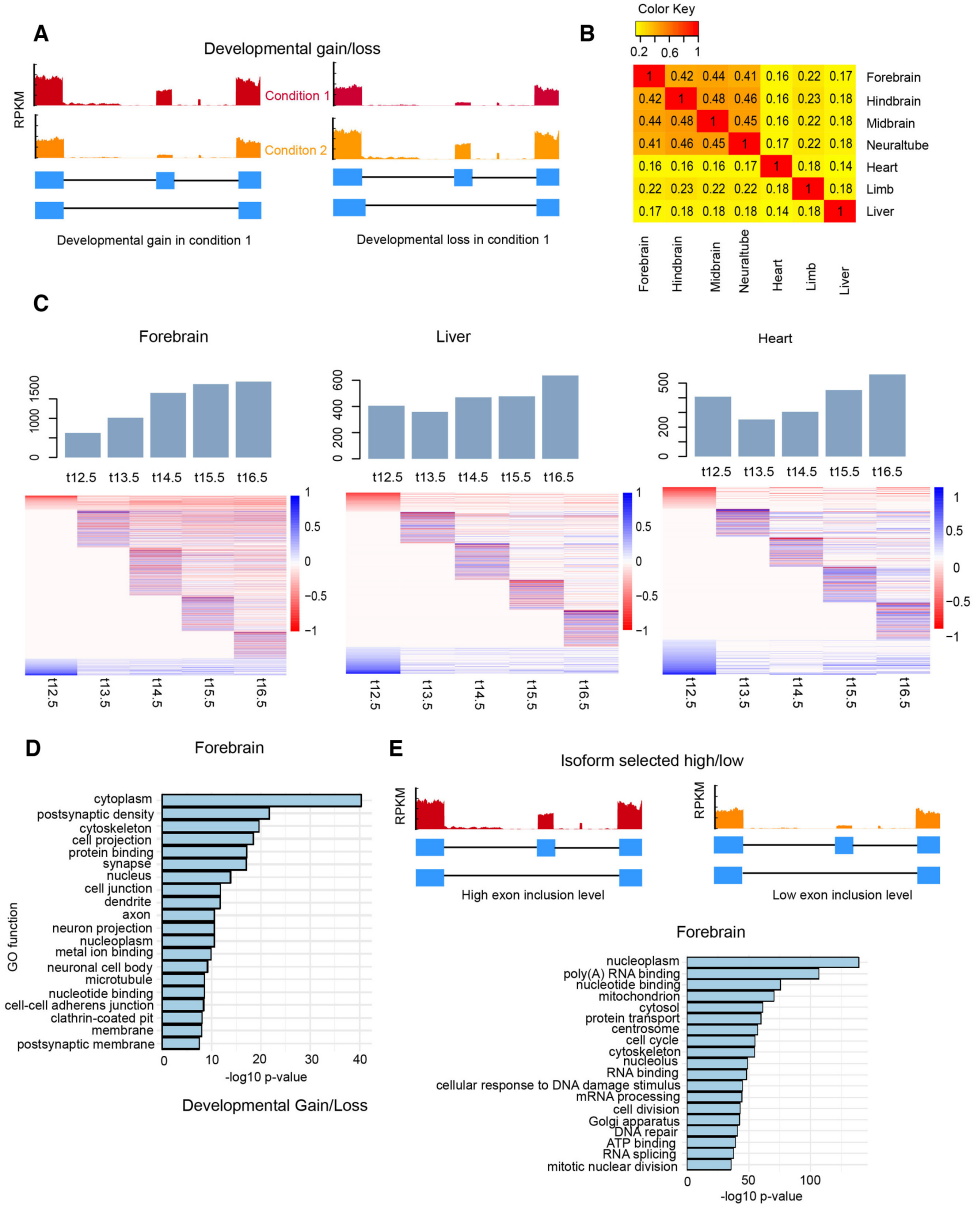
Alternative splicing events associated with tissue development. (**A**) Schematic illustrating developmentally associated skipped exons. (**B**) Overlap of developmentally associated alternative splicing events between tissues (**C**) Number of significant alternative splicing events identified across 7 developmental time points for several mouse tissues. Heat maps show the differential exon inclusion level (ΔPSI) by comparing each time point to earliest time point in skipped exons (row). Bar graph shows the proportion of lineage-specific AS genes. (**D**) Ontology analysis of developmentally associated AS genes in forebrain. Data are derived from ENCODE database (32) (Supplemental Table S1) and analysed using rMATs (33). (**E**) Ontology analysis of isoform selected high/low AS genes in forebrain.

Alternative splicing has been shown to contribute to cell differentiation, tissue identity and organ development (2,38). To identify AS events associated with tissue development, we analyzed ENCODE RNA-seq data (32) derived from mouse embryonic tissues at multiple developmental time points. We selected data from 7 tissues at 6 time points based on the availability of both RNA- and ChIP-seq data. Our analysis focused on one specific AS type—skipped exon—because it is the most common type in the mammalian transcriptome (31). Significant skipped exon events in each dataset were identified by comparing each time point with the earliest time point (E11.5) using rMATS (33). We analysed alternative splice events over developmental time for each tissue and identified skipped exons with significant ΔPSI larger than 0.1 (developmental gain) or less than –0.1 (developmental loss) (FDR < 0.05). These skipped exons associated with tissue development are referred to as ‘developmental gain/loss,’ and vary in number from 600 to 3000 across the tissues examined (Figure 1A, Supplemental Table S2).

We observed that the number of developmentally associated alternative splicing events increased with developmental time, with brain tissues showing the greatest increase (Figure 1C, Supplemental Table S2, Figure S1). For example, the number of alternative splicing events in forebrain increased by 310.56%, from 625 on E12.5 day to 1941 on E16.5, while in liver the increase was only 157.3% (405–637). Hierarchical clustering on skipped exon events across developmental time points revealed specific splicing patterns in different tissues (Figure 1C, Supplemental Figure S1). In brain, neural tube and limb, there were more developmental gain events after E12.5, while in heart and liver, the number of developmental loss events was slightly higher at most time points (Supplemental Table S2). Tissue-specific alternative splicing plays important roles for tissue identity during development (39). Thus, to explore what percentage of developmentally-associated skipped exons are tissue-specific, we performed pairwise comparison of the identified skipped exons among different tissues (Figure 1B). On average, over half of the lineage-specific transcripts in each tissue were alternatively spliced; this percentage was not significantly different between tissues (Supplemental Figure S1). In addition, we found most of the lineage-specific events occurred in the early time point, which account for ∼50% of those events among all tissues. Brain tissues showed a significant decrease of lineage-specific events at later time points when compared to liver, limb and heart (Supplemental Figure S1). This finding underscores the relevance of alternative splicing to lineage-specific gene expression.

In addition to developmentally associated skipped exons, we also observed another category of skipped exons according to PSI values derived from rMATS – isoform selected high/low (Figure 1E). These exons were alternative spliced but did not show inclusion level changes over developmental time.

Gene ontology (GO) enrichment analysis found that these two categories of AS genes were enriched in different functional categories (Figure 1D, E, Supplemental Table S3). AS genes belonging to the developmental gain/loss category were overrepresented in certain GO functions, such as cytoplasm, postsynaptic density and cytoskeleton (Figure 1D), consistent with previous studies of AS genes in mouse tissue development (40,41). Alternatively, AS genes belonging to the inclusion high versus low category were enriched in RNA binding, cell cycle and cell division GO functions (Figure 1E). Taken together, these results comprised a global analysis of alternative splicing events in different tissues across development.

### Histone modification enrichment in exon flanking regions differentiated skipped exon groups

Though previous studies find that histone modifications are enriched in promoter regions and predict expression of corresponding genes (24,26,42), it has become increasingly clear that they also associate with gene bodies and exon regions, indicating a potential role of histone modifications in pre-mRNA splicing regulation (43). To investigate if histone modifications associated with alternative splicing across tissue development, we focused on the ChIP-seq distribution patterns of 8 histone modifications, including H3K4me1, 2, 3, H3K9me3, H3K27me3, H3K36me3, H3K9ac and H3K27ac, which were available for all tissues and developmental time points analysed. For each developmental time point, we profiled the hPTM distribution patterns of all skipped and its paired constitutive exons. We reasoned that histone modifications related to alternative splicing are likely to be localized to the genomic region at which splicing occurs and hypothesized that ChIP-seq distribution patterns would vary by skipped exon category. Thus, we compared the normalized ChIP-seq signal distributions of each hPTM in a ±150 bp region flanking the splice sites of each skipped exon. The distributions of all eight hPTMs in all seven tissues at each developmental time point are in s 2–31.

**Figure 2.**
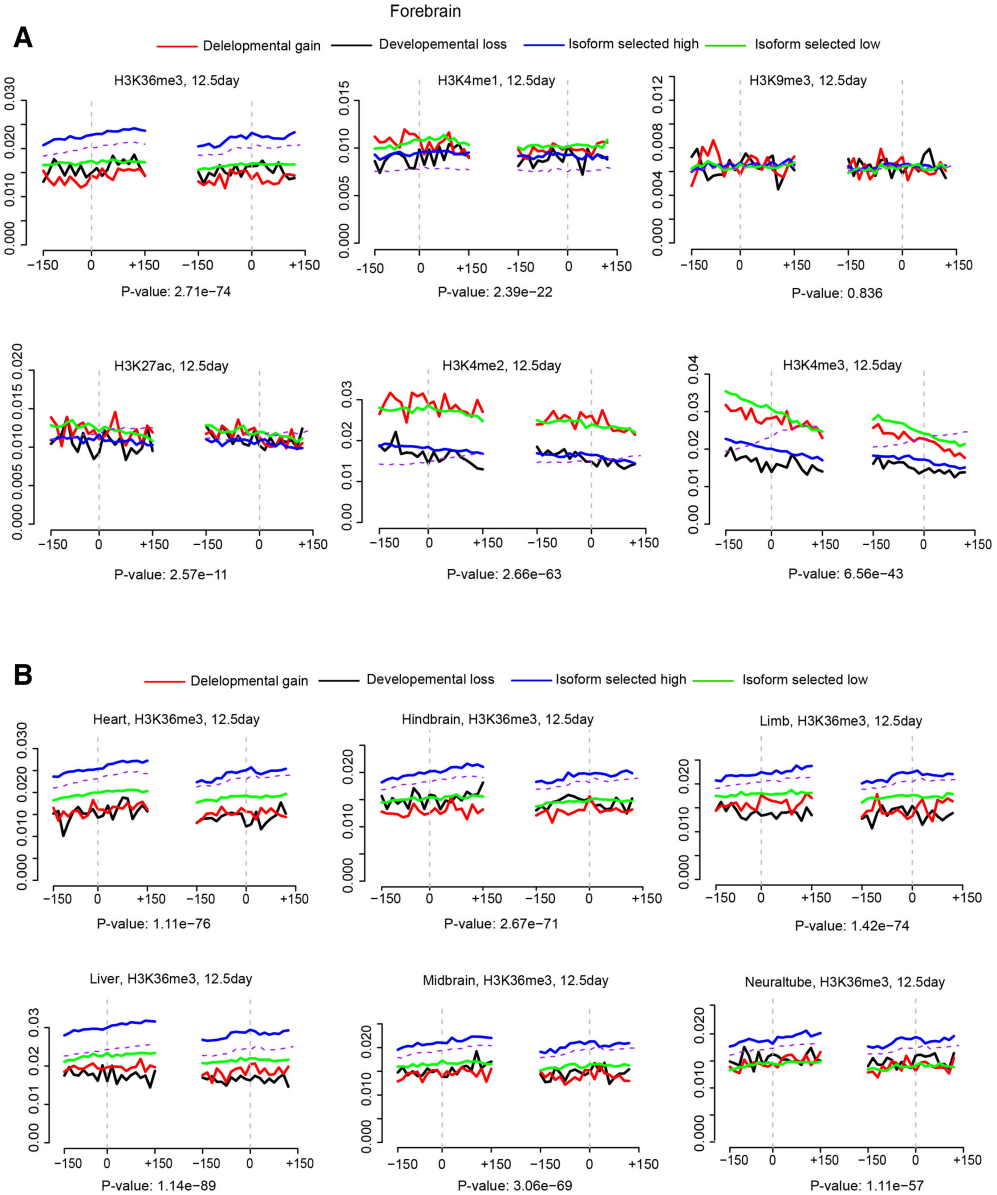
Representative distribution of mean ChIP-seq signal of 6 types of hPTM, including H3K36me3, H3K4me1, H3K9me3, H3K27ac, H3K4me2 and H3K4me3 on the flanking region (±150 bp) of four types of skipped exons. Dashed grey line shows exon–intron borders. (**A**) Forebrain, E12.5 (**B**) distribution of H3K36me3 among six tissues shows hPTM distribution was significantly different among different types of skipped exons (*P*-values, ANOVA test).

Figure 2 shows the mean ChIP-seq signal distributions of several hPTMs in brain and heart. We found that only certain modifications, H3K36me3 and H3K4me1/2/3, distributed according to skipped exon category. In addition, hPTMs corresponding to different groups of skipped exons diverged greatly with respect to their correlative behaviour. For example, H3K36me3 was positively correlated with exon inclusion levels of skipped exons in the isoform selected high versus low inclusion category. That is, the higher the exon inclusion level, the stronger the H3K36me3 enrichment in the exon flanking regions. However, H3K4me2/3 displayed the opposite trend, that is, the H3K4me2/3 enrichment was highest in skipped exons with low inclusion level. This association pattern was consistent among all tissues (Figure 2, Supplemental Figure S2–S31). Conversely, for the gain versus loss inclusion category, the association patterns were similar in brain tissues but differed among the other tissues. This is especially true for H3K4me2/3, as we found that in forebrain at E12.5, H3K4me2/3 enrichment was positively correlated with skipped exons with inclusion gain, but in heart, limb and liver, enrichment appeared to be negatively correlated with those exons. The distribution of hPTMs also significantly different from the distribution of their paired constitutive exons in majority of the cases.

Comparison of hPTM distribution across different time points and tissues revealed unique patterns for some hPTMs (Supplemental Figures S2–S31). When we compared different tissues at the same time point, H3K4me1/2/3 enrichment displayed the biggest variation, e.g. H3K4me3 signal was higher in the exon flanking regions of inclusion gain versus loss category in forebrain at E15.5, while in heart, it was much higher at exons in low versus high category. H3K4me also varied across different time points for some tissues. For example, in heart tissue at E12.5, H3K4me3 enrichment was greatest for exons in the inclusion gain versus loss category, but this preferential enrichment gradually switched to exons in the high vs. low group over developmental time. These results suggested that the role of of hPTMs in AS varies across time points and tissues.

### Modelling skipped exon inclusion by logistic regression and random forest

We next took our analysis one step further to computationally model the relationship between histone modifications and each skipped exon category. We tested the hypothesis that the model can distinguish two different groups of skipped exons: (i) exons with developmental gain versus exons with developmental loss and (ii) exons with isoform selected high versus exons with isoform selected low. In this study, we chose two different approaches—logistic regression and random forest. To avoid the uncertainty and complexity of using deep learning models, we chose to use traditional machine learning approaches because of their good performance and ease of training and interpretability (see Discussion).

For each histone modification, we summed the ChIP-seq signal upstream and downstream (±150 bp) of skipped exons’ splice sites and regarded them as eight hPTM features of the model. These eight features were then expanded to 32 explanatory variables to build the model. The model performance was measured by accuracy and area under the ROC curve (AUC) based on 5-fold cross validation.

**Figure 3.**
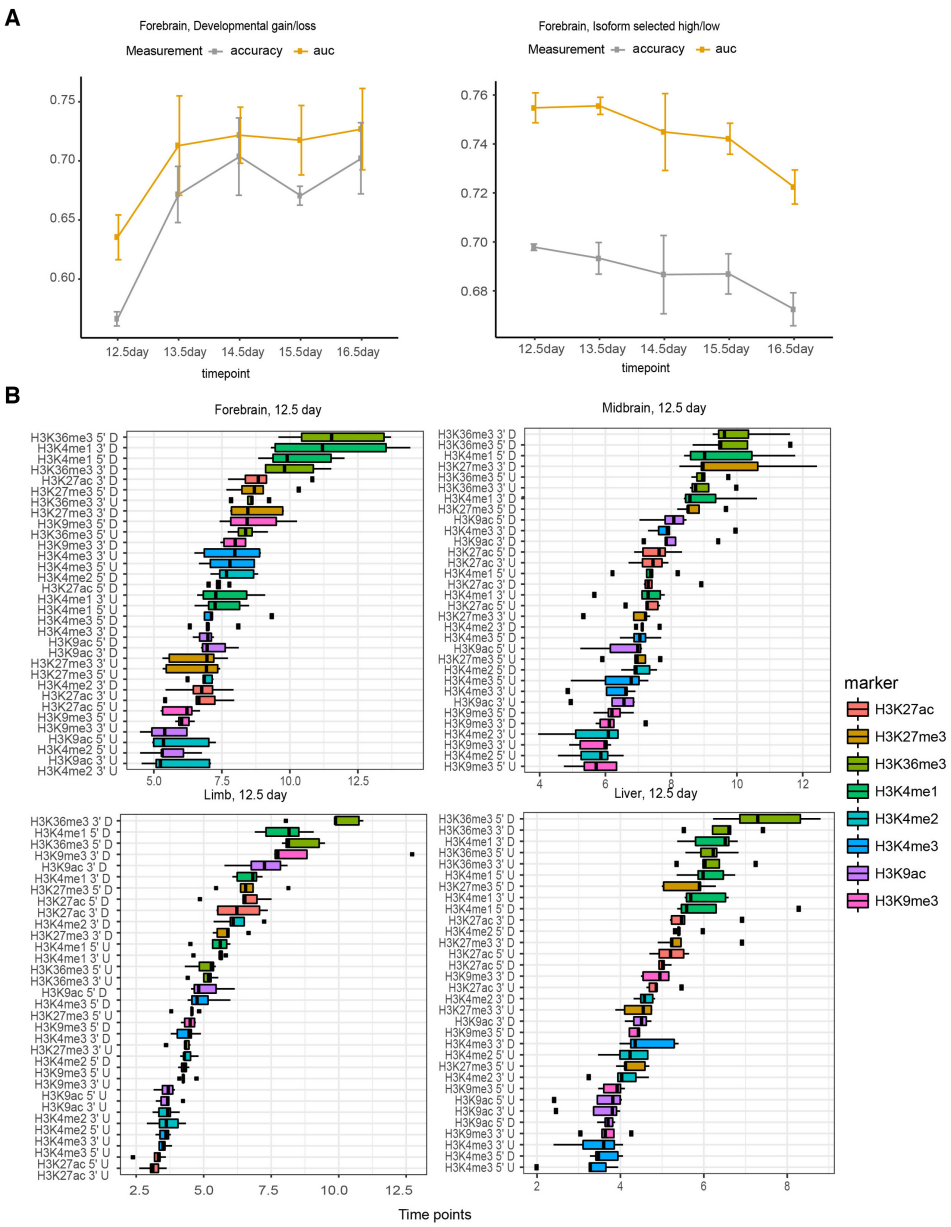
Model performance and important histone modifications associated with skipped exon selection in different tissues and timepoints (**A**) Accuracy and AUC values of random forest models built based on hPTM signals on the flanking regions of skipped exons in forebrain across developmental time points. (**B**) H3K36me3 and H3K4me1 are the most predictive hPTM in differentiating skipped exon inclusion categories. Boxplot of important score generated by random forest model in different tissues at E12.5 shows several types of hPTM are key predictors. Importance score is calculated based on 5-fold cross validation.

Table 1 shows the accuracy values of two models for seven tissues at different developmental time points. In general, random forest showed better performance than logistic regression in all tissues and time points, which was consistent with a recent study that compared the performance of 13 popular machine learning algorithms (44). The accuracy of random forest model varied from 0.57 to 0.72 in developmental gain versus developmental loss category and from 0.67 to 0.70 in isoform selected high versus isoform selected low category. Due to the imbalanced datasets of some tissues, we also compared their AUC values, which is insensitive to imbalanced classes. Consistent with accuracy values, AUC of random forest was between 0.64 and 0.74 in developmental gain versus developmental loss category and from 0.72 to 0.75 in isoform selected high versus isoform selected low category (Figure 3A, Supplement Figures S35–S37). In addition, accuracy and AUC values from random forest were much higher than random prediction (0.5), indicating a good predictive power of random forest model.

**Table 1.** Accuracies of logistic and random forest models to predict gain verus loss and high versus low categories for different tissues at each developmental time point. Accuracies were calculated based on 5-fold cross validation

**Gain versus loss**	Logistic regression					Random forest				
Tissue	12.5 day	13.5 day	14.5 day	15.5 day	16.5 day	12.5 day	13.5 day	14.5 day	15.5 day	16.5 day
Forebrain	0.53	0.59	0.64	0.6	0.62	0.57	0.67	0.7	0.67	0.7
Heart	0.62	0.62	0.5	0.62	0.6	0.62	0.64	0.64	0.64	0.62
Hindbrain	0.53	0.63	0.63	0.63	0.59	0.58	0.69	0.71	0.7	0.69
Limb	0.52	0.57	0.56	0.58	NA	0.61	0.61	0.67	0.64	NA
Liver	0.59	0.54	0.47	0.54	0.53	0.64	0.59	0.52	0.59	0.61
Midbrain	0.58	0.63	0.65	0.62	0.62	0.59	0.71	0.72	0.69	0.7
Neural tube	0.55	0.61	0.65	0.59	NA	0.59	0.67	0.72	0.68	NA
**High versus low**	Logistic regression					Random forest				
Tissue	12.5 day	13.5 day	14.5 day	15.5 day	16.5 day	12.5 day	13.5 day	14.5 day	15.5 day	16.5 day
Forebrain	0.63	0.64	0.63	0.63	0.61	0.7	0.69	0.69	0.69	0.67
Heart	0.61	0.63	0.61	0.62	0.62	0.69	0.7	0.69	0.7	0.68
Hindbrain	0.63	0.62	0.62	0.62	0.63	0.69	0.69	0.68	0.69	0.7
Limb	0.63	0.63	0.62	0.62	NA	0.69	0.69	0.67	0.68	NA
Liver	0.62	0.6	0.6	0.59	0.59	0.69	0.67	0.67	0.66	0.67
Midbrain	0.62	0.62	0.63	0.63	0.62	0.68	0.69	0.69	0.68	0.68
Neural tube	0.65	0.63	0.62	0.64	NA	0.7	0.69	0.67	0.7	NA

For developmental gain versus loss category, the model performance varied over time. The AUC of random forest was >0.6 for the majority of tissues and time points and showed a trend for increase over time, but this trend may be caused by the smaller sample size at early time points (Figure 3A, Supplemental Figures S32–S36). For the skipped exon in isoform selected high versus isoform selected low category, the model performance remained stable for all tissues and did not differ greatly when compared to most of the time points of gain versus loss inclusion category (Table 1, Figure 3A, Supplemental Figures S32–S36).

To further investigate the association patterns between hPTMs and skipped exons, we included constitutive exons in the random forest model. Similar to the previous model, the model performance of random forest exhibited the same patterns across different tissues and timepoints in both developmental gain/loss and isoform selected high/low categories. However, adding features from constitutive exons further increased the model performance in the developmental gain/loss exon category, while there was no effect on the isoform selected high/low exon category. This indicated greater divergence in the hPTM features between constitutive and developmentally-regulated alternative exons in the developmental gain/loss category (Supplemental Table S3).

In summary, our data indicated an association of hPTMs with two categories of skipped exon selection: exons that show isoform switching behavior during tissue development and exons that are alternatively spliced but without isoform switching over developmental time. In both categories, hPTMs were highly predictive of skipped exon inclusion, suggesting that hPTMs are involved in skipped exon selection, either directly or indirectly.

### Specific types of hPTMs were key predictors for skipped exon groups

To elucidate the relative importance of different hPTMs on skipped exon selection and to test their respective contributions to splicing across tissues and time points, we extracted the importance score generated from random forest model (Figure 3B, Supplemental Figures S37–S51). Overall, H3K36me3 and H3K4me1 were the most predictive hPTMs in differentiating skipped exon inclusion categories, while H3K9me3 was the least informative. In addition to H3K36me3, we observed a strong predictive effect of H3K4me1 at 5′ splice site downstream and 3′ splice site upstream in many of the tissues. The 3′ splice site upstream of H3K27me3 showed a greater contribution in midbrain and hindbrain at E12.5, while in limb, H3K9ac and H3K9me3 at the 3′ splice site upstream were informative to differentiate skipped exon groups.

We next compared the same tissue at different developmental time points, and similarly found that H3K36me3 and H3K4me1 ranked at the top for majority of the cases (Supplemental Figures S37–S51). Consistent with their contributions at E12.5, the 5′ splice site downstream, 3′ splice site upstream of H3K36me3 and the 5′ splice site downstream, 3′ splice site upstream of H3K4me1 were the most informative predictors. On the other hand, the contribution of some types of hPTMs varied over time. For example, in liver at E13.5, the 3′ splice site upstream of H3K9ac had a much stronger predictive effect when the same region was compared at the other time points.

**Figure 4.**
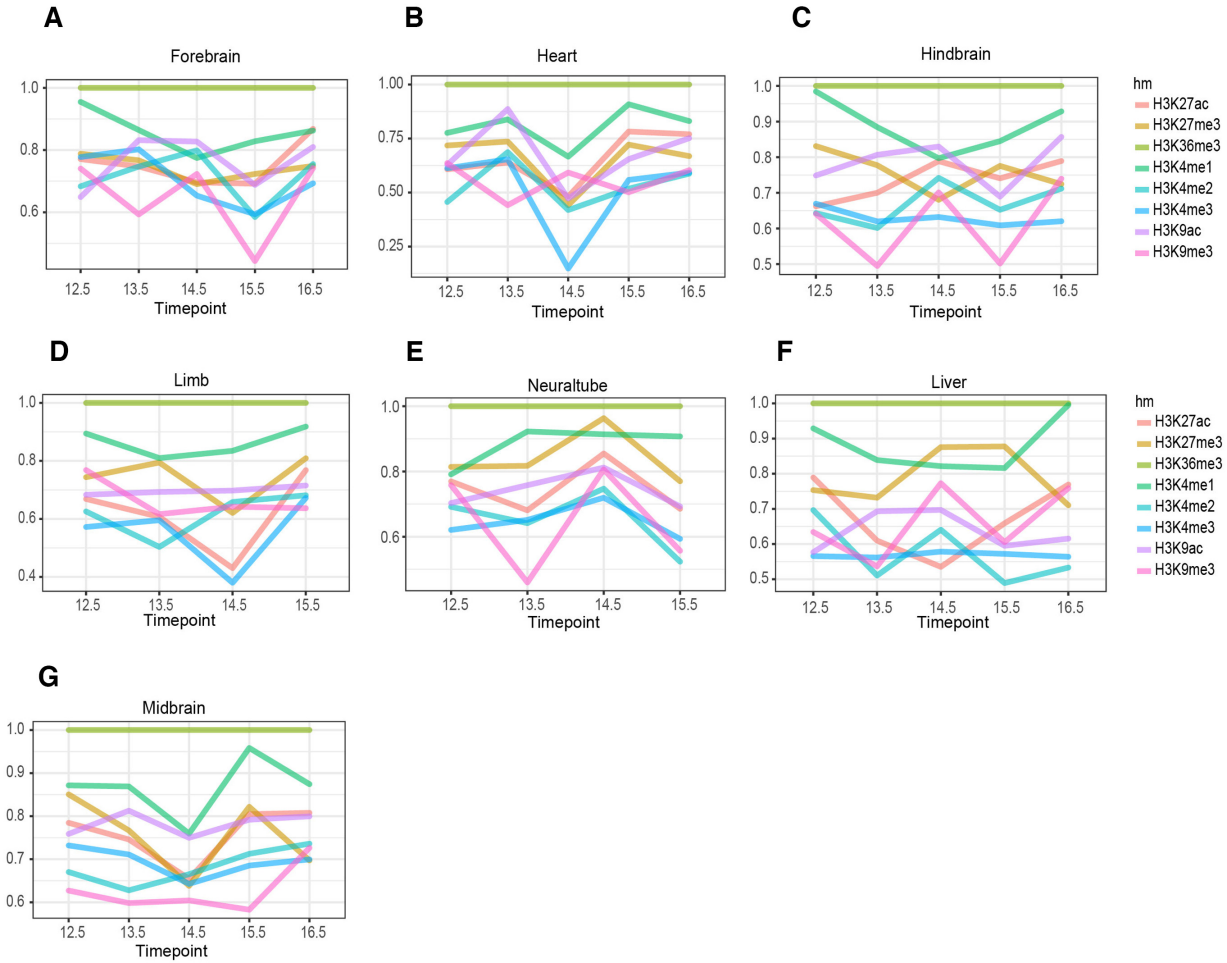
Contributions of different types of hPTM to differentiate developmental gain versus developmental loss over time in (**A**) forebrain (**B**) heart (**C**) hindbrain (**D**) limb (**E**) neuraltube (**F**) liver (**G**) midbrain. The importance score generated by random forest was normalized so that the maximum value is 1. H3K36me3 is the top predictive feature across all the timepoints and tissues.

To further examine the contribution of individual hPTMs, we averaged the important score in the flanking regions for each hPTM and normalized it by dividing the largest averaged value. We then plotted the normalized score for each time point. Figure 4 shows the contribution of each hPTM to differentiating exons in the developmental gain/loss category. We observed a consistent subset of hPTMs as predictors for all tissues and time points examined, with H3K36me3 being the most informative feature in 100% of cases and H3K4me1 being the second most informative feature in ∼80% of cases. The contribution of other hPTMs varied across the different tissues examined. In brain tissues and heart, H3K9ac had a relatively higher predictive rank, while in limb, neural tube and liver, the effect of H3K27me3 was greatest. The pattern was consistent for isoform selected high vs. low group (Supplemental Figure S5 and S52–S53).

**Figure 5.**
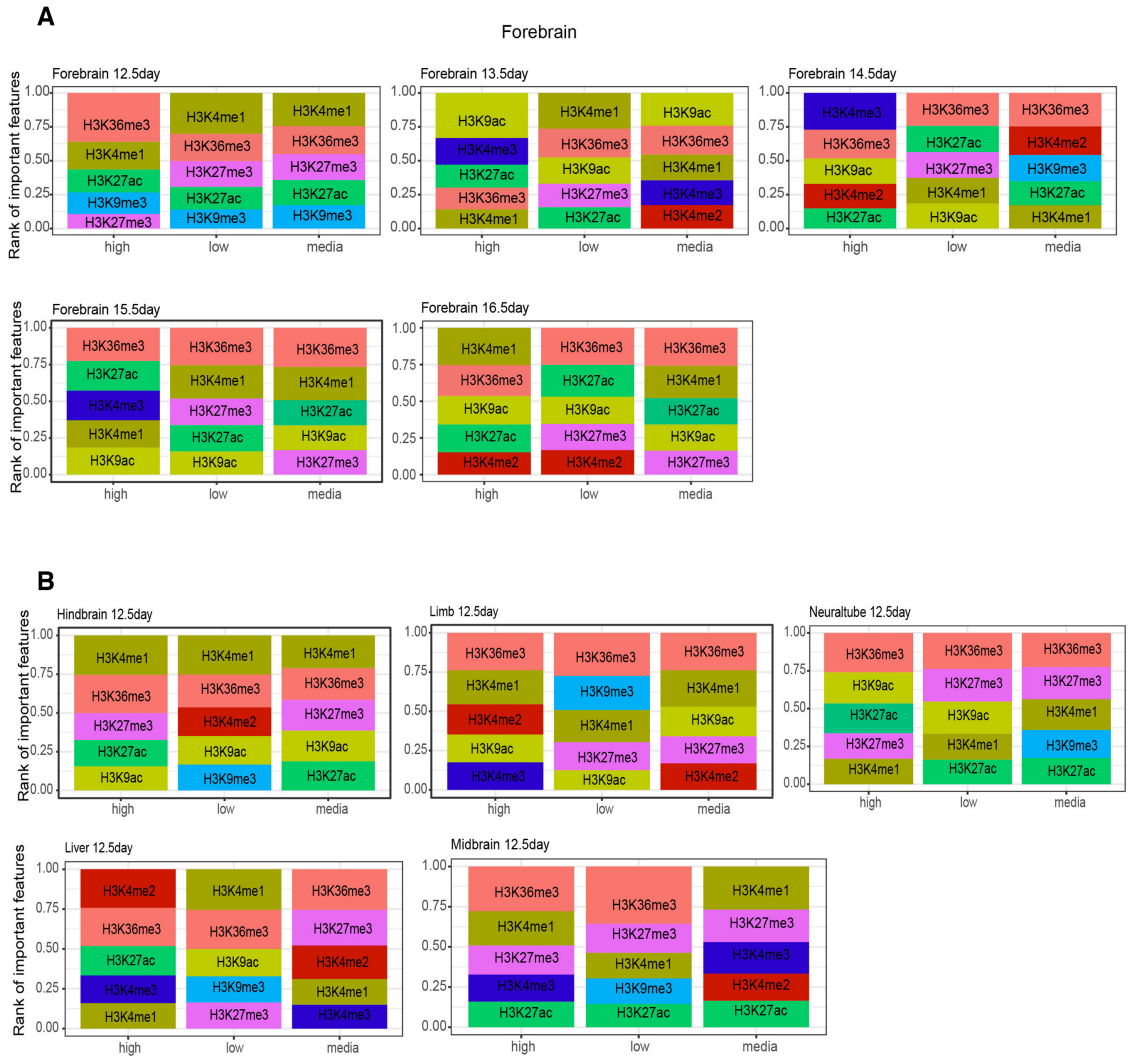
Contribution of top 5 predictive hPTMs after stratifying gene expression level in developmental gain/loss exons (**A**) forebrain, E12.5 (**B**) Contribution of different hPTMs among six tissues shows H3K36me3 and H3K4me1 consistently become the top predictive features to differentiate different exon splicing patterns.

A multitude of studies have detailed the relationships between hPTMs and gene expression levels—both in promoter regions and within gene bodies (24–26,45). Gene expression levels may therefore also be correlated to skipped exon usage and confound our model result. Although we observed little correlation among hPTM signals, exon splicing and gene expression (Supplemental Table S5, Figure S73), to control for the potential confounding effect of gene expression on model accuracy, we further divided the exons into three categories according to their gene expression level: high, medium, low (see Materials and Methods). We found that while the contributions of hPTMs varied by gene expression category, H3K36me3 and H3K4me1 were consistently the top predictive features (100% of all tissues and timepoints for H3K36me3 and 86% of tissues and timepoints for H3K4me1) (Figure 5, Supplemental Figures S68–S71). The predominant appearance of H3K36me3 and H3K4me1 as top predictors indicated their strong association with exon splicing patterns in both developmental gain/loss and isoform selected high/low categories.

To further investigate the role of these hPTMs we selected several developmentally-regulated alternative exons characterized in the literature (38). We then analyzed the hPTM enrichment in the flanking regions of these exons. Using this approach, we identified a subset of exons for which hPTM enrichment strongly correlated with exon splicing rate, as measured by PSI. This data further supported a potential link between hPTMs and tissue development driven by alternative splicing (Supplemental Table S4). Taken together, these results supported our hypothesis that H3K36me3 and H3K4me1 specifically contribute to alternative splicing.

### Interaction among histone modifications and skipped exon selection

Interactions among histone modifications in promoter regions for the regulation of gene expression and exon splicing have been reported in several studies based on Bayesian methods (23,46). However, Bayesian methods used in these studies discretized the ChIP-seq signal based on the clustering result, which may cause information loss. To investigate the interaction among histone modifications, we used iterative random forest model (iRF), which can be applied to identify high-order interactions. iRF algorithm first sequentially grows feature-weighted random forests to perform feature space reduction and then fits the model based on Random Intersection Trees algorithm to identify high-order feature combinations that are prevalent on the random forest decision paths (55).

**Figure 6.**
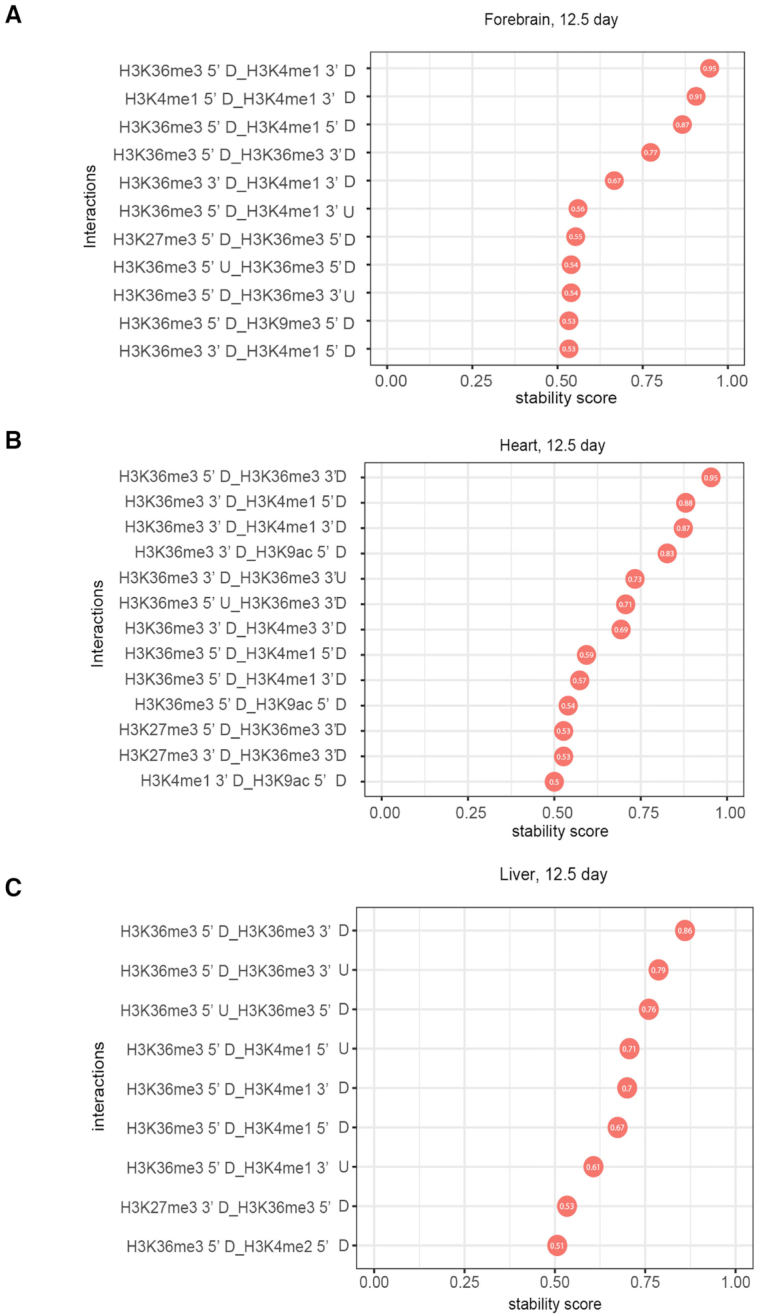
Top stable interactions of histone modifications in the exon flanking region for gain versus loss group obtained by iRF algorithm (stability score > = 0.5). 3 tissues (12.5 day) were visualized: (**A**) forebrain, (**B**) heart and (**C**) liver. Many histone modification interactions were observed in forebrain, heart and liver for developmental gain versus loss category at E12.5. These included interactions between modifications on different amino acids (e.g. H3K36me3 and H3K4me1), between different kinds of modifications (e.g. H3K4me1 and H3K9ac), and between the different genic regions of the same histone modification (e.g. H3K4me1 5′ downstream and H3K4me1 3′ upstream).

As demonstrated in Figure 6, many histone modification interactions were observed in forebrain, heart and liver for developmental gain versus loss category at E12.5. The interactions from other tissues and isoform selected high versus low category are in Supplemental Figures S54–S67. These included interactions between modifications on different amino acids (e.g. H3K36me3 and H3K4me1), between different kinds of modifications (e.g. H3K4me1 and H3K9ac), and between the different genic regions of the same histone modification (e.g. H3K4me1 5′ downstream and H3K4me1 3′ upstream). The interaction between H3K36me3 and H3K4me1 in the exon flanking regions (H3K36me3 5′ downstream and H3K4me1 3′ upstream) was the top feature in both developmental gain versus loss and isoform selected high versus low group. The other top interactions included H3K27me3 and H3K36me3, H3K27ac and H3K36me3, H3K27ac and H3K4me1 and H3K36me3 and H3K9me3. Interestingly, we observed many interactions between the different flanking regions of the same histone modification, such as interactions between H3K36me3 5′ upstream and H3K36me3 3′ upstream, suggesting a spatial relevance of hPTMs to alternatively spliced exon selection. The prevalence of H3K36me3 and H3K4me1 as interacting partners further underscores the relative importance of these hPTMs in skipped exon selection.

## DISCUSSION

AS plays a critical role during tissue development and cell differentiation (2,38). Previous studies reveal several regulatory mechanisms for AS, including expression and targeting of splicing factors and enrichment of hPTMs (38). In this study, we sought to comprehensively investigate previous observations of the relationship between hPTMs and AS during tissue development by integrating ChIP-seq and RNA-seq data from seven different mouse embryonic tissues at six developmental time points. We identified two different categories of AS (skipped) exons: skipped exons associated with development and skipped exons associated with isoform selection. Ontological analyses found that genes from these two categories are enriched in different functions. AS genes associated with developmental gain/loss category in forebrain were more likely to be enriched in neuronal-related functional categories, such as neuron projection, postsynaptic density and cytoskeleton. This is consistent with previous gene ontology analysis for differentially spliced exons in developing cerebral cortex, which show that cytoskeleton genes are overrepresented in mouse and human (41). On the other hand, AS genes that belong to isoform selected high/low category are overrepresented in distinct ontological categories important to maintain cell function or tissue identity, such as cell cycle, protein transport and RNA binding.

Computational models constructed based on ChIP-seq signal in the flanking regions of skipped exons showed that hPTMs associated with both categories of skipped exon. Consistent with previous studies, we found H3K36me3 to be most predictive for skipped exon groups (37,41). Specifically, H3K36me3 enrichment in the flanking region 5′ splice site downstream and 3′ splice site upstream of the skipped exon was the top predictor in all tissues. This result was also true when we stratified the exons by gene expression level. While the contributions of hPTMs varied by gene expression category, H3K36me3 was consistently the top predictive features in all tissues and time points (Figure 5, Supplemental Figure S68–S71). This result has been reported previously in other systems. First, a computational analysis of skipped exons based on 3 human cell lines shows the enrichment of H3K36me3 in exon and downstream of 3′ splice site is significantly correlated with skipped exon inclusion (43). Second, analysis of human and C. elegans exons finds H3K36me3 is most highly enriched at the 5′ end of exons (47). Finally, our previous analysis of mouse nucleus accumbens reveals that H3K36me3 has the greatest enrichment at alternative isoforms relative to other hPTMs (48). Our approach is an improvement over previous approaches based on clustering (49) by quantifying the global associations between hPTMs and exon splicing and their contributions. Together with our findings, these prior reports indicate one possible regulatory by which H3K36me3 enrichment contributes to alternative exon selection in AS.

Using iterative random forest model, we further identified interactions between several hPTMs that associated with skipped exon selection. The interaction between H3K36me3 and H3K4me1 in the exon flanking regions was the top feature in both developmental gain/loss and isoform selected high/low group. Other interactions, such as H3K27me3 and H3K36me3, H3K27ac and H3K36me3, and H3K36me3 and H3K9me3, indicated that relatively weaker predictive hPTMs may only be functional when in combination with the highly predictive ones. The concept of hPTM interaction is not new. Several studies have found the combinatorial effect of histone modifications and their association with gene transcription and differentiation. For example Han *et al.* deciphers histone modification interaction relationships on exons based on Bayesian network (23). Interestingly, we found many interactions that occur between the different flanking regions of same or different histone modifications, such as interactions between H3K36me3 5′ upstream and H3K36me3 3′ upstream and between H3K36me3 5′ downstream and H3K4me1 3′ upstream. These results suggested hPTMs located in different positions in the exon flanking region may contribute differently to skipped exon selection. This is consistent with the result of a previous study that finds hPTMs correlate to skipped exon inclusion via specific patterns along the flanking region of those exons (25). In particular, H3K36me3 shows a significant correlation between upstream and downstream of exon flanking regions for exon inclusion rate, which is consistent with our finding.

**Figure 7.**
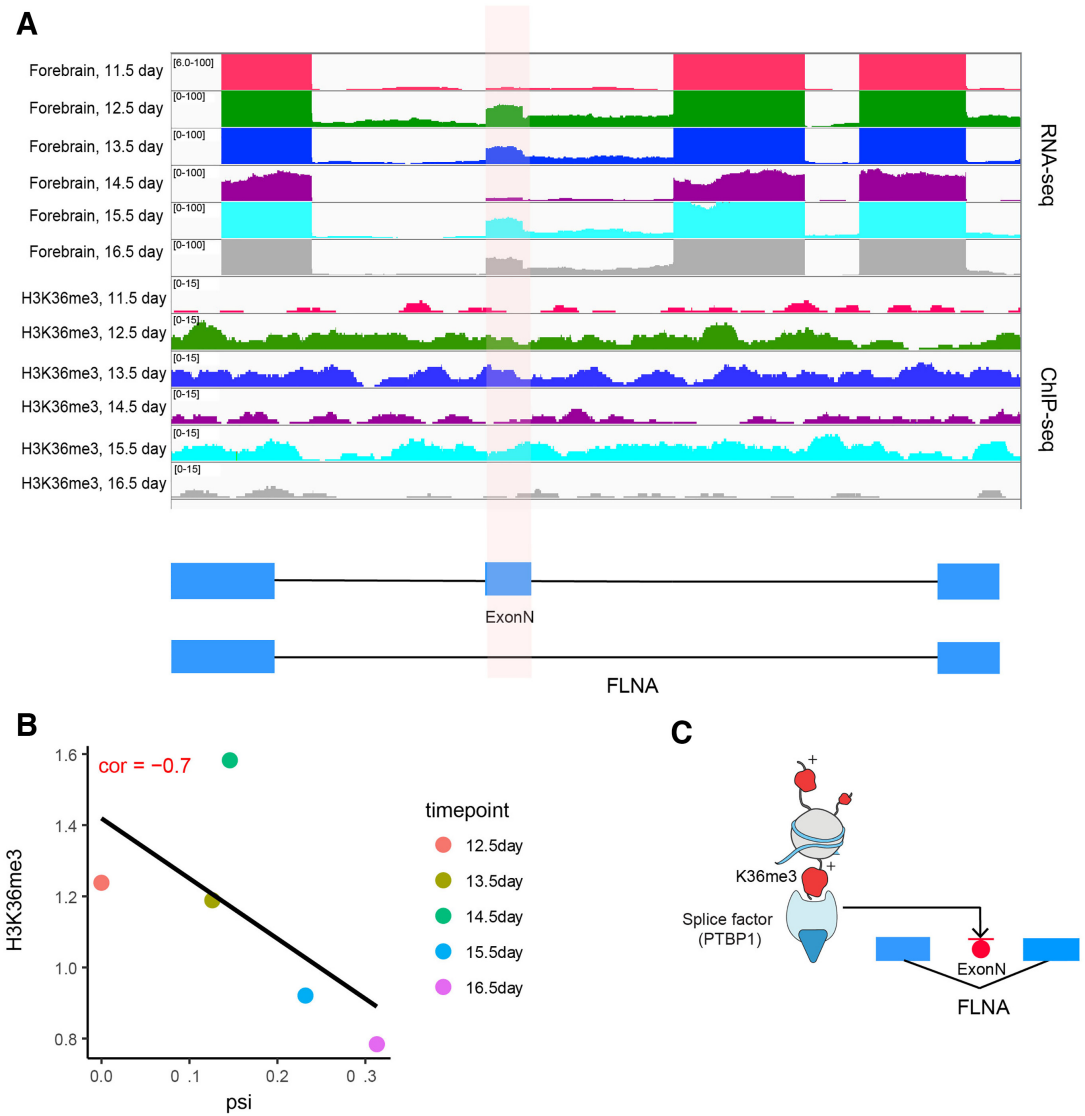
Potential mechanistic connection between histone modifications and tissue development. (**A**) Genome browser view shows the enrichment of H3K36me3 and exonN inclusion in forebrain, heart, liver and limb. (**B**) The inclusion level of exonN in FLNA gene was significantly correlated with H3K36me3 enrichment. (**C**) Schematic depicts a potential mechanism by which H3K36me3 regulates exonN inclusion. H3K36me3 can recruit splicing factor PTBP1, which will further repress exonN inclusion.

Furthermore, we observed the occurrence of some hPTMs at several skipped exons found in neuronal developmental tissues from a previous study (38), suggesting a potential mechanistic connection between those modifications and tissue development driven by AS (Supplemental Table S4). RNA-seq analysis from Zhang *et al.* shows exonN is included in cerebral cortex and cerebellum but excluded from non-neural tissues. This is consistent with our finding that the inclusion level of FLNA exonN is significantly increased in forebrain over developmental time, but not in liver, heart and limb. Interestingly, one previous study finds that mutations that disrupt the Polypyrimidine tract binding protein (PTBP1) binding site of FLNA exonN in neural progenitor cell causes a brain-specific malformation in human, suggesting the potential regulatory role between PTBP1 and exonN inclusion (41). In this study, we observed that the signal of H3K36me3 in the flanking regions was significantly correlated with FLNA exonN inclusion in forebrain (Figure 7A, B), suggesting a link among histone modifications, splice factors and exon inclusion (Figure 7C). This notion is furthered by previous findings from Luco *et al.*, that H3K36me3 can directly interact with spliceosome components to regulate alternative exon expression in human cell lines (50). Finally, motif enrichment analysis in the flanking regions of developmental gain/loss exons identified motifs that were over-represented in the alternatively spliced exons among different tissues, including PTBP1 in brain-related tissues, further indicating the potential link between hPTM and splicing motifs in regulation of alternative spliced exon (Supplemental Figure S72).

In recent years, deep learning models also have been applied in identifying epigenetic factors associated with alternative splicing and gene expression (51,52). Deep learning methods are especially useful with large numbers of features and examples, and techniques that use perturbation or backpropagation (backtracking) (53) can aid in model interpretation. Perturbation-based approaches may better capture the space of inputs that can change an output but are computationally expensive, while backpropagation-based methods are efficient but potentially more limited in their ability to define a full set of features related to an output (53). In short, these methods are powerful predictors but may not be the most suitable methods when the goal is to understand why an input is linked with an output. Here, we had relatively few features so we studied the link between hPTM and splicing with, logistic regression and iterative random forest models, which aligned with our scientific goals. Future studies, particularly those focused primarily on building predictive models from raw sequence-level features, could benefit from deep learning.

Although our model identified potential links between hPTMs and exon splicing, it still has certain limitations. Firstly, without the input information, the ChIP read count could be influenced not only by hPTM enrichment in ChIP-seq data, but by the other factors such as GC context and chromatin accessibility of the region. However, there other studies have pointed out that correcting the effect using ChIP-seq input and nucleosome occupancy has little influence for the original result and major findings still hold (54). In addition, at present, we focus on hPTM enrichment in the exon flanking region, which is that area most likely to be relevant in a direct recruitment model. However, hPTM enrichment further from the exons, in the promoter, from different exons or across the gene, likely contribute to splicing as well. Further analyses will incorporate these distal associations, requiring additional parameters to control for noise and uncertainty introduced into the model. Overall, we have performed a comprehensive analysis to investigate chromatin-mediated alternative splicing events during tissue development. Using computational models, we found that specific histone modifications, H3K36me3 and H3K4me1, have the strongest associations in skipped exon selection among all the tissues and developmental time points examined. We also identified interactions of two or more hPTMs that highly predict AS. For example, the interaction between H3K36me3 and H3K4me1 in the exon flanking region was the top feature in both skipped exon categories. These findings increased the complexity of defining AS regulation, which will inform further experimental studies on the functional relevance of these modifications to alternative splicing.

## Supplementary Material

gkaa248_Supplemental_Files
