# Supplementary material for: Specific histone modifications associate with alternative exon selection during mammalian development
Source: Nucleic Acids Res. 2020 Apr 22;48(9):4709–24. doi: 10.1093/nar/gkaa248 (PMC7229819; doi:10.1093/nar/gkaa248)
Supplement: gkaa248_Supplemental_Files [file gkaa248_supplemental_files.zip › all.supplemental.figs.final.pdf]

Supplemental table 1: number of alternative events identified in different tissues among different time points

Supplemental table 2: Representative GO function for genes involved in alternative splicing in all tissues

Supplemental table 3: Performance of random forest model after and before adding features from constitutive exons for developmental gain/loss and isoform selected high/low exons.

Supplemental table 4: Correlation between hPTMs and skipped exons during tissue development for sets of target genes

Supplemental table 5: Global correlation between hPTMs and psi value for alternative spliced exons during tissue development

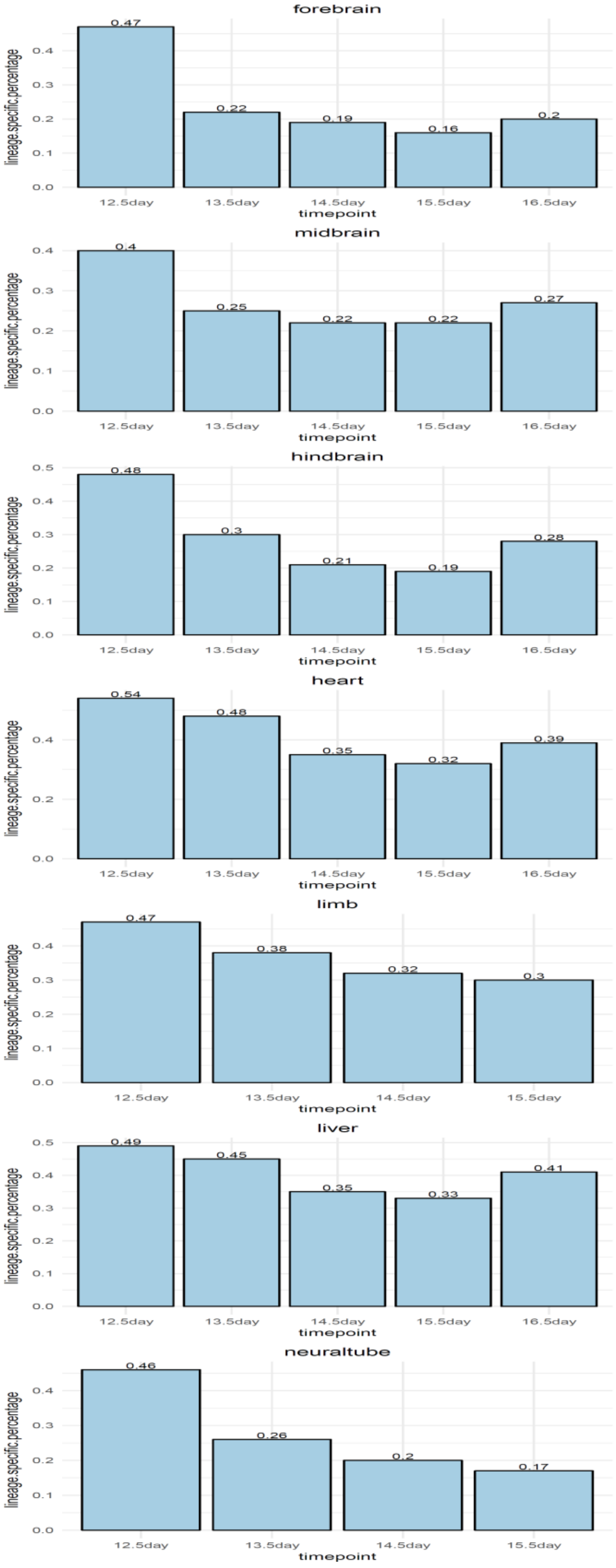

Supplemental figure 1: Percentage of developmentally associated alternative splicing events in different timepoints and brain regions

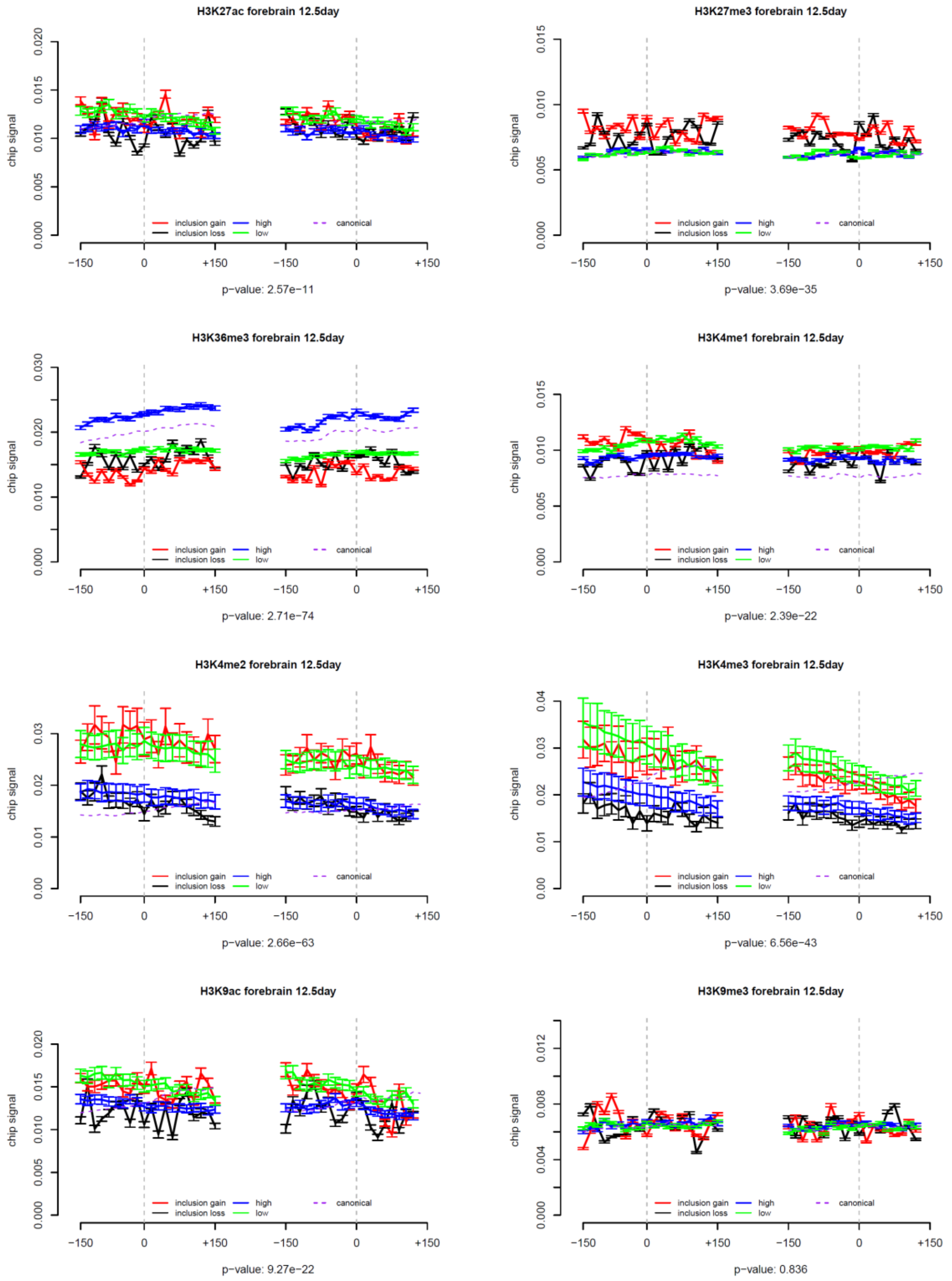

Supplemental figure 2: Representative distribution of mean ChIP-seq signal of 6 types of hPTM, including H3K36me3, H3K4me1, H3K9me3, H3K27ac, H3K4me2 and H3K4me3 on the flanking region (+/- 150bp) of four types of skipped exons in forebrain at E12.5. Dashed grey line shows exon-intron borders.

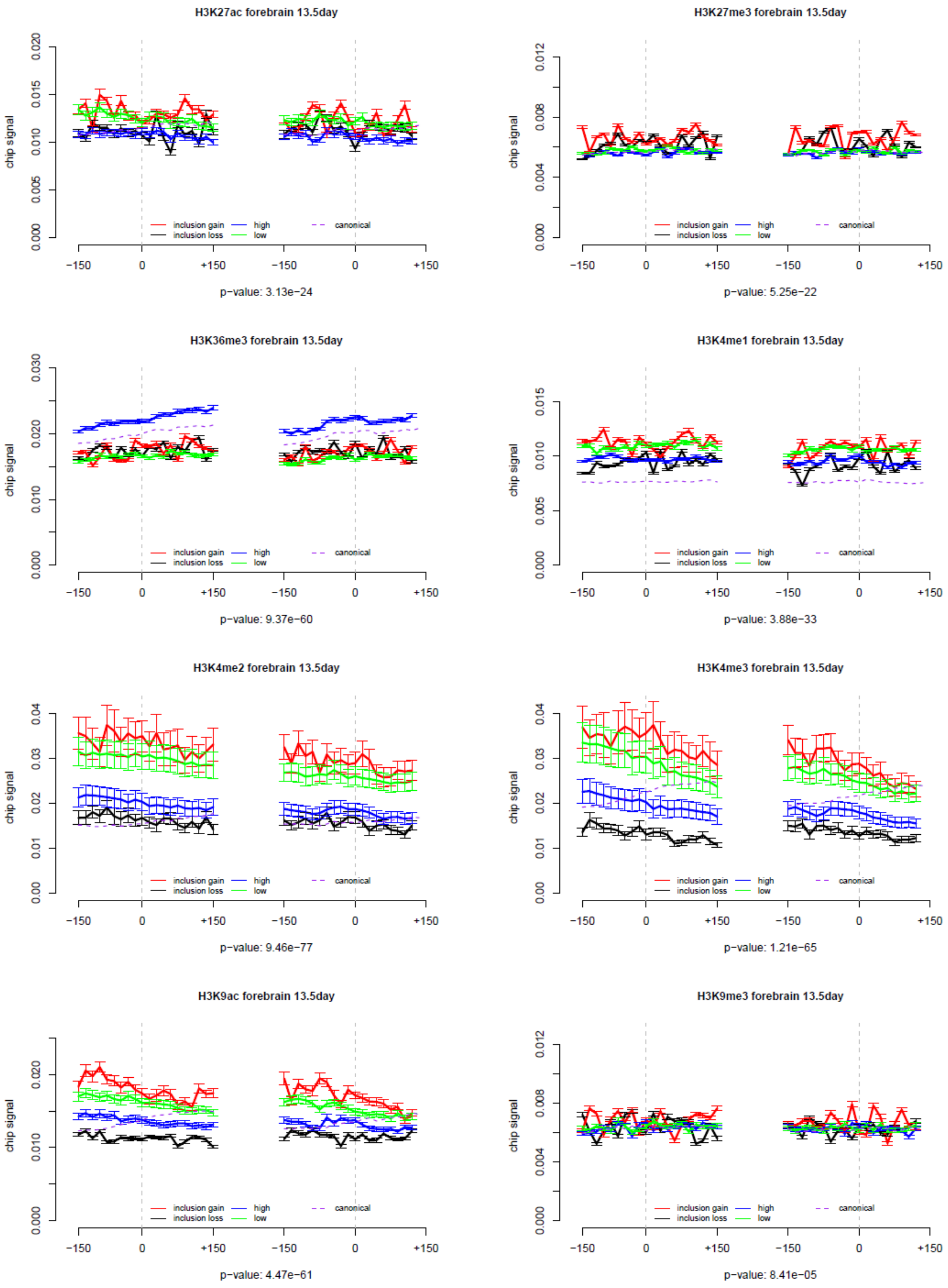

Supplemental figure 3: Representative distribution of mean ChIP-seq signal of 6 types of hPTM, including H3K36me3, H3K4me1, H3K9me3, H3K27ac, H3K4me2 and H3K4me3 on the flanking region (+/- 150bp) of four types of skipped exons in forebrain at E13.5. Dashed grey line shows exon-intron borders.

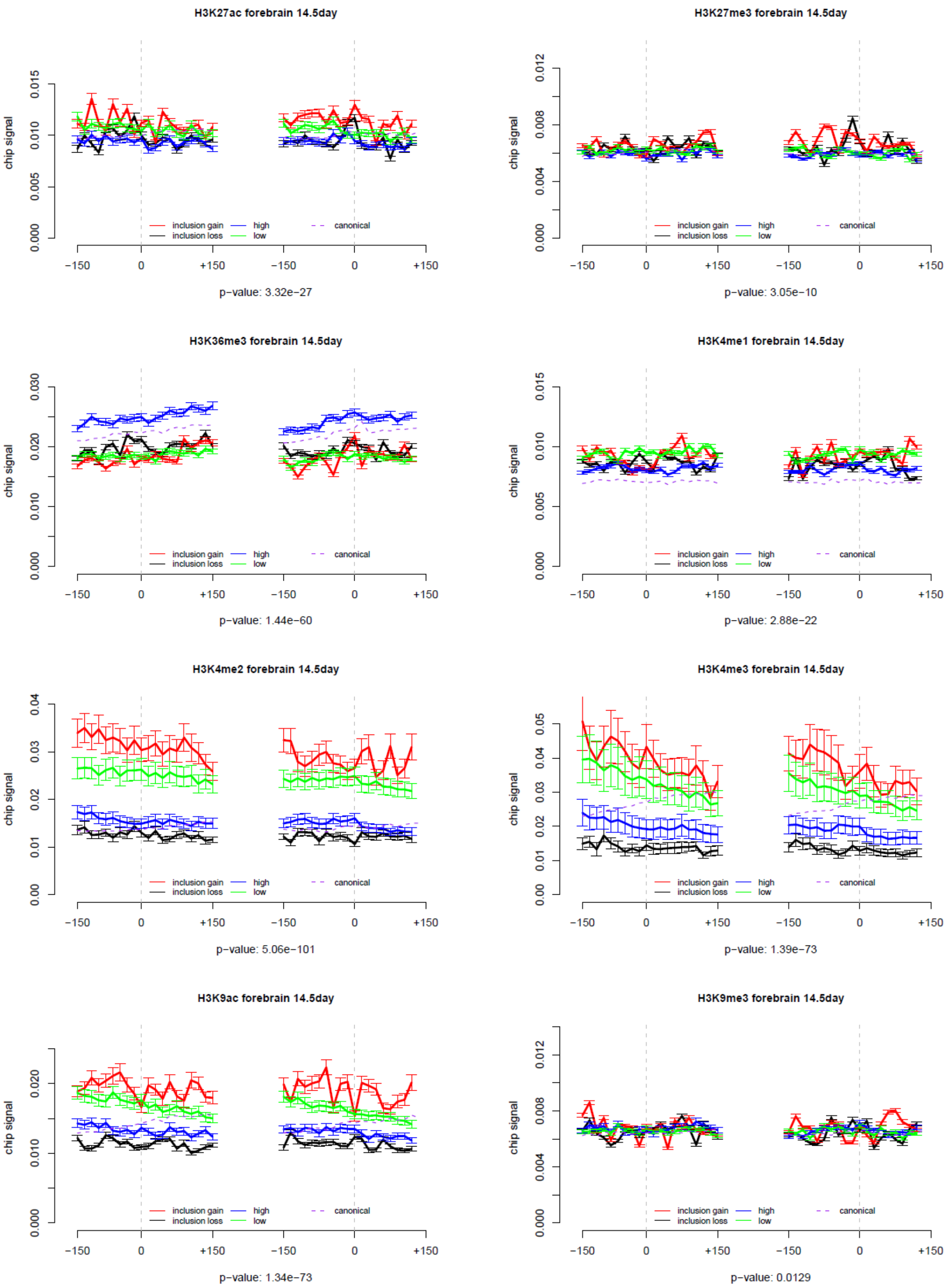

Supplemental figure 4: Representative distribution of mean ChIP-seq signal of 6 types of hPTM, including H3K36me3, H3K4me1, H3K9me3, H3K27ac, H3K4me2 and H3K4me3 on the flanking region (+/- 150bp) of four types of skipped exons in forebrain at E14.5. Dashed grey line shows exon-intron borders.

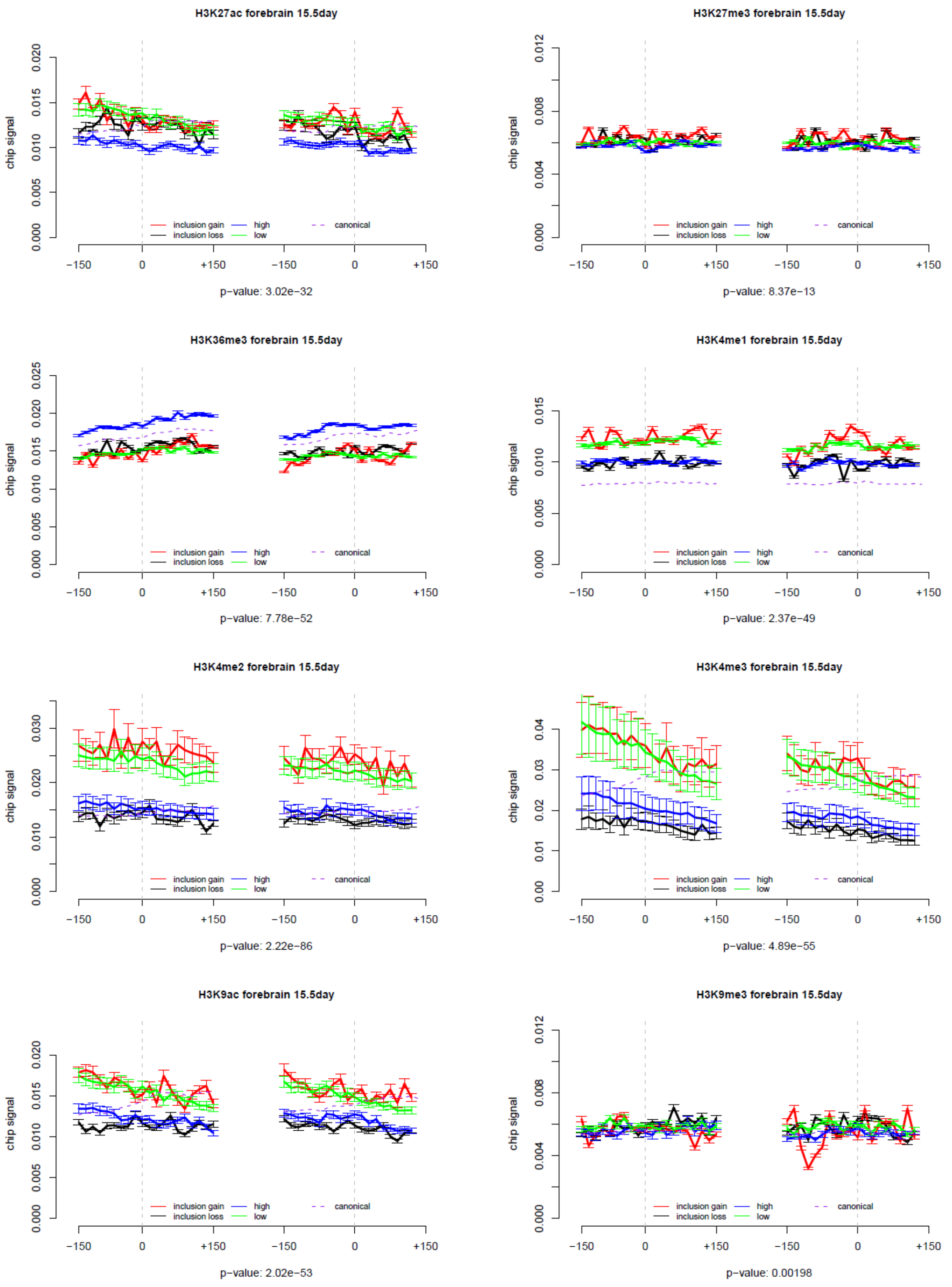

Supplemental figure 5: Representative distribution of mean ChIP-seq signal of 6 types of hPTM, including H3K36me3, H3K4me1, H3K9me3, H3K27ac, H3K4me2 and H3K4me3 on the flanking region (+/- 150bp) of four types of skipped exons in forebrain at E15.5. Dashed grey line shows exon-intron borders.

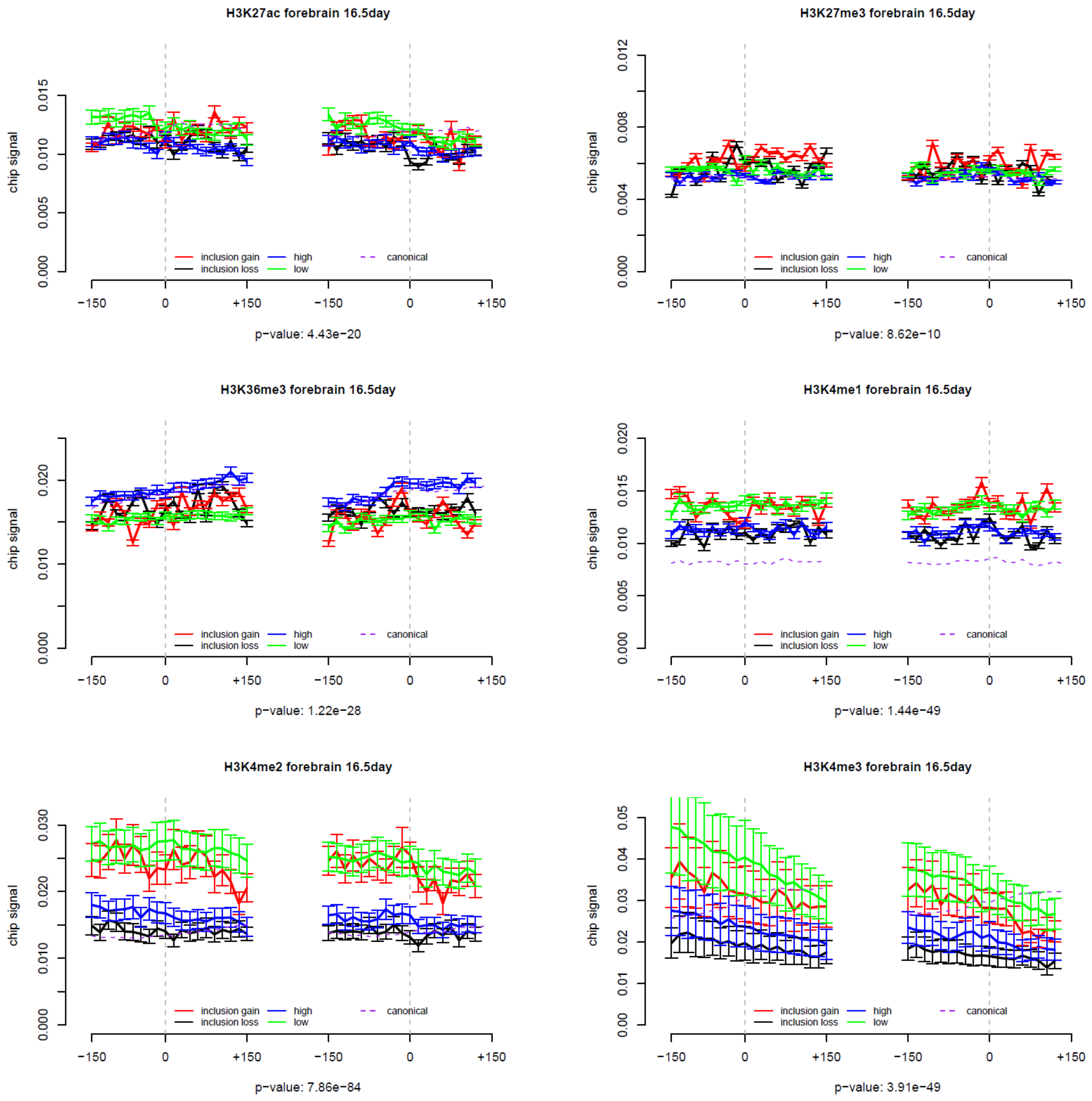

H3K9ac forebrain 16.5day

H3K9me3 forebrain 16.5day

Supplemental figure 6: Representative distribution of mean ChIP-seq signal of 6 types of hPTM, including H3K36me3, H3K4me1, H3K9me3, H3K27ac, H3K4me2 and H3K4me3 on the flanking region (+/- 150bp) of four types of skipped exons in forebrain at E16.5. Dashed grey line shows exon-intron borders.

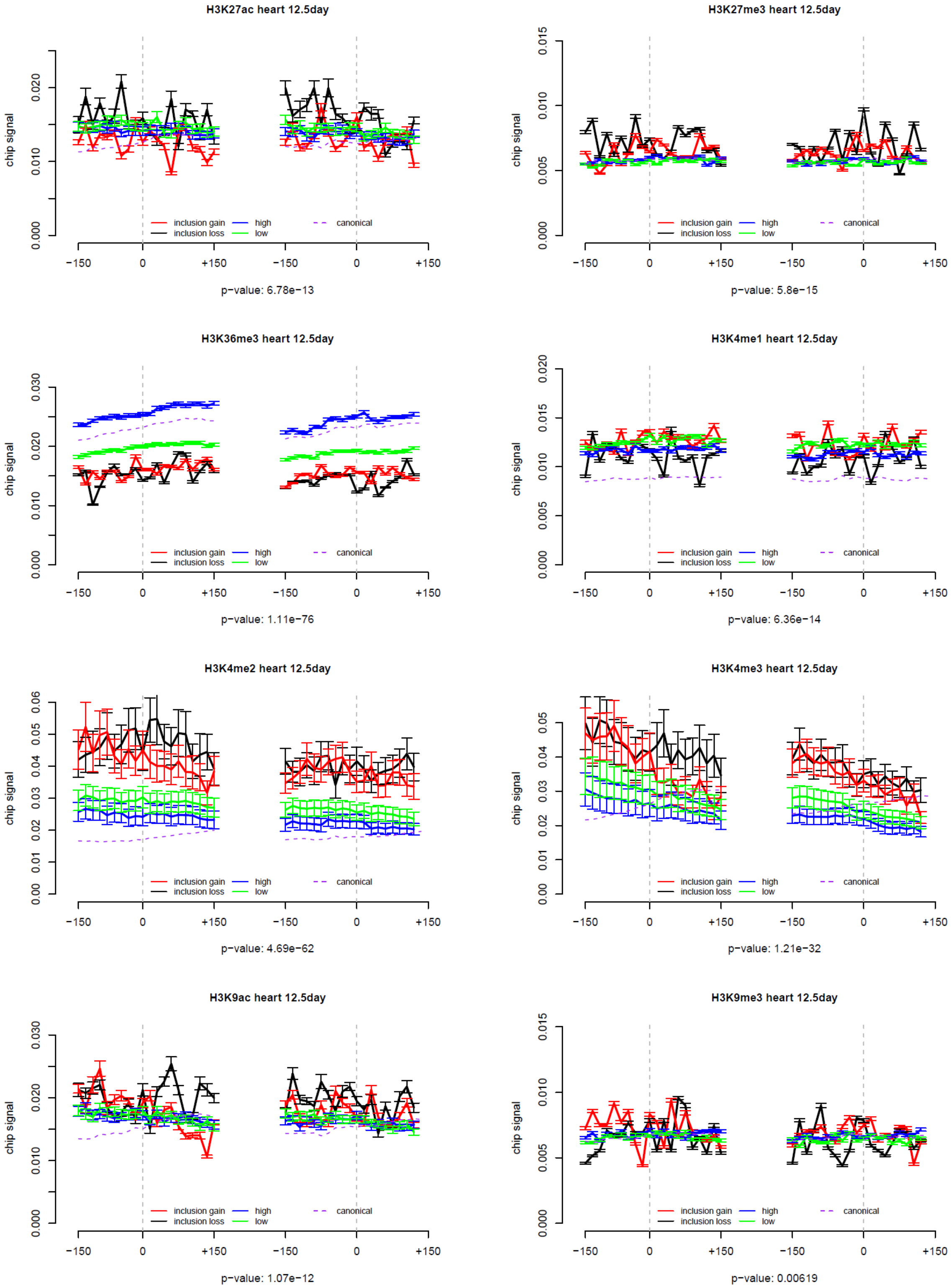

Supplemental figure 7: Representative distribution of mean ChIP-seq signal of 6 types of hPTM, including H3K36me3, H3K4me1, H3K9me3, H3K27ac, H3K4me2 and H3K4me3 on the flanking region (+/- 150bp) of four types of skipped exons in heart at E12.5. Dashed grey line shows exon-intron borders.

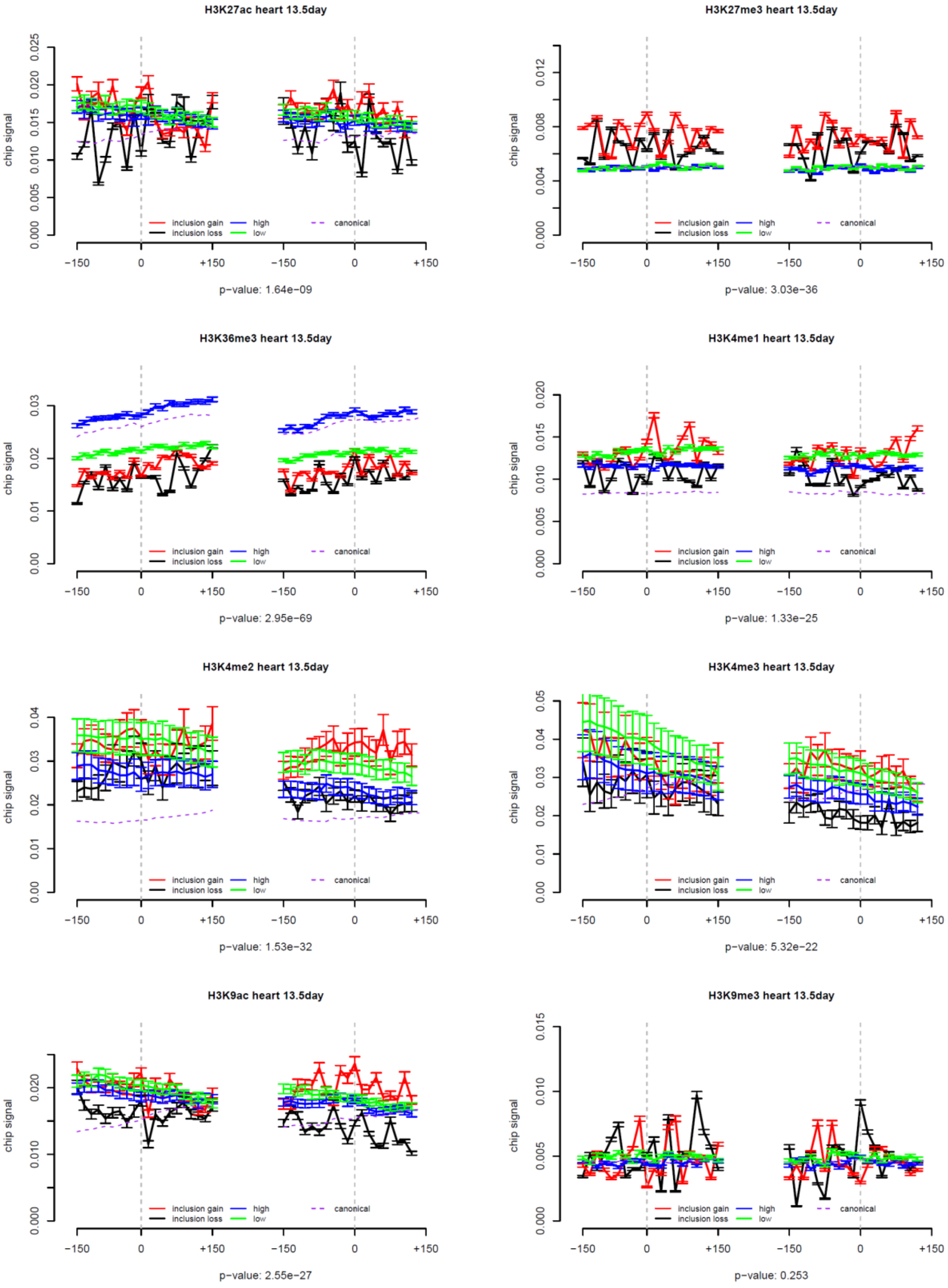

Supplemental figure 8: Representative distribution of mean ChIP-seq signal of 6 types of hPTM, including H3K36me3, H3K4me1, H3K9me3, H3K27ac, H3K4me2 and H3K4me3 on the flanking region (+/- 150bp) of four types of skipped exons in heart at E13.5. Dashed grey line shows exon-intron borders.

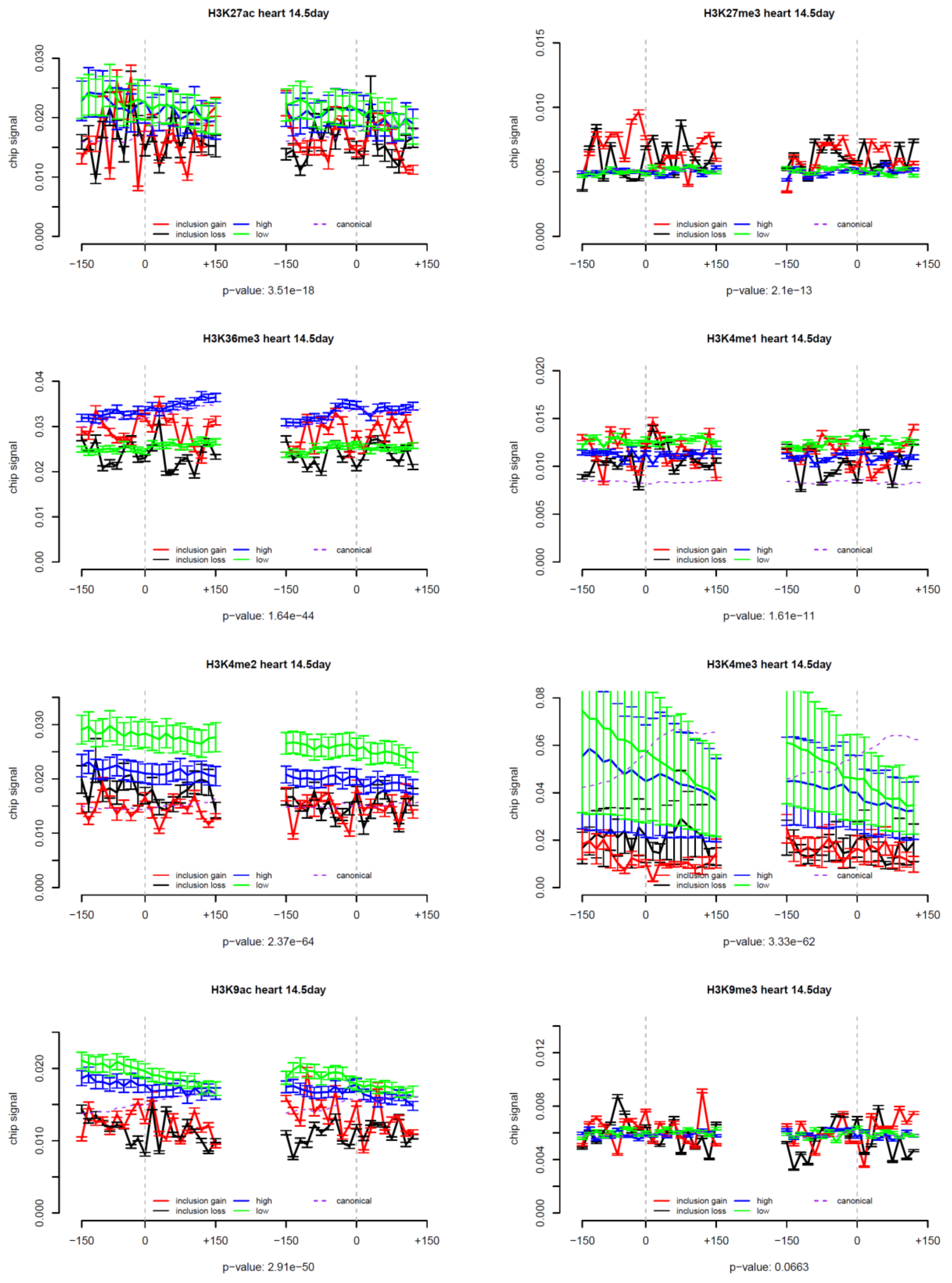

Supplemental figure 9: Representative distribution of mean ChIP-seq signal of 6 types of hPTM, including H3K36me3, H3K4me1, H3K9me3, H3K27ac, H3K4me2 and H3K4me3 on the flanking region (+/- 150bp) of four types of skipped exons in heart at E14.5. Dashed grey line shows exon-intron borders.

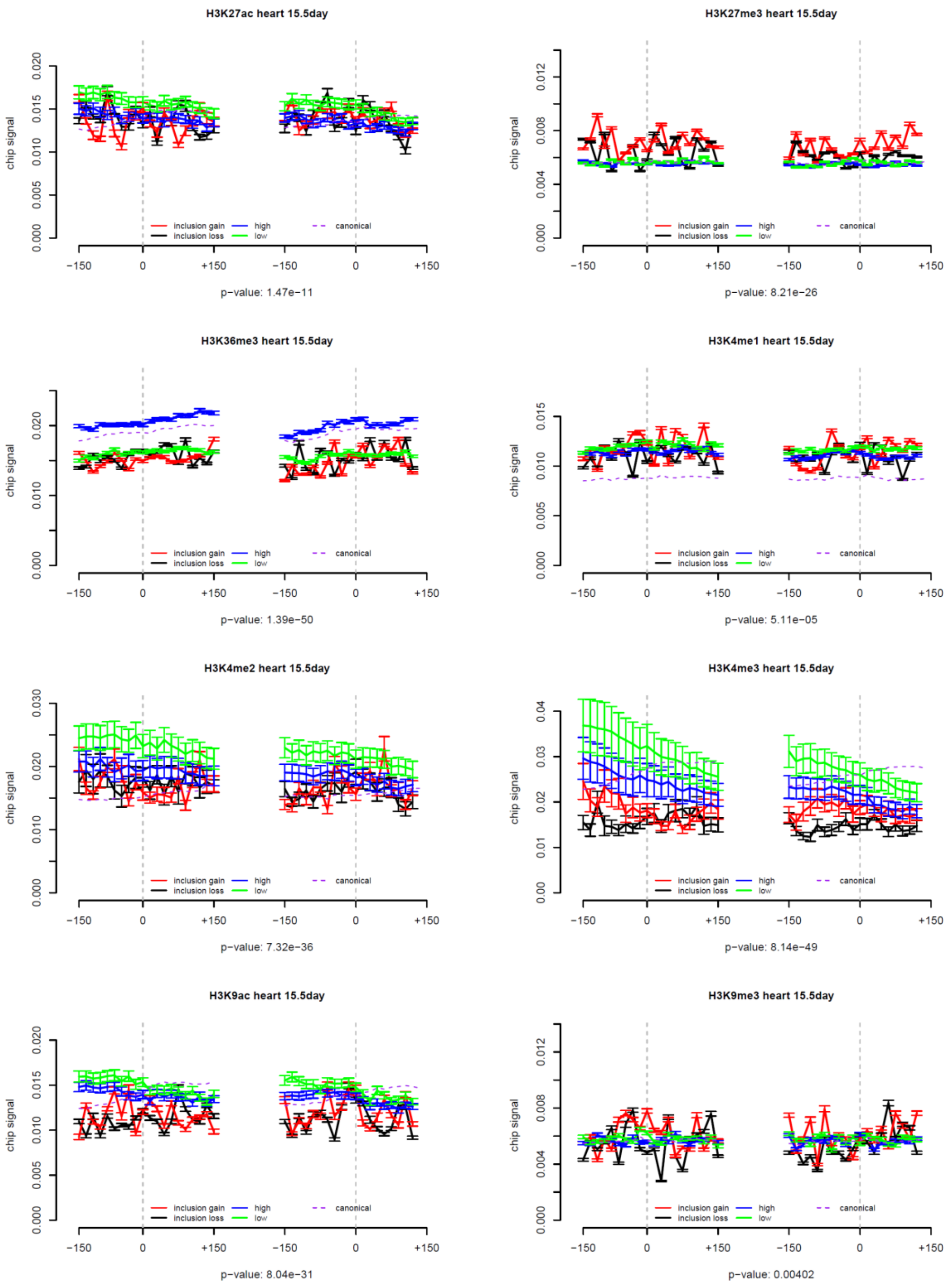

Supplemental figure 10: Representative distribution of mean ChIP-seq signal of 6 types of hPTM, including H3K36me3, H3K4me1, H3K9me3, H3K27ac, H3K4me2 and H3K4me3 on the flanking region (+/- 150bp) of four types of skipped exons in heart at E15.5. Dashed grey line shows exon-intron borders.

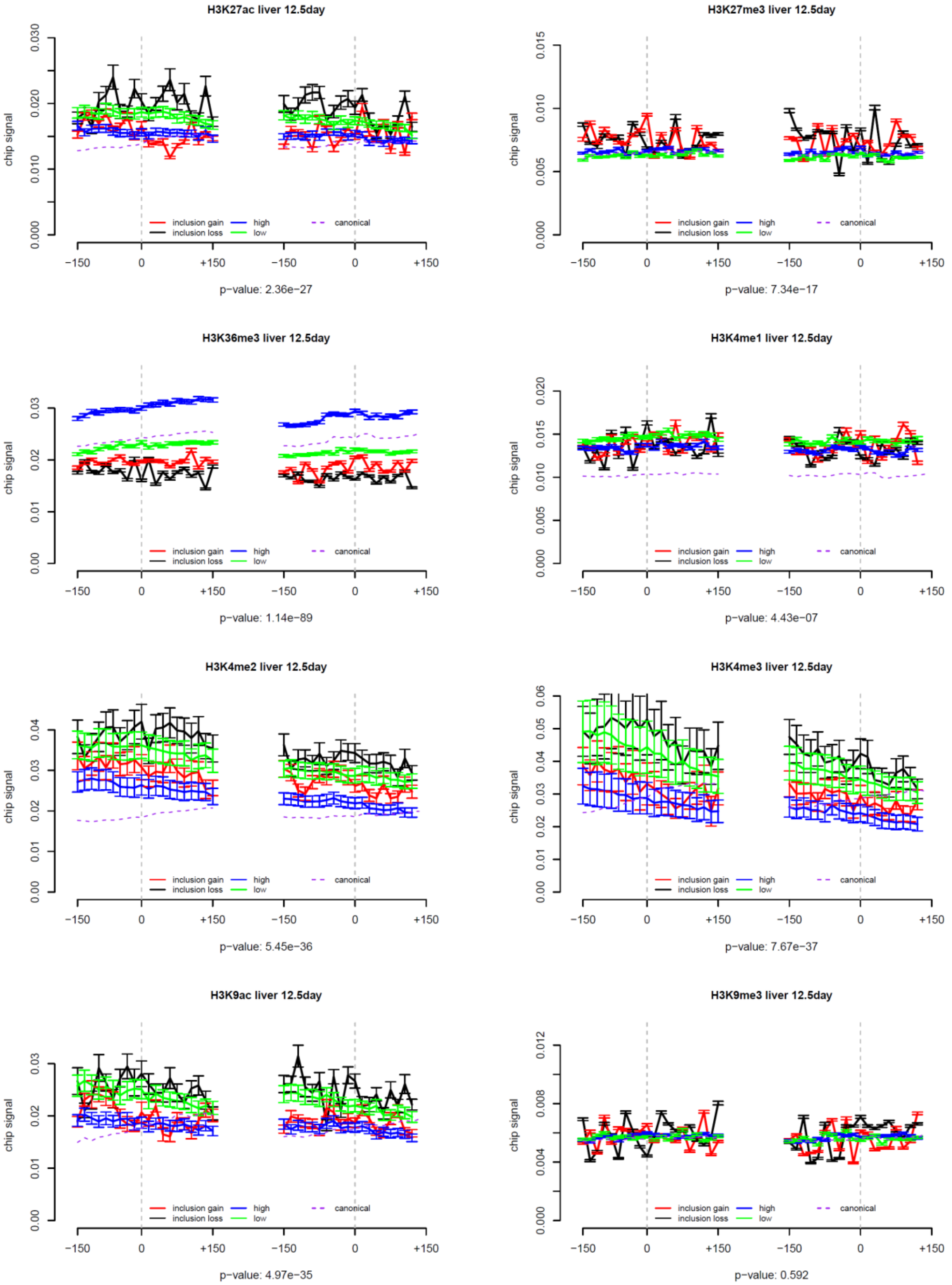

Supplemental figure 11: Representative distribution of mean ChIP-seq signal of 6 types of hPTM, including H3K36me3, H3K4me1, H3K9me3, H3K27ac, H3K4me2 and H3K4me3 on the flanking region (+/- 150bp) of four types of skipped exons in liver at E12.5. Dashed grey line shows exon-intron borders.

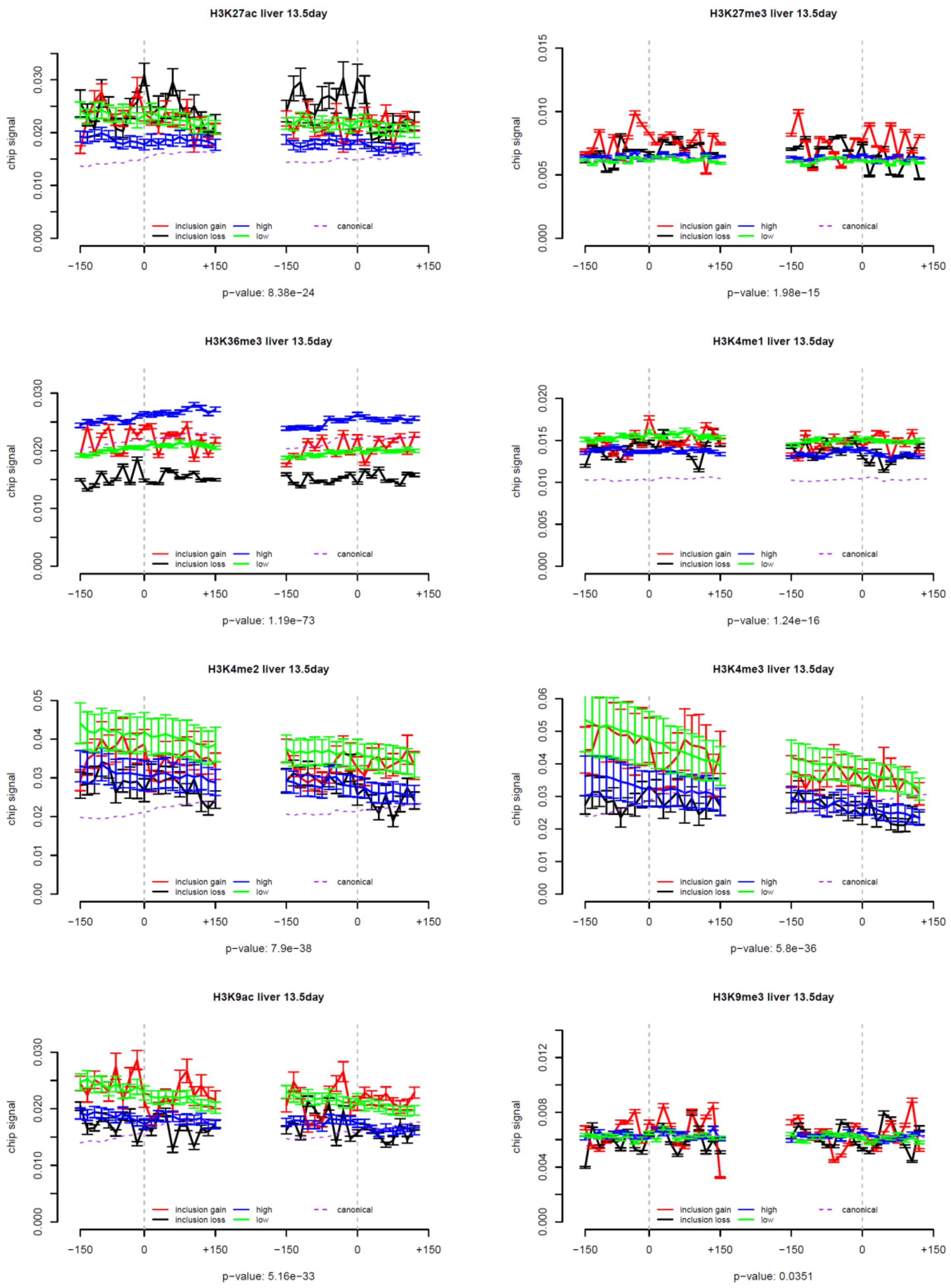

Supplemental figure 12: Representative distribution of mean ChIP-seq signal of 6 types of hPTM, including H3K36me3, H3K4me1, H3K9me3, H3K27ac, H3K4me2 and H3K4me3 on the flanking region (+/- 150bp) of four types of skipped exons in liver at E13.5. Dashed grey line shows exon-intron borders.

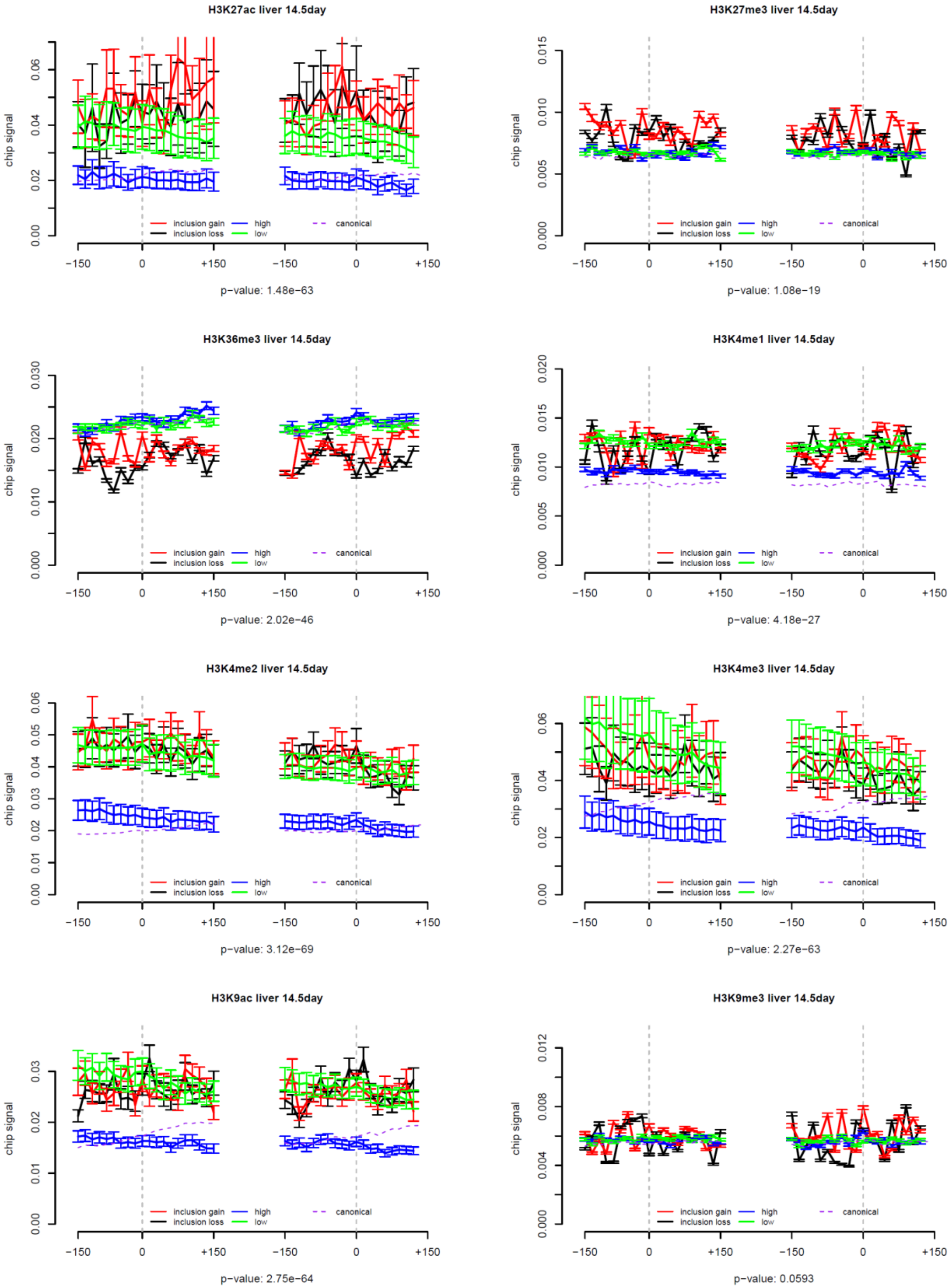

Supplemental figure 13: Representative distribution of mean ChIP-seq signal of 6 types of hPTM, including H3K36me3, H3K4me1, H3K9me3, H3K27ac, H3K4me2 and H3K4me3 on the flanking region (+/- 150bp) of four types of skipped exons in liver at E14.5. Dashed grey line shows exon-intron borders.

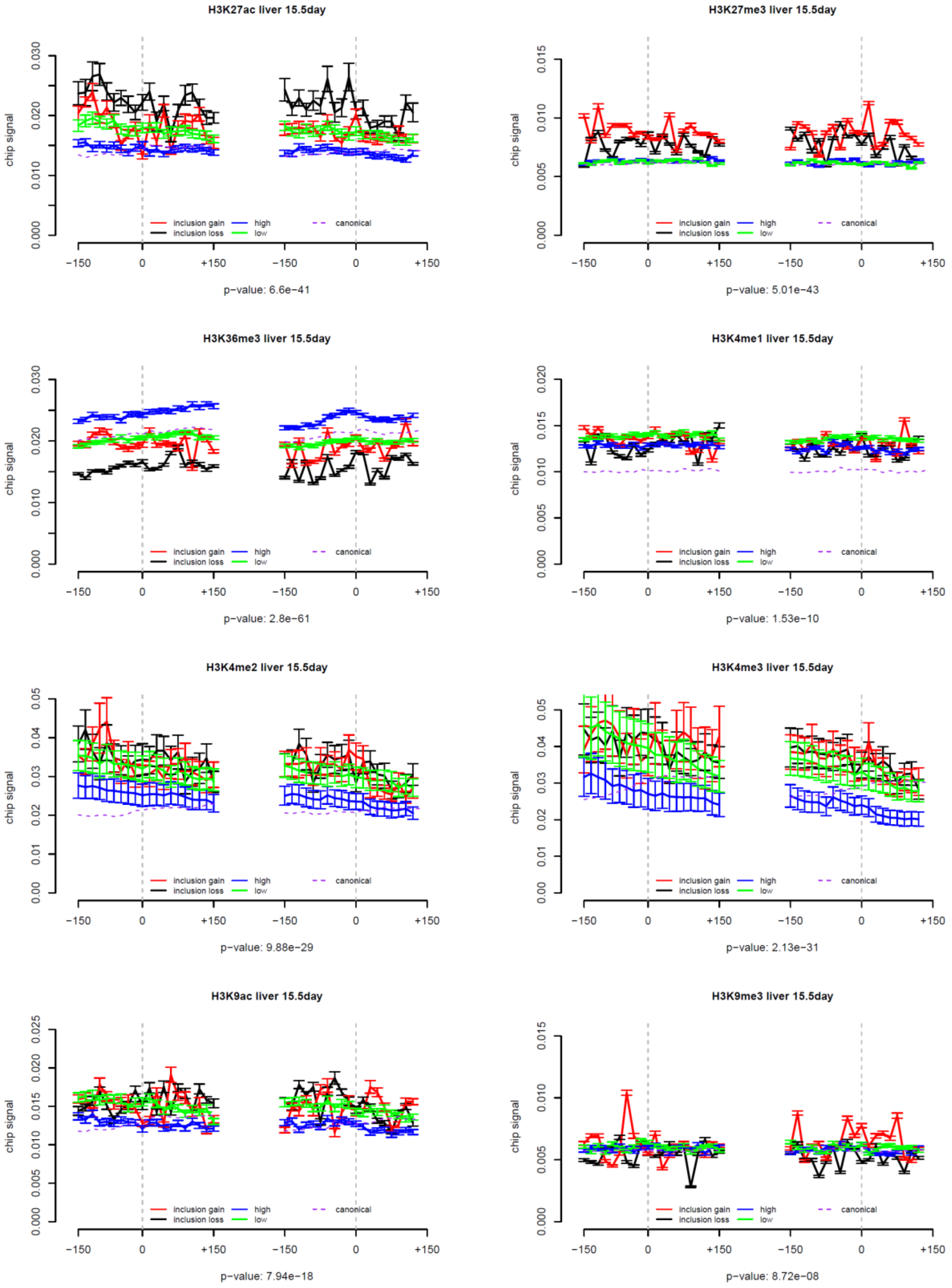

Supplemental figure 14: Representative distribution of mean ChIP-seq signal of 6 types of hPTM, including H3K36me3, H3K4me1, H3K9me3, H3K27ac, H3K4me2 and H3K4me3 on the flanking region (+/- 150bp) of four types of skipped exons in liver at E15.5. Dashed grey line shows exon-intron borders.

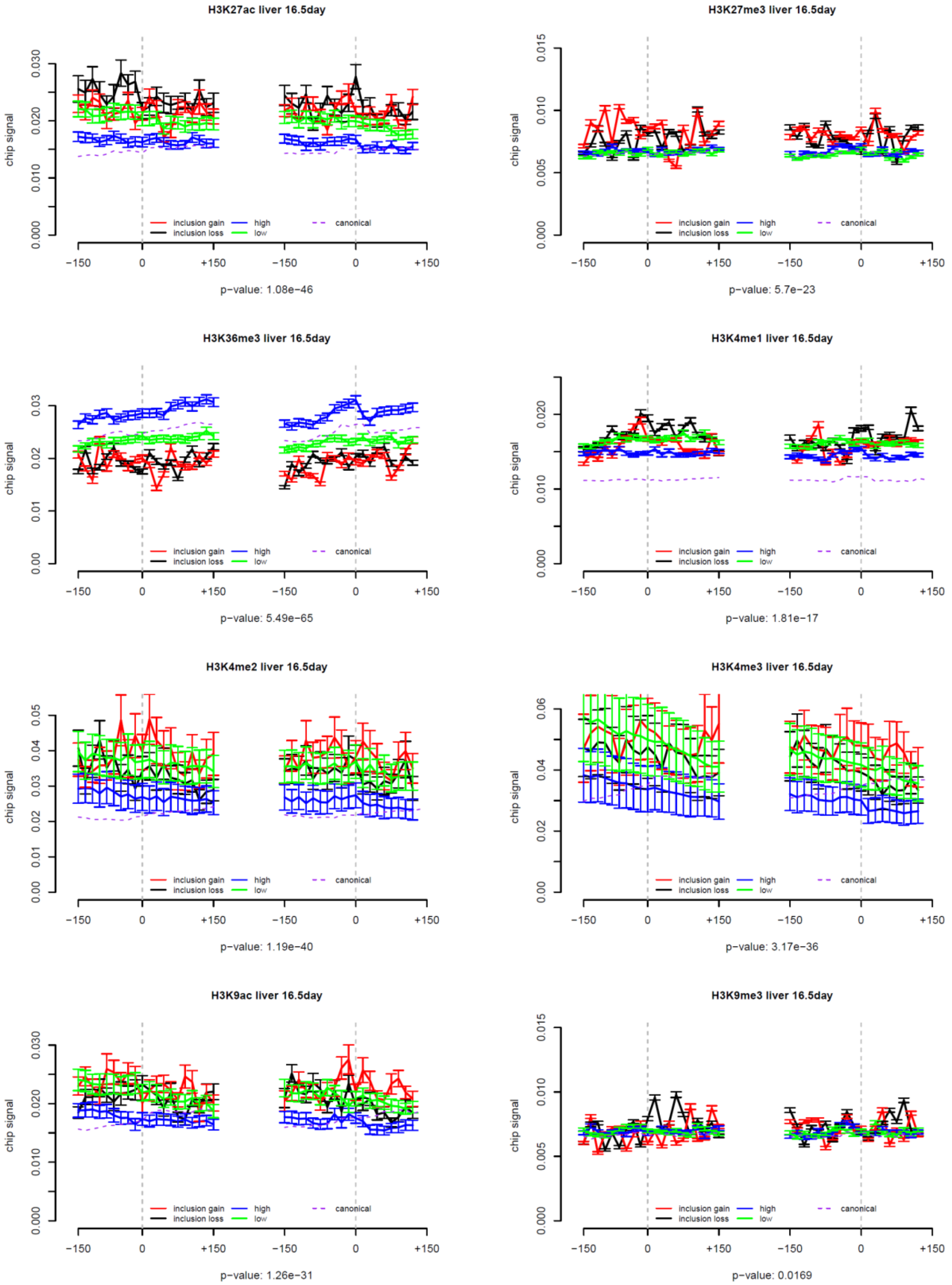

Supplemental figure 15: Representative distribution of mean ChIP-seq signal of 6 types of hPTM, including H3K36me3, H3K4me1, H3K9me3, H3K27ac, H3K4me2 and H3K4me3 on the flanking region (+/- 150bp) of four types of skipped exons in liver at E16.5. Dashed grey line shows exon-intron borders.

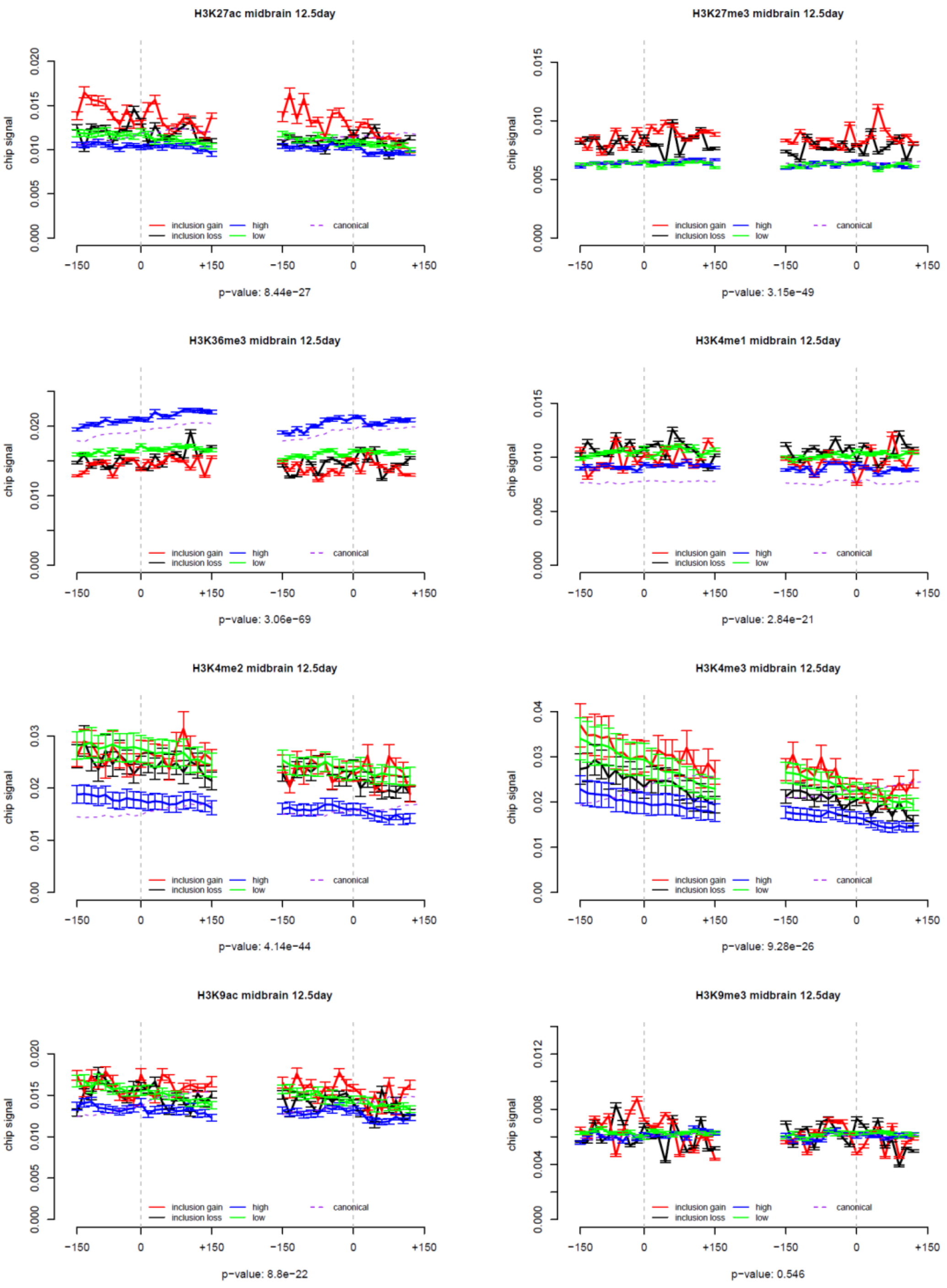

Supplemental figure 16: Representative distribution of mean ChIP-seq signal of 6 types of hPTM, including H3K36me3, H3K4me1, H3K9me3, H3K27ac, H3K4me2 and H3K4me3 on the flanking region (+/- 150bp) of four types of skipped exons in midbrain at E12.5. Dashed grey line shows exon-intron borders.

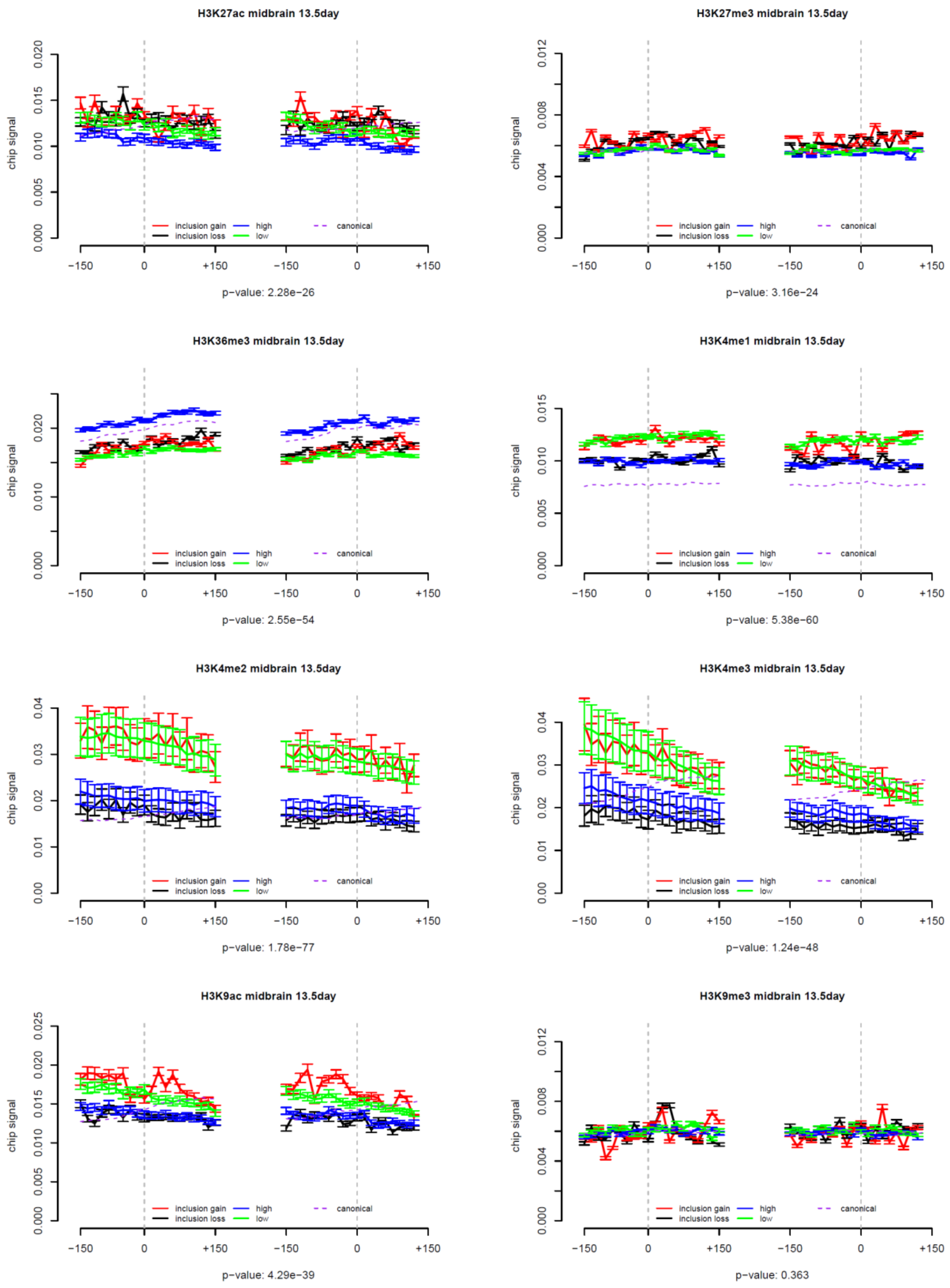

Supplemental figure 17: Representative distribution of mean ChIP-seq signal of 6 types of hPTM, including H3K36me3, H3K4me1, H3K9me3, H3K27ac, H3K4me2 and H3K4me3 on the flanking region (+/- 150bp) of four types of skipped exons in midbrain at 13.5day. Dashed grey line shows exon-intron borders.

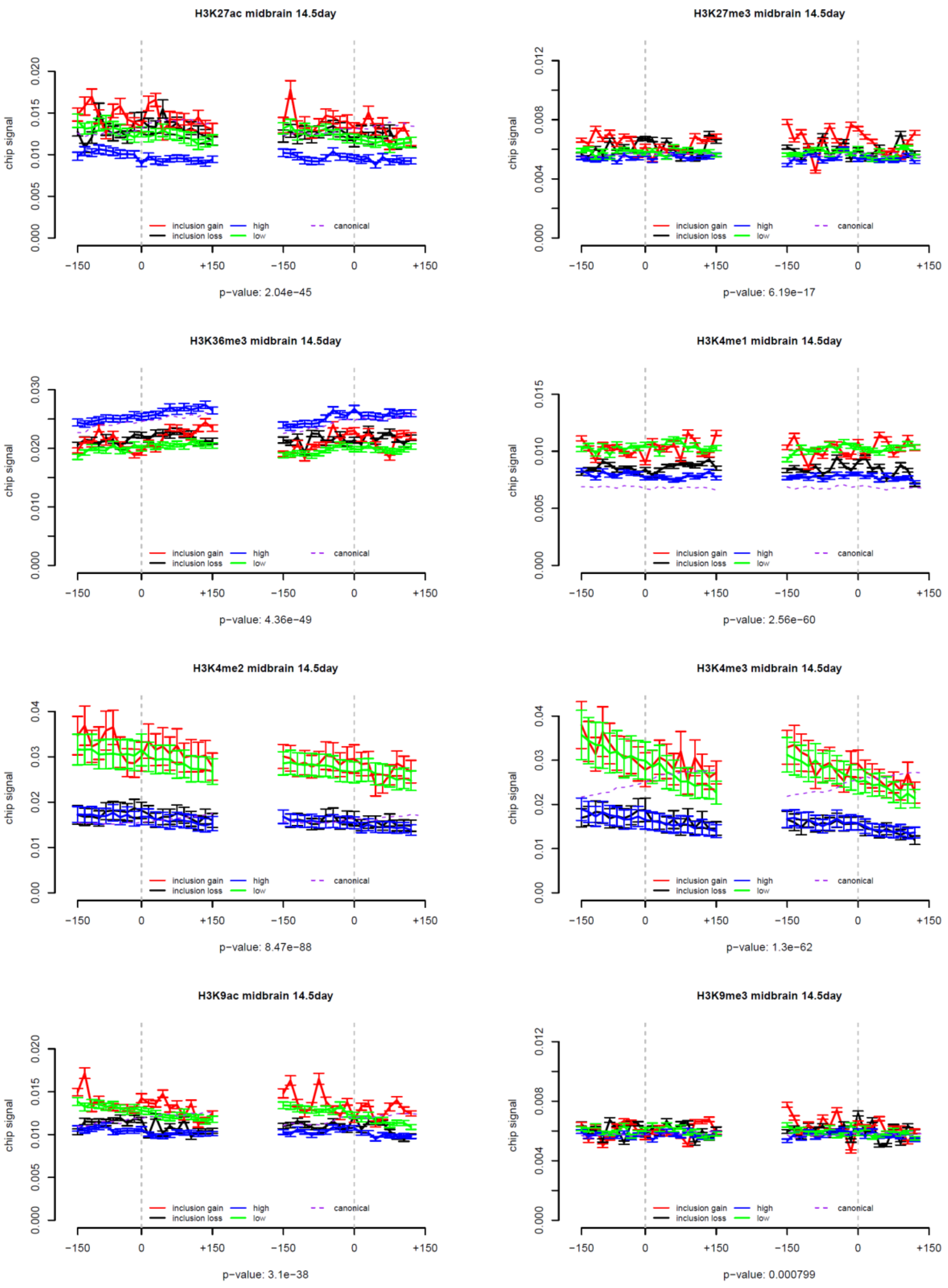

Supplemental figure 18: Representative distribution of mean ChIP-seq signal of 6 types of hPTM, including H3K36me3, H3K4me1, H3K9me3, H3K27ac, H3K4me2 and H3K4me3 on the flanking region (+/- 150bp) of four types of skipped exons in midbrain at E14.5. Dashed grey line shows exon-intron borders.

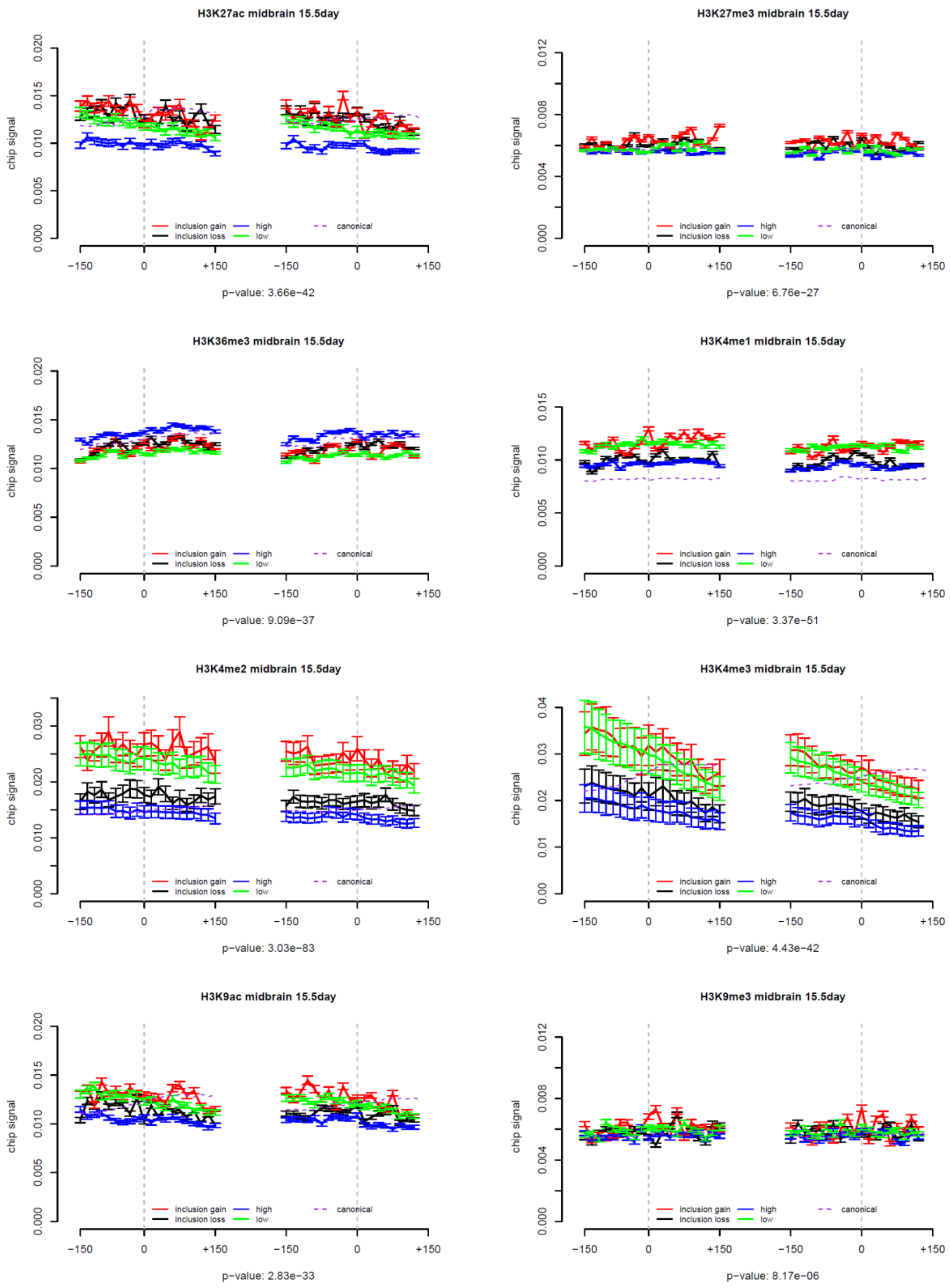

Supplemental figure 19: Representative distribution of mean ChIP-seq signal of 6 types of hPTM, including H3K36me3, H3K4me1, H3K9me3, H3K27ac, H3K4me2 and H3K4me3 on the flanking region (+/- 150bp) of four types of skipped exons in midbrain at E15.5. Dashed grey line shows exon-intron borders.

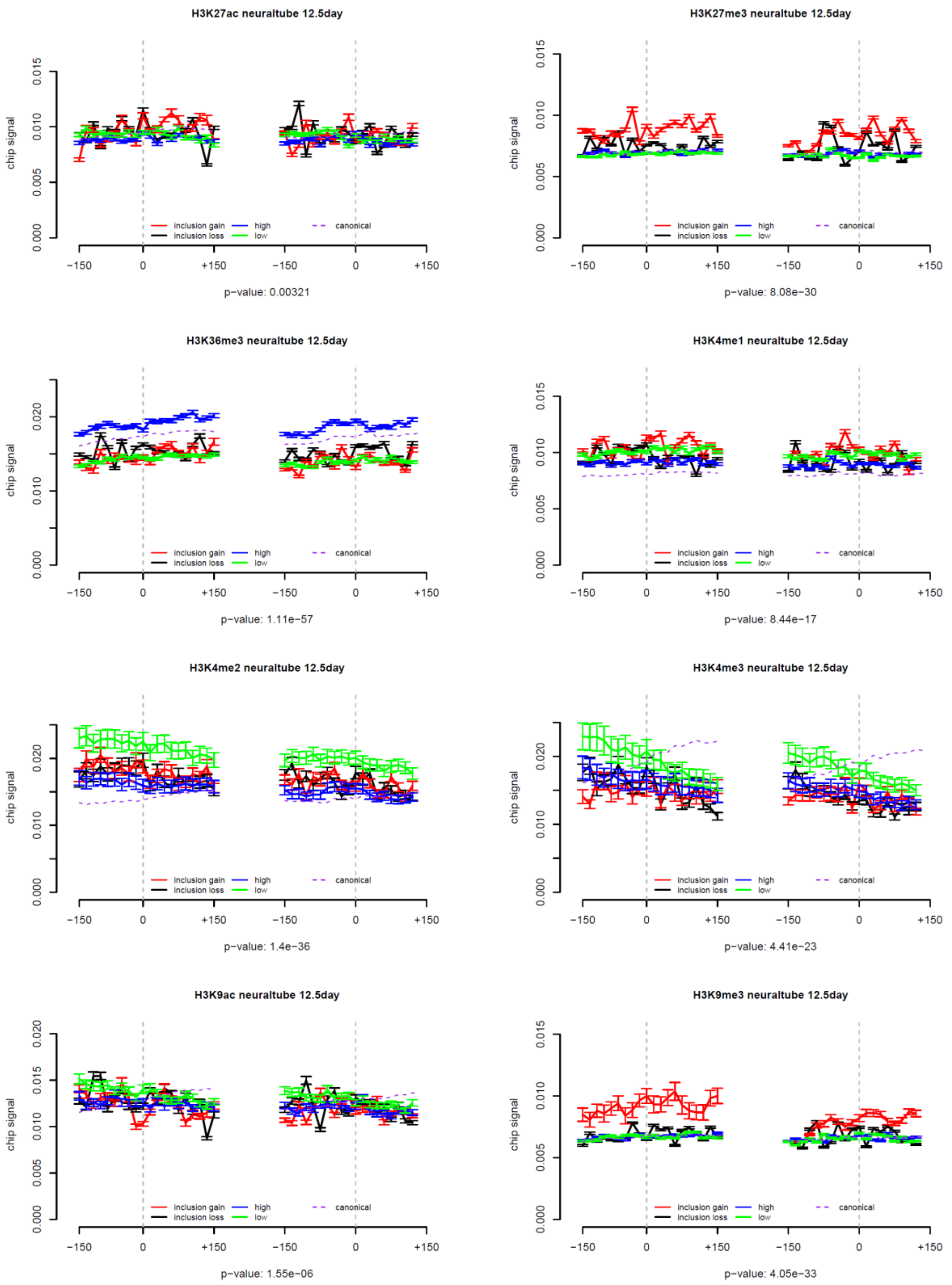

Supplemental figure 20: Representative distribution of mean ChIP-seq signal of 6 types of hPTM, including H3K36me3, H3K4me1, H3K9me3, H3K27ac, H3K4me2 and H3K4me3 on the flanking region (+/- 150bp) of four types of skipped exons in neuraltube at E12.5. Dashed grey line shows exon-intron borders.

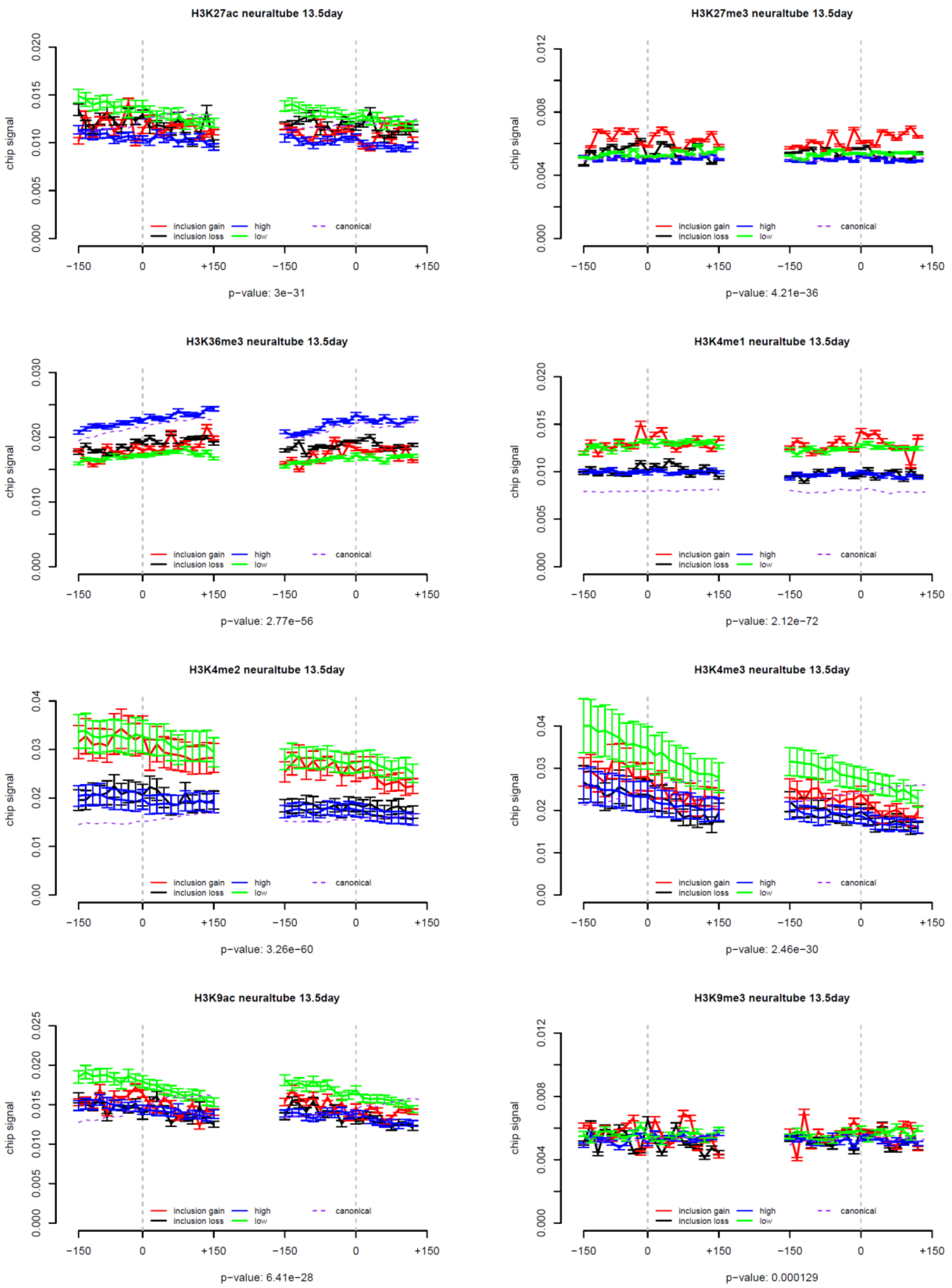

Supplemental figure 21: Representative distribution of mean ChIP-seq signal of 6 types of hPTM, including H3K36me3, H3K4me1, H3K9me3, H3K27ac, H3K4me2 and H3K4me3 on the flanking region (+/- 150bp) of four types of skipped exons in neuraltube at 13.5day. Dashed grey line shows exon-intron borders.

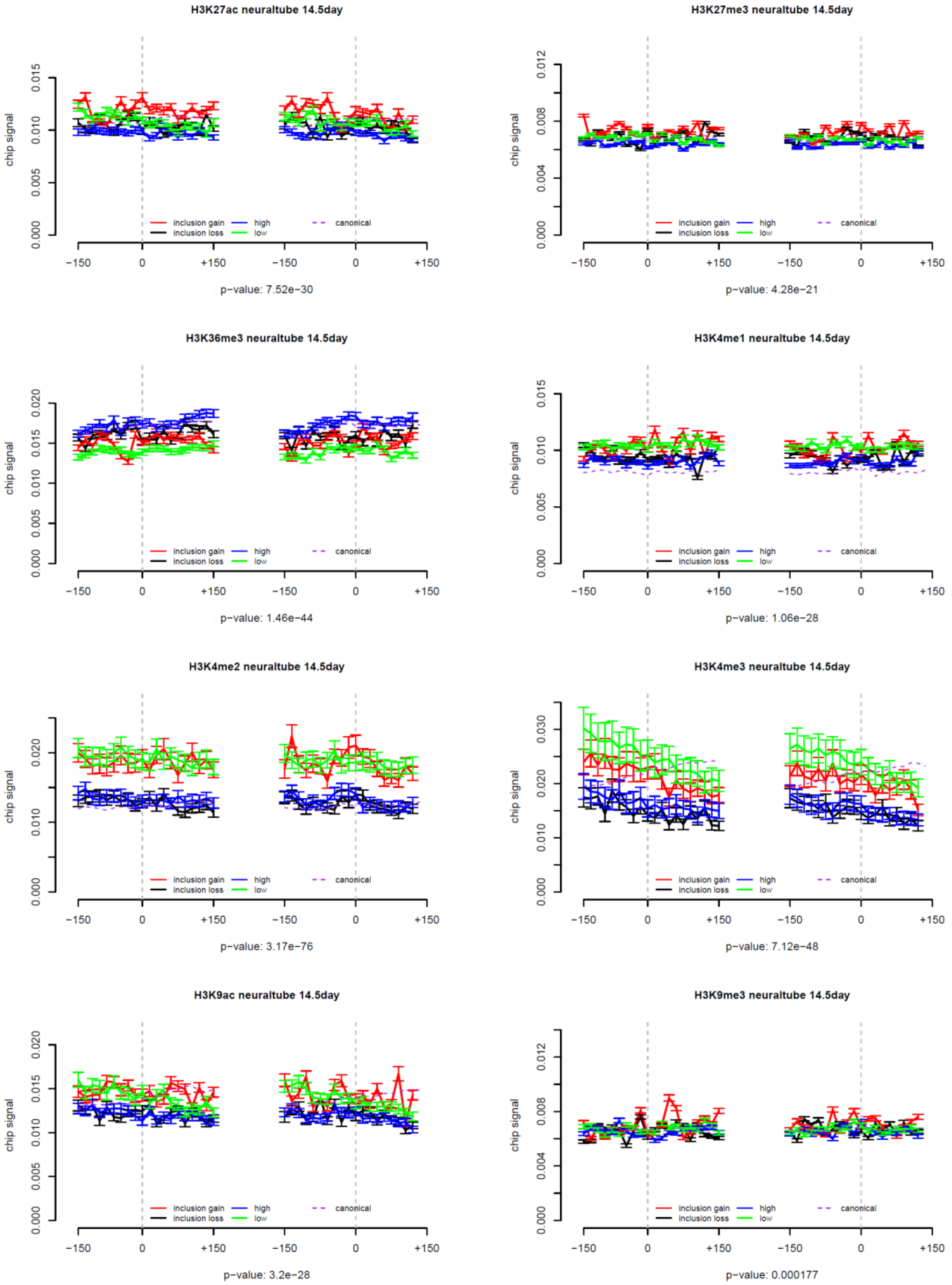

Supplemental figure 22: Representative distribution of mean ChIP-seq signal of 6 types of hPTM, including H3K36me3, H3K4me1, H3K9me3, H3K27ac, H3K4me2 and H3K4me3 on the flanking region (+/- 150bp) of four types of skipped exons in neuraltube at E14.5. Dashed grey line shows exon-intron borders.

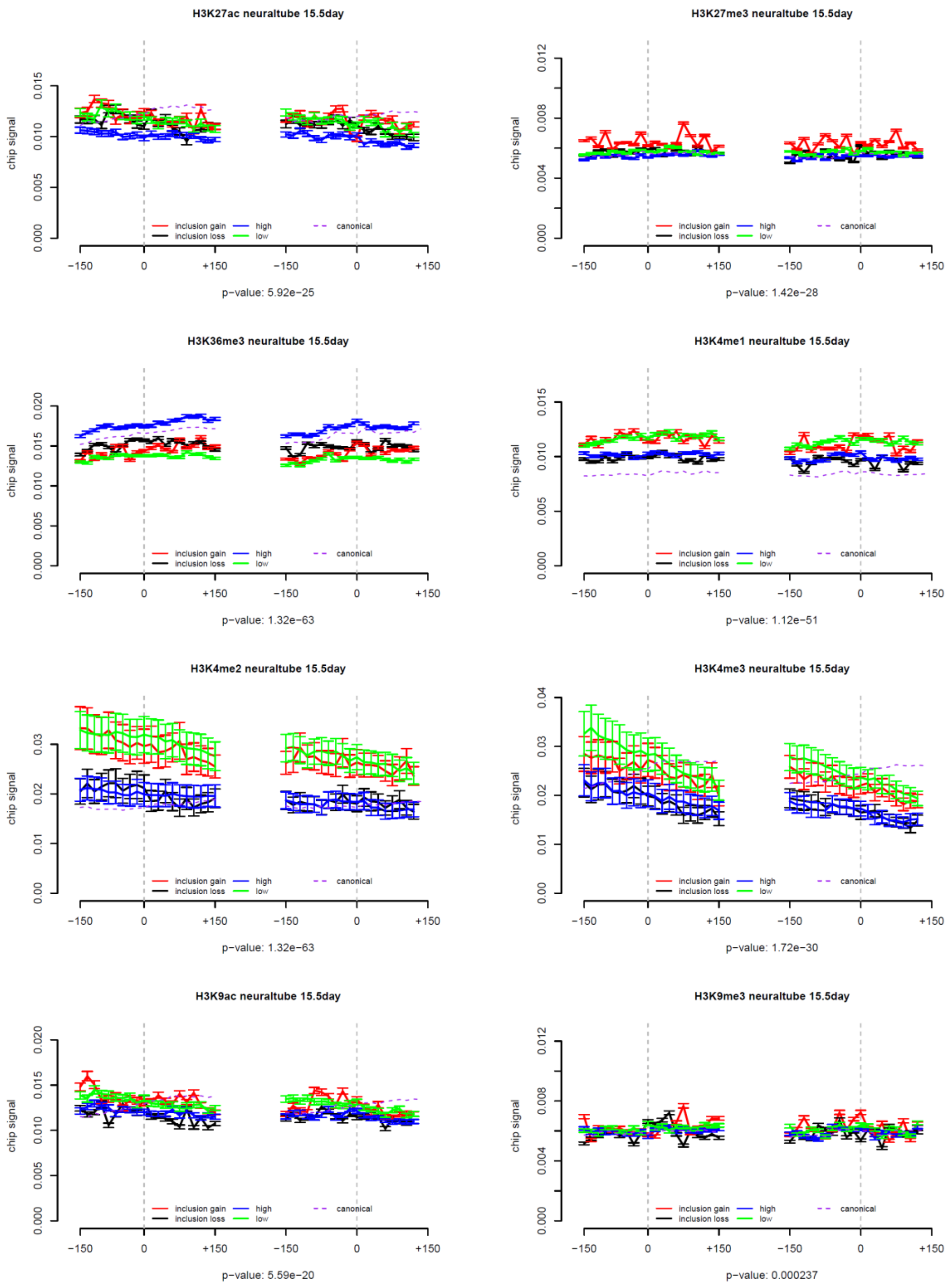

Supplemental figure 23: Representative distribution of mean ChIP-seq signal of 6 types of hPTM, including H3K36me3, H3K4me1, H3K9me3, H3K27ac, H3K4me2 and H3K4me3 on the flanking region (+/- 150bp) of four types of skipped exons in neuraltube at E15.5. Dashed grey line shows exon-intron borders.

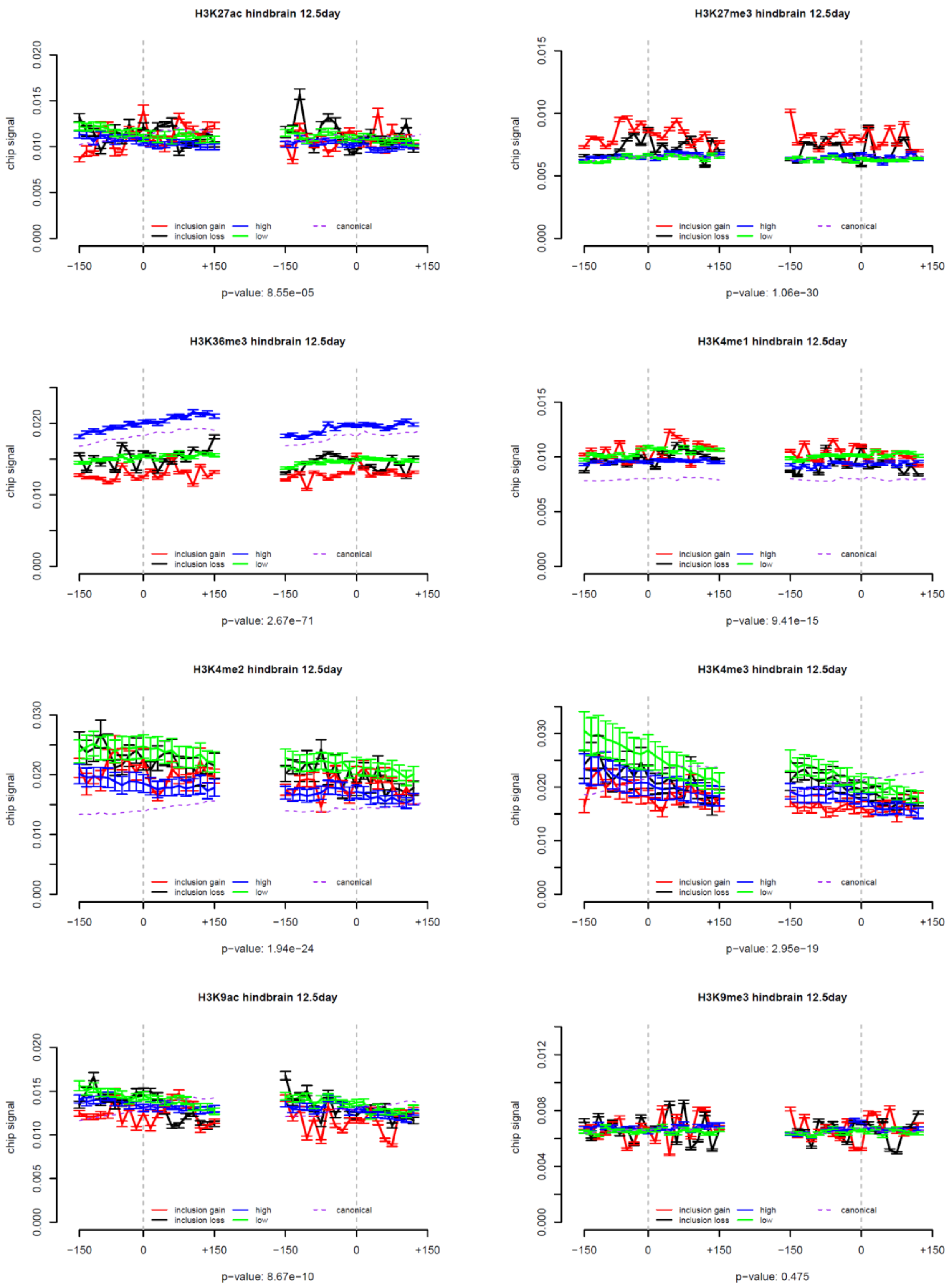

Supplemental figure 24: Representative distribution of mean ChIP-seq signal of 6 types of hPTM, including H3K36me3, H3K4me1, H3K9me3, H3K27ac, H3K4me2 and H3K4me3 on the flanking region (+/- 150bp) of four types of skipped exons in hindbrain at E12.5. Dashed grey line shows exon-intron borders.

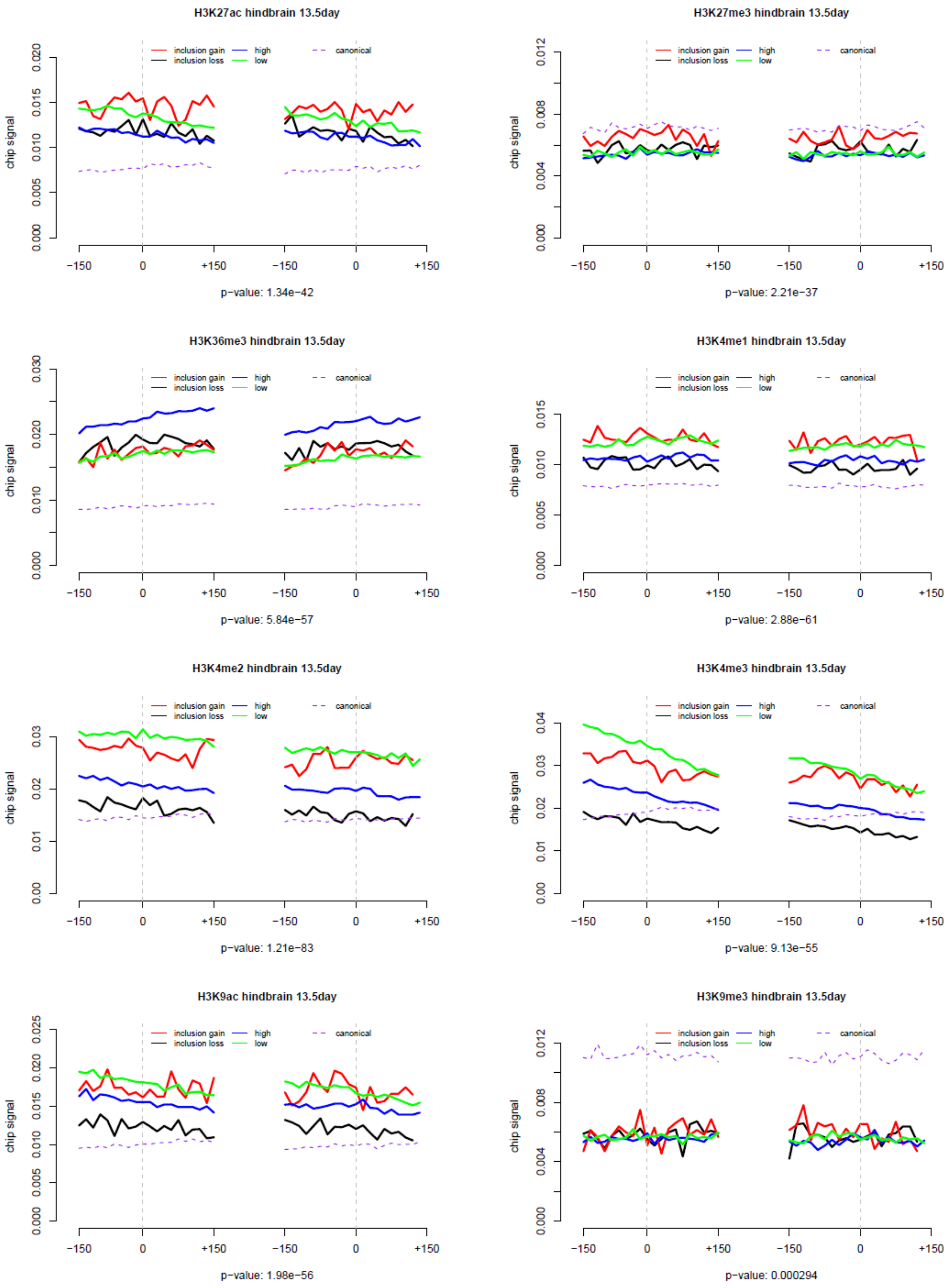

Supplemental figure 25: Representative distribution of mean ChIP-seq signal of 6 types of hPTM, including H3K36me3, H3K4me1, H3K9me3, H3K27ac, H3K4me2 and H3K4me3 on the flanking region (+/- 150bp) of four types of skipped exons in hindbrain at 13.5day. Dashed grey line shows exon-intron borders.

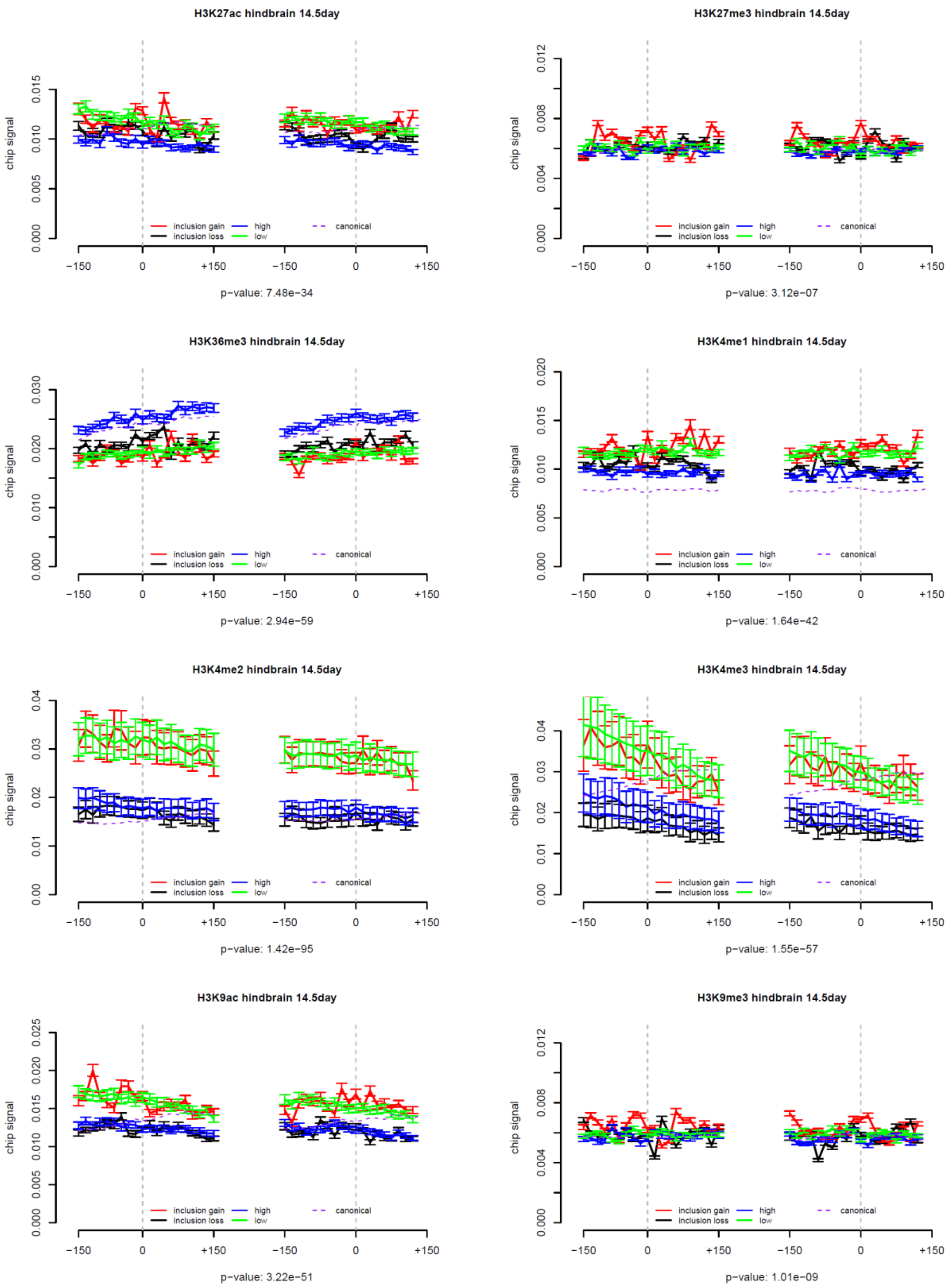

Supplemental figure 26: Representative distribution of mean ChIP-seq signal of 6 types of hPTM, including H3K36me3, H3K4me1, H3K9me3, H3K27ac, H3K4me2 and H3K4me3 on the flanking region (+/- 150bp) of four types of skipped exons in hindbrain at E14.5. Dashed grey line shows exon-intron borders.

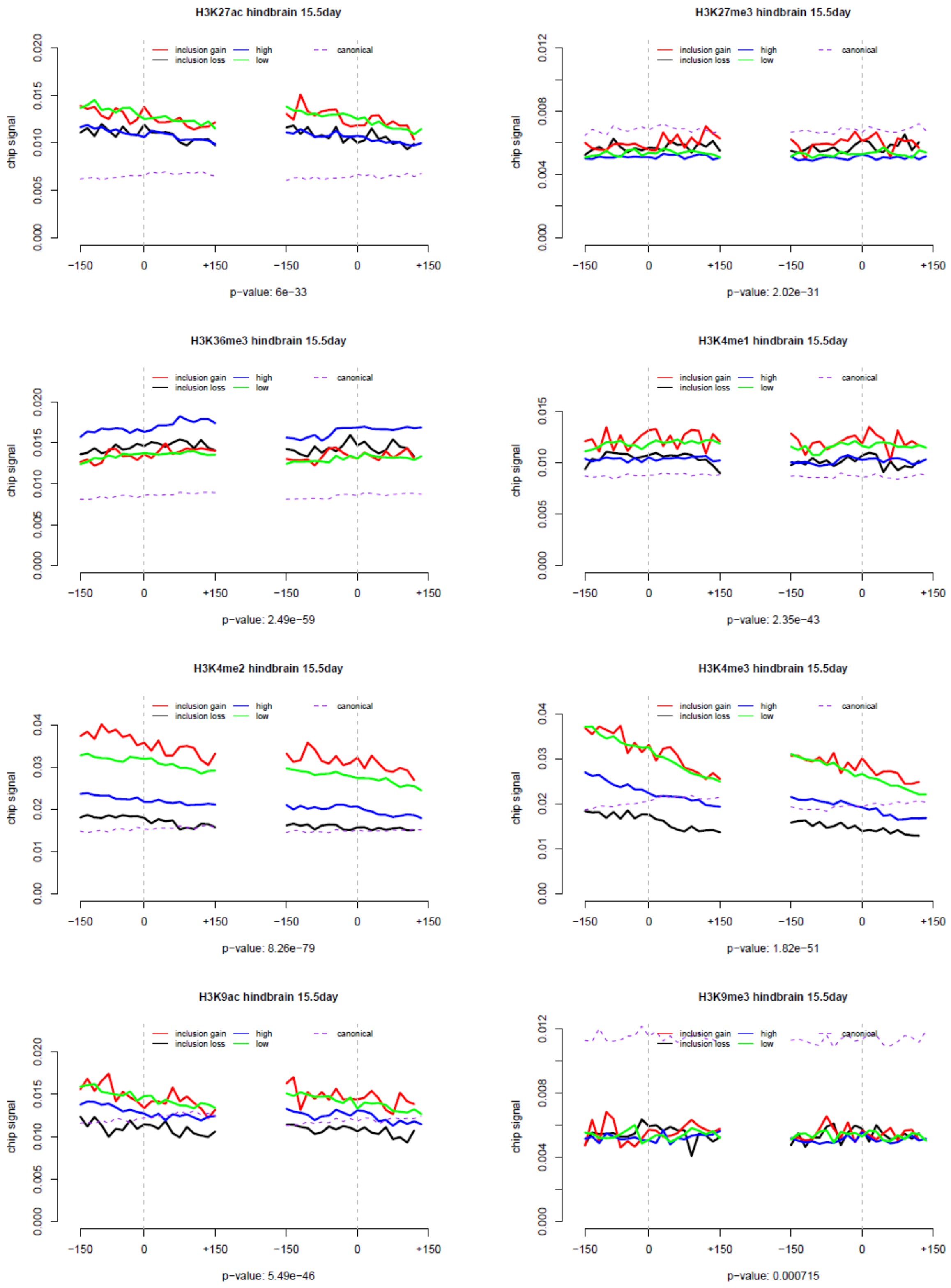

Supplemental figure 27: Representative distribution of mean ChIP-seq signal of 6 types of hPTM, including H3K36me3, H3K4me1, H3K9me3, H3K27ac, H3K4me2 and H3K4me3 on the flanking region (+/- 150bp) of four types of skipped exons in hindbrain at E15.5. Dashed grey line shows exon-intron borders.

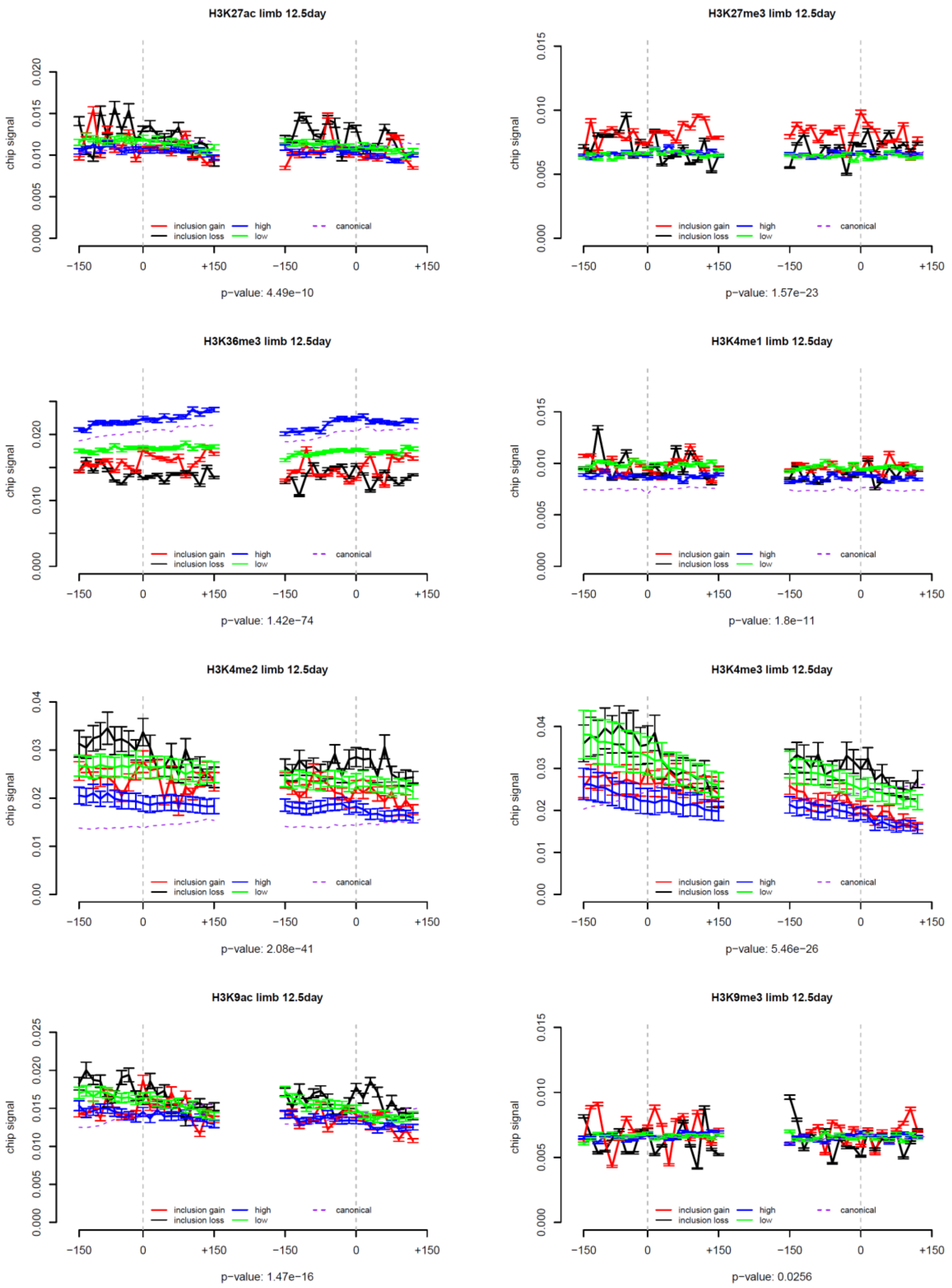

Supplemental figure 28: Representative distribution of mean ChIP-seq signal of 6 types of hPTM, including H3K36me3, H3K4me1, H3K9me3, H3K27ac, H3K4me2 and H3K4me3 on the flanking region (+/- 150bp) of four types of skipped exons in limb at E12.5. Dashed grey line shows exon-intron borders.

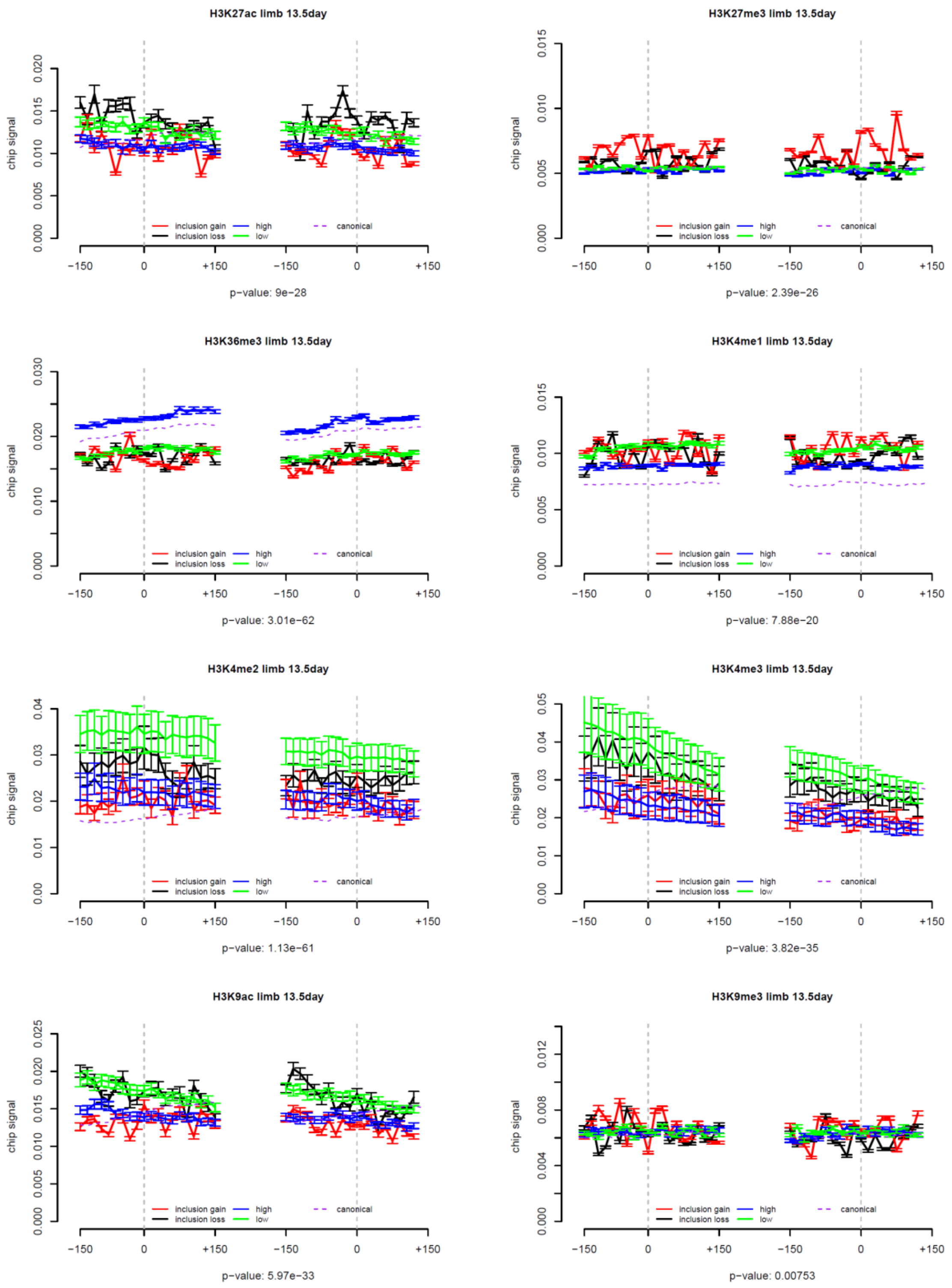

Supplemental figure 29: Representative distribution of mean ChIP-seq signal of 6 types of hPTM, including H3K36me3, H3K4me1, H3K9me3, H3K27ac, H3K4me2 and H3K4me3 on the flanking region (+/- 150bp) of four types of skipped exons in limb at 13.5day. Dashed grey line shows exon-intron borders.

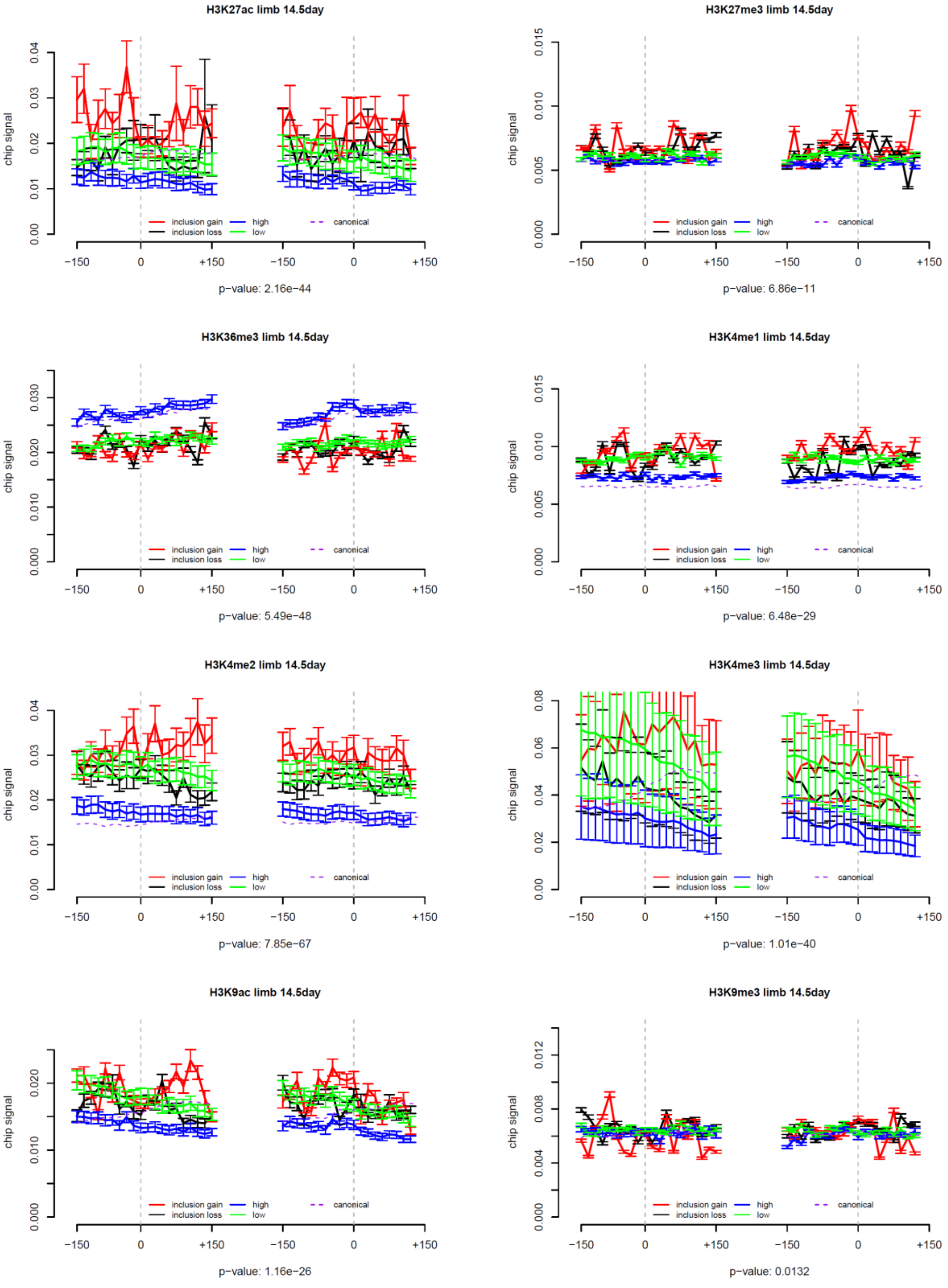

Supplemental figure 30: Representative distribution of mean ChIP-seq signal of 6 types of hPTM, including H3K36me3, H3K4me1, H3K9me3, H3K27ac, H3K4me2 and H3K4me3 on the flanking region (+/- 150bp) of four types of skipped exons in limb at E14.5. Dashed grey line shows exon-intron borders.

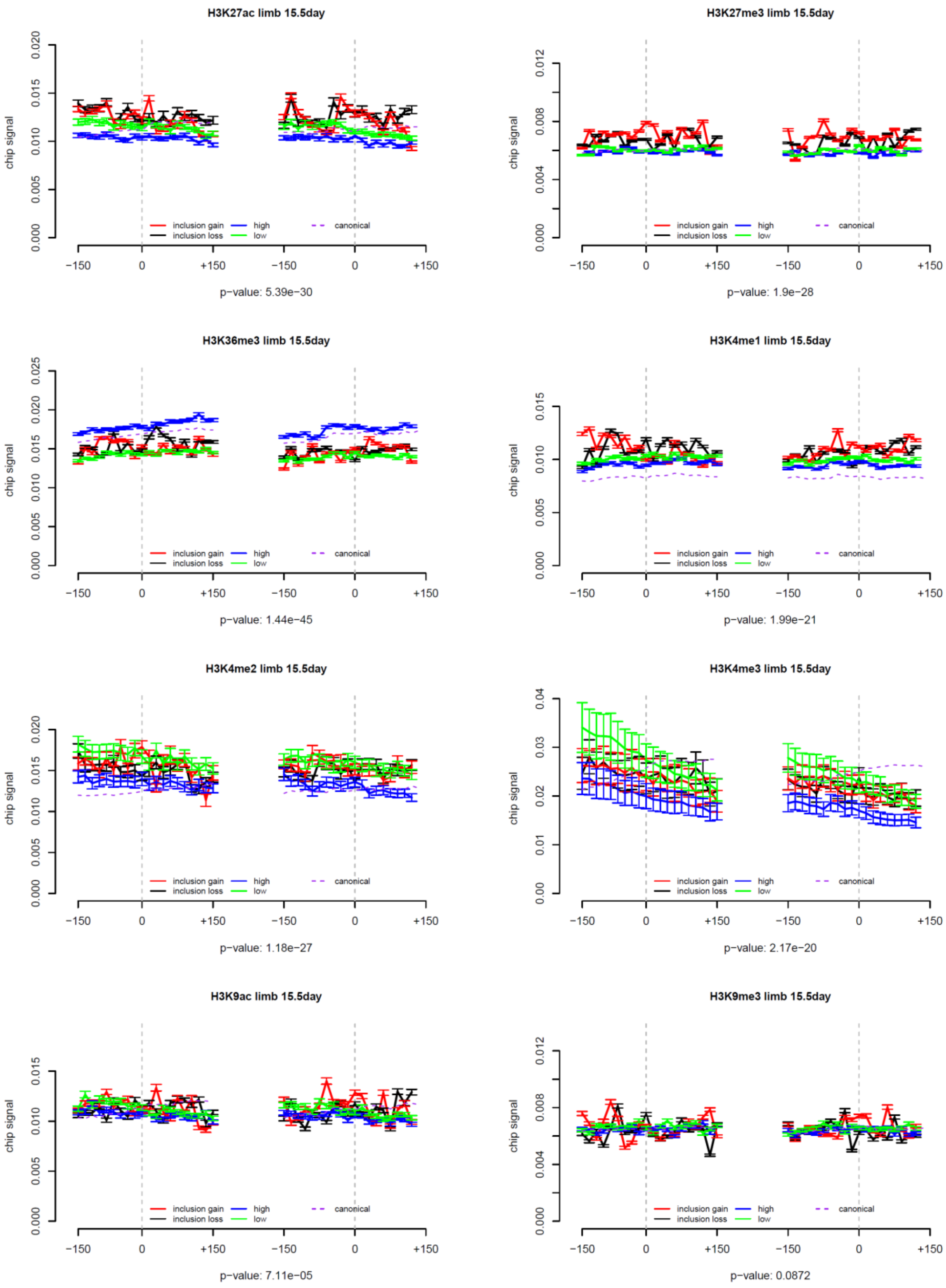

Supplemental figure 31: Representative distribution of mean ChIP-seq signal of 6 types of hPTM, including H3K36me3, H3K4me1, H3K9me3, H3K27ac, H3K4me2 and H3K4me3 on the flanking region (+/- 150bp) of four types of skipped exons in midbrain at E15.5. Dashed grey line shows exon-intron borders.

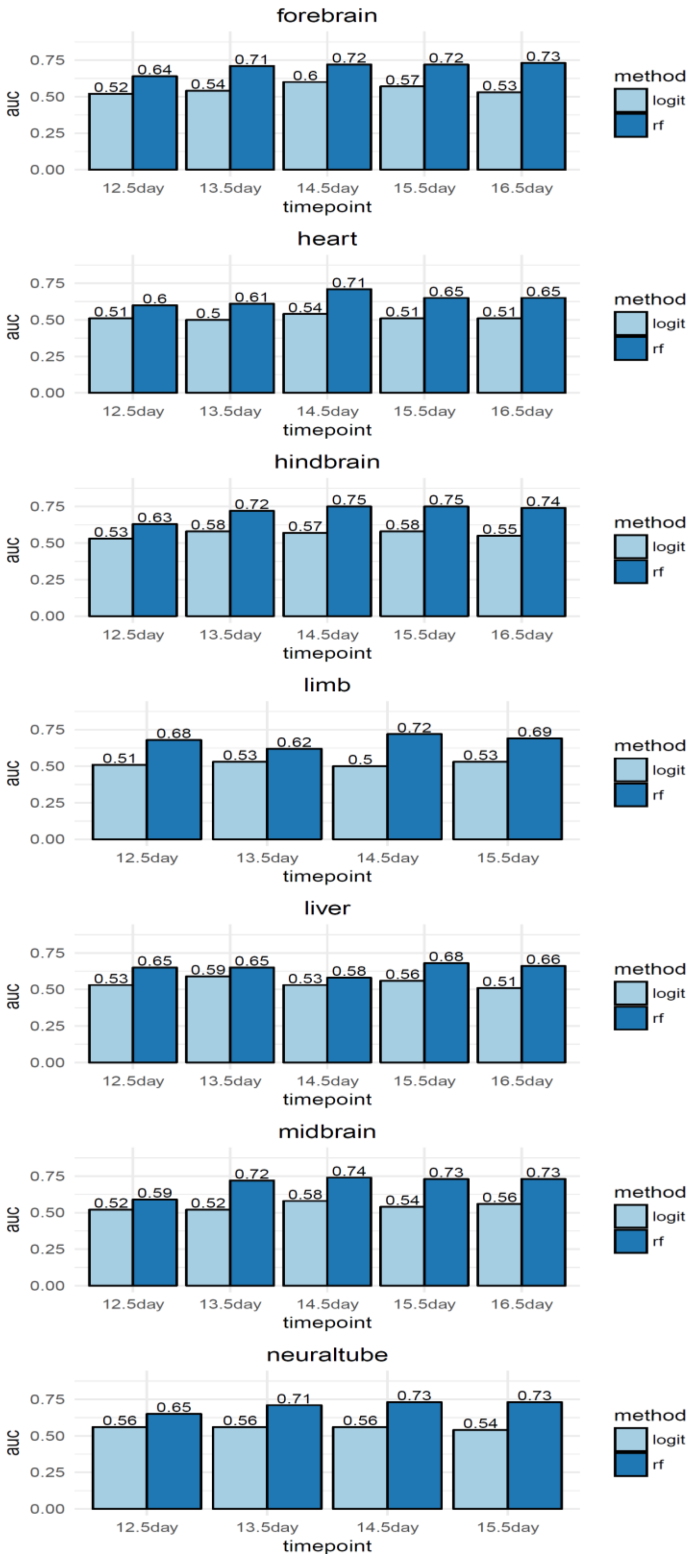

Supplemental figure 32: Model performance for isoform selected high/low exons

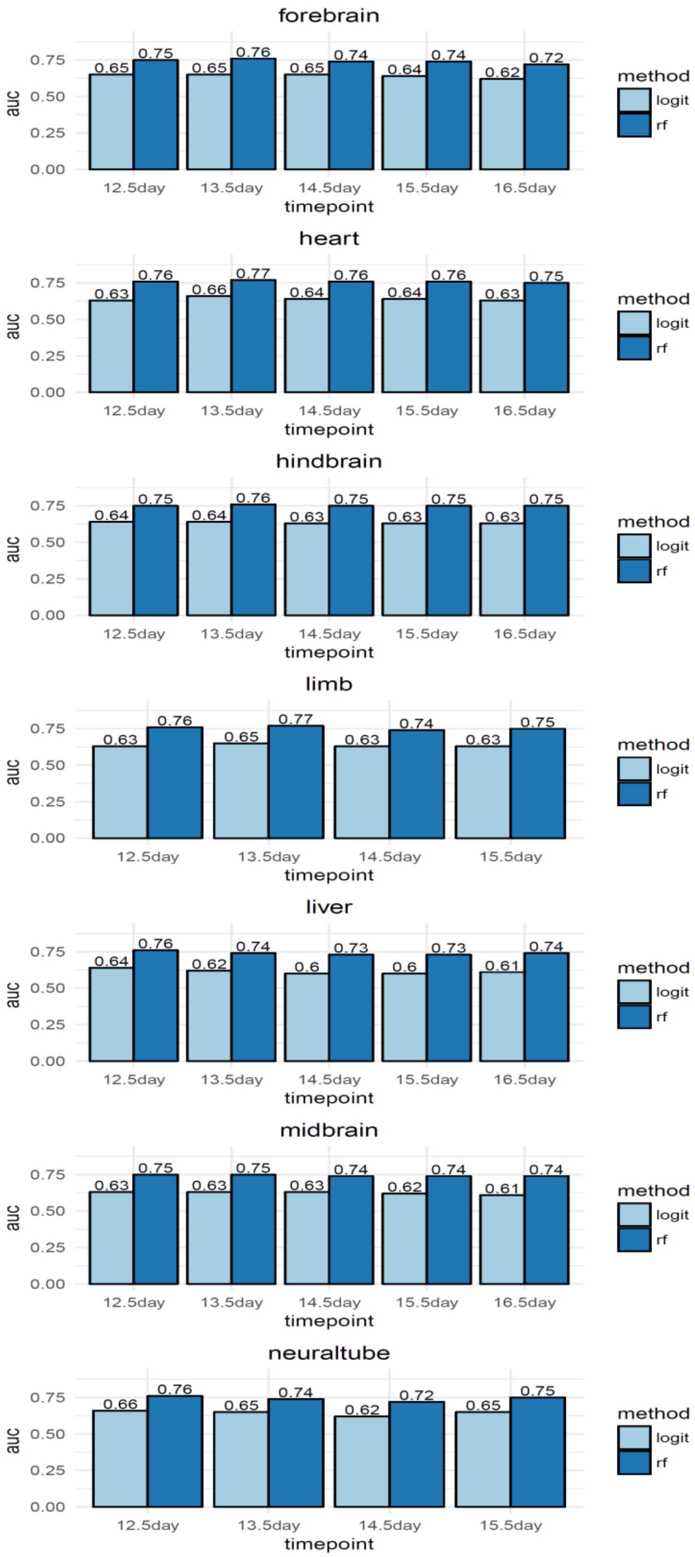

Supplemental figure 33: Model performance for developmental gain/loss exons

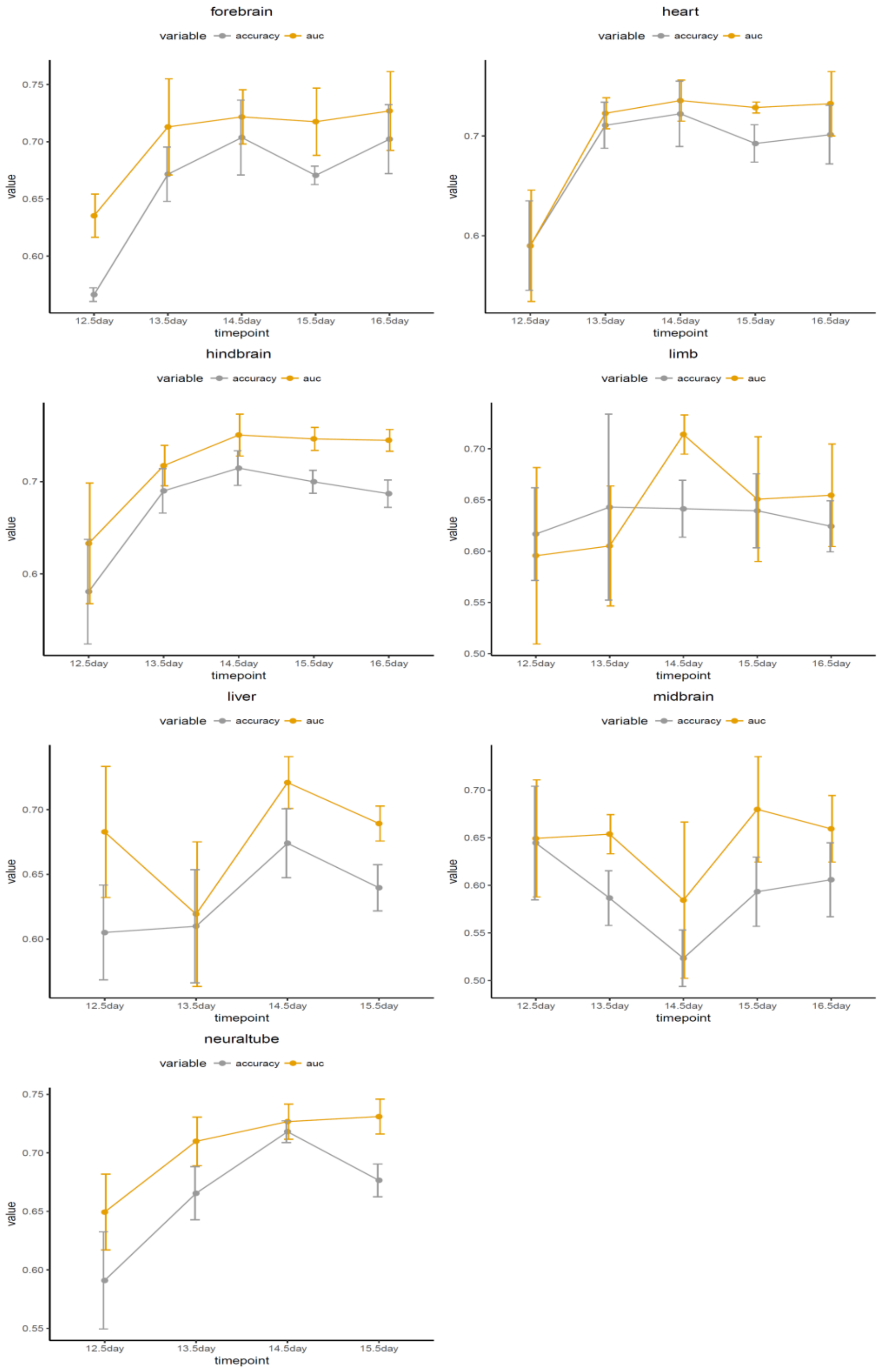

Supplemental figure 34: Random forest model performance for isoform selected high/low exons

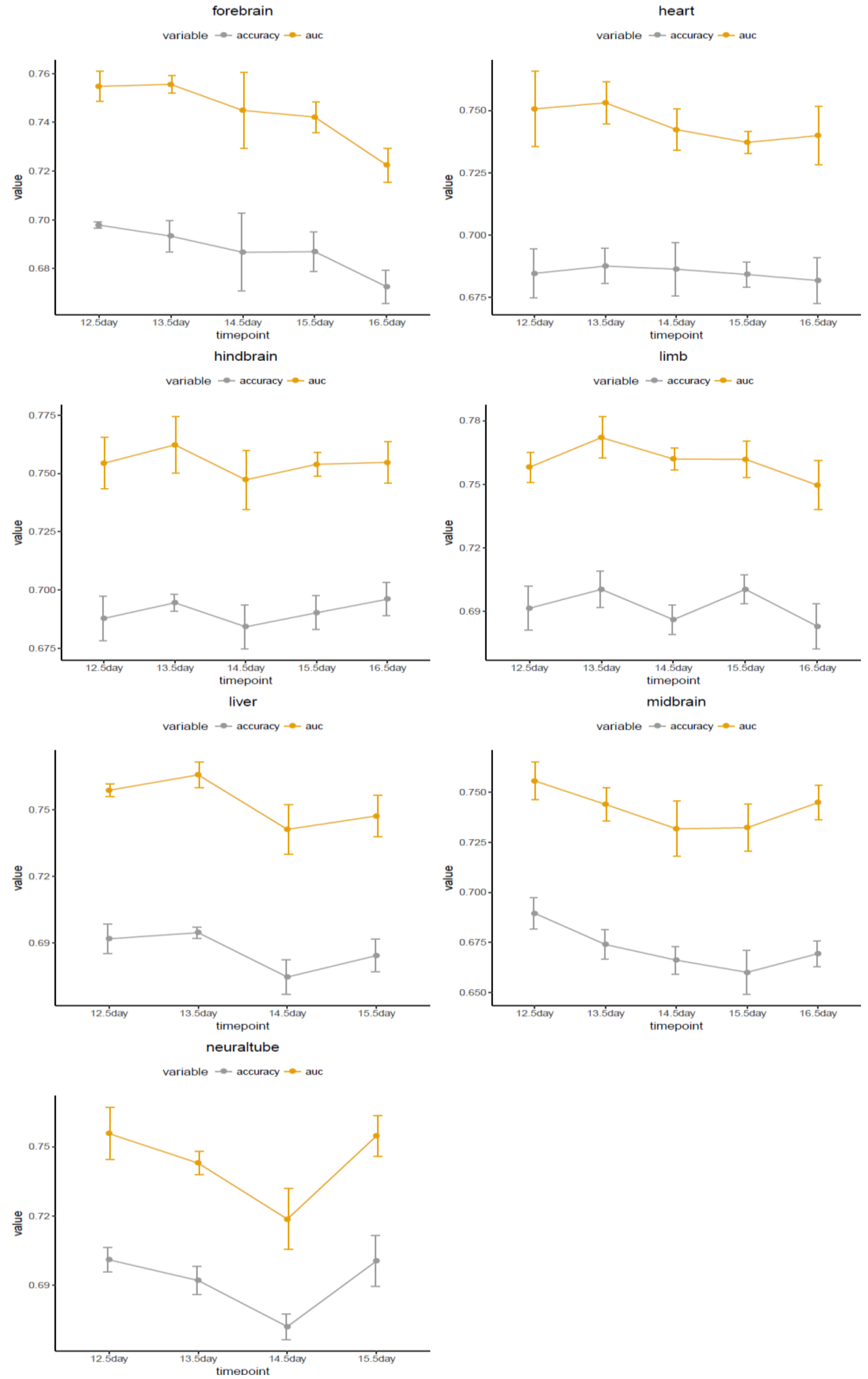

Supplemental figure 35: Random forest model performance for developmental gain/loss exons

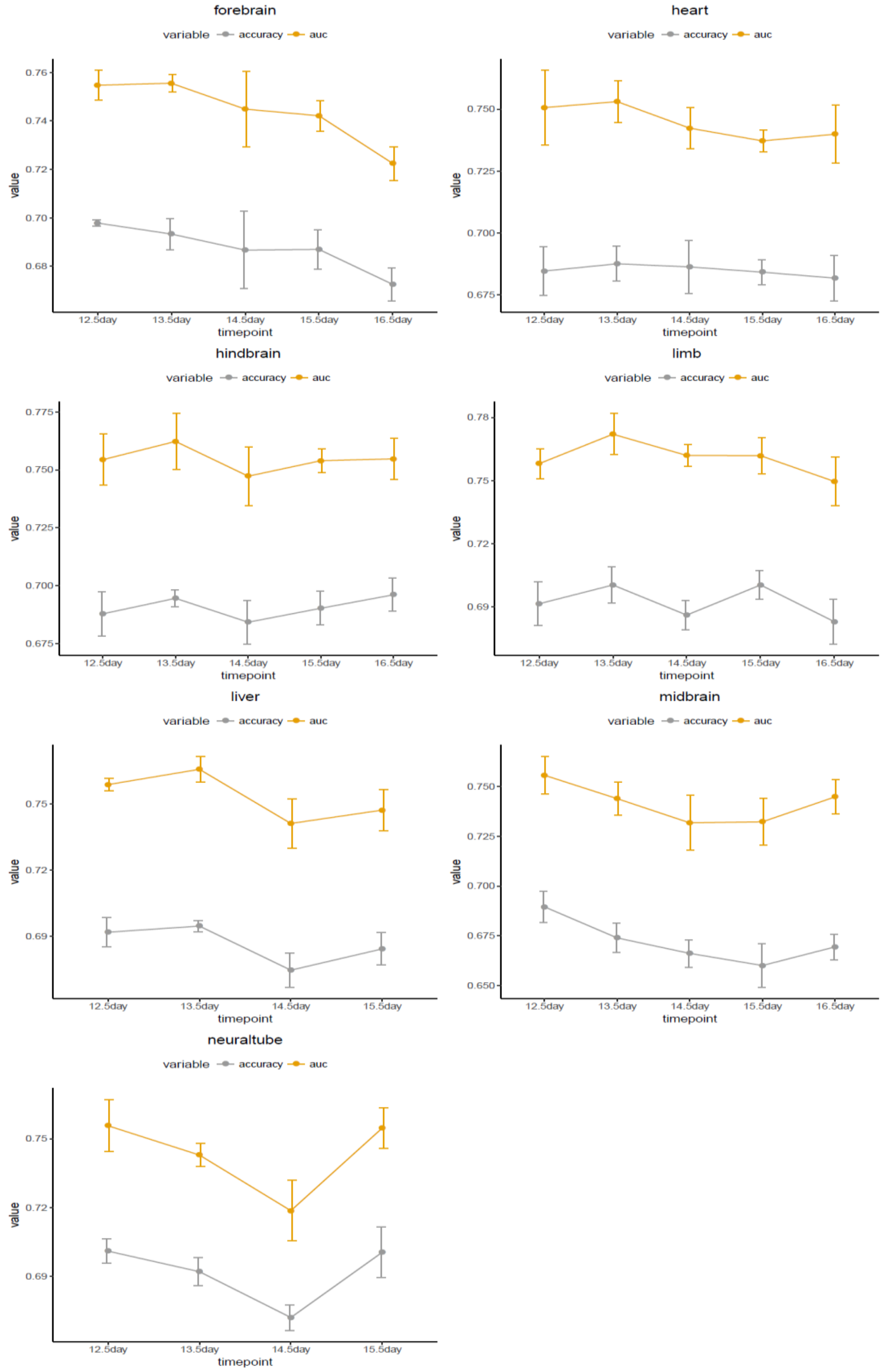

Supplemental figure 36: Random forest model performance for developmental gain/loss exons

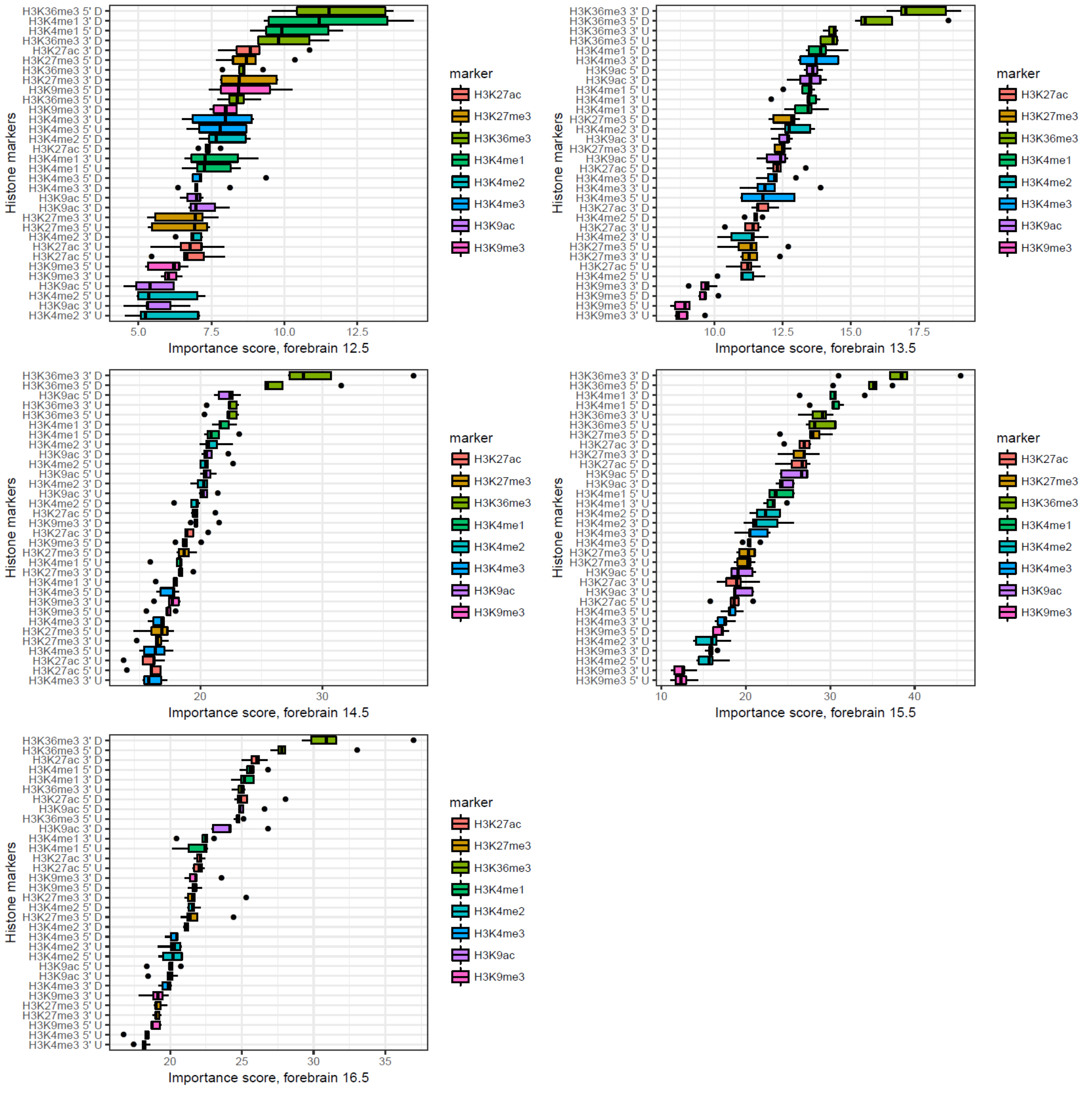

Supplemental figure 37: Boxplot of important score generated by random forest model in forebrain at different timepoints for developmental gain/loss shows several types of hPTMs are key predictors. Importance score is calculated based on 5-fold cross validation.

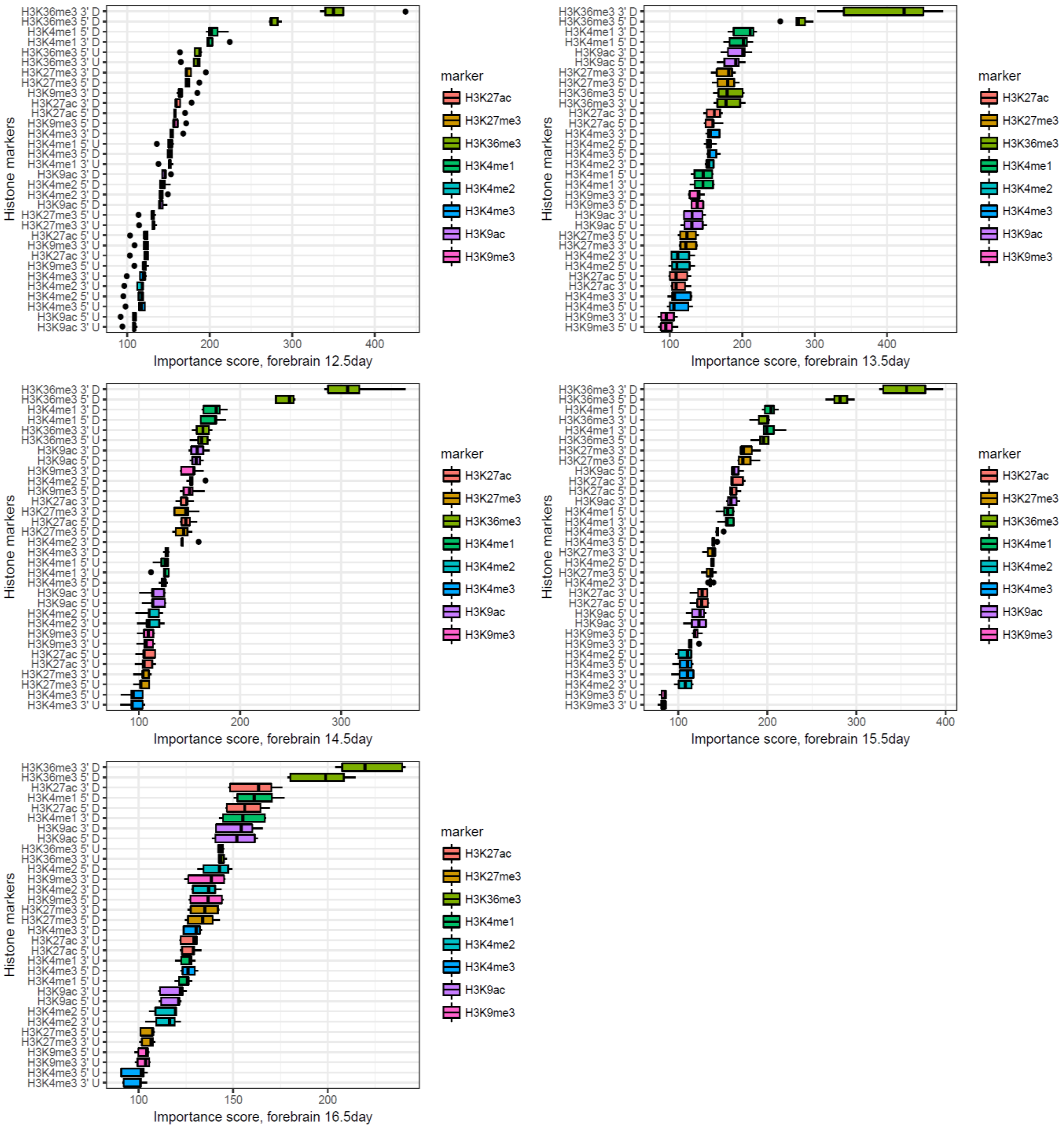

Supplemental figure 38: Boxplot of important score generated by random forest model in forebrain at different timepoints for isoform selected high/low shows several types of hPTMs are key predictors. Importance score is calculated based on 5-fold cross validation.

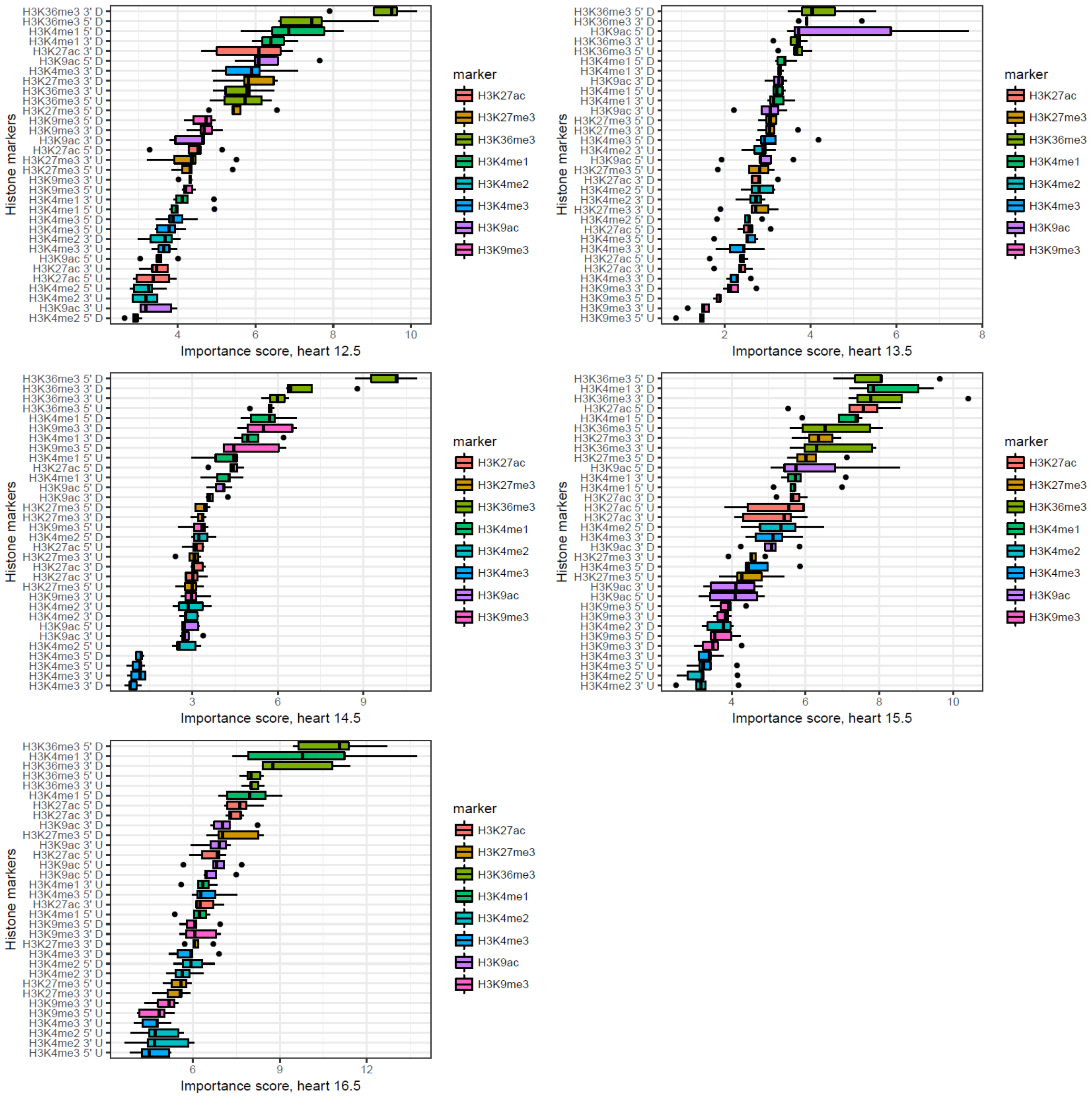

Supplemental figure 39: Boxplot of important score generated by random forest model in heart at different time points for developmental gain/loss shows several types of hPTMs are key predictors. Importance score is calculated based on 5-fold cross validation.

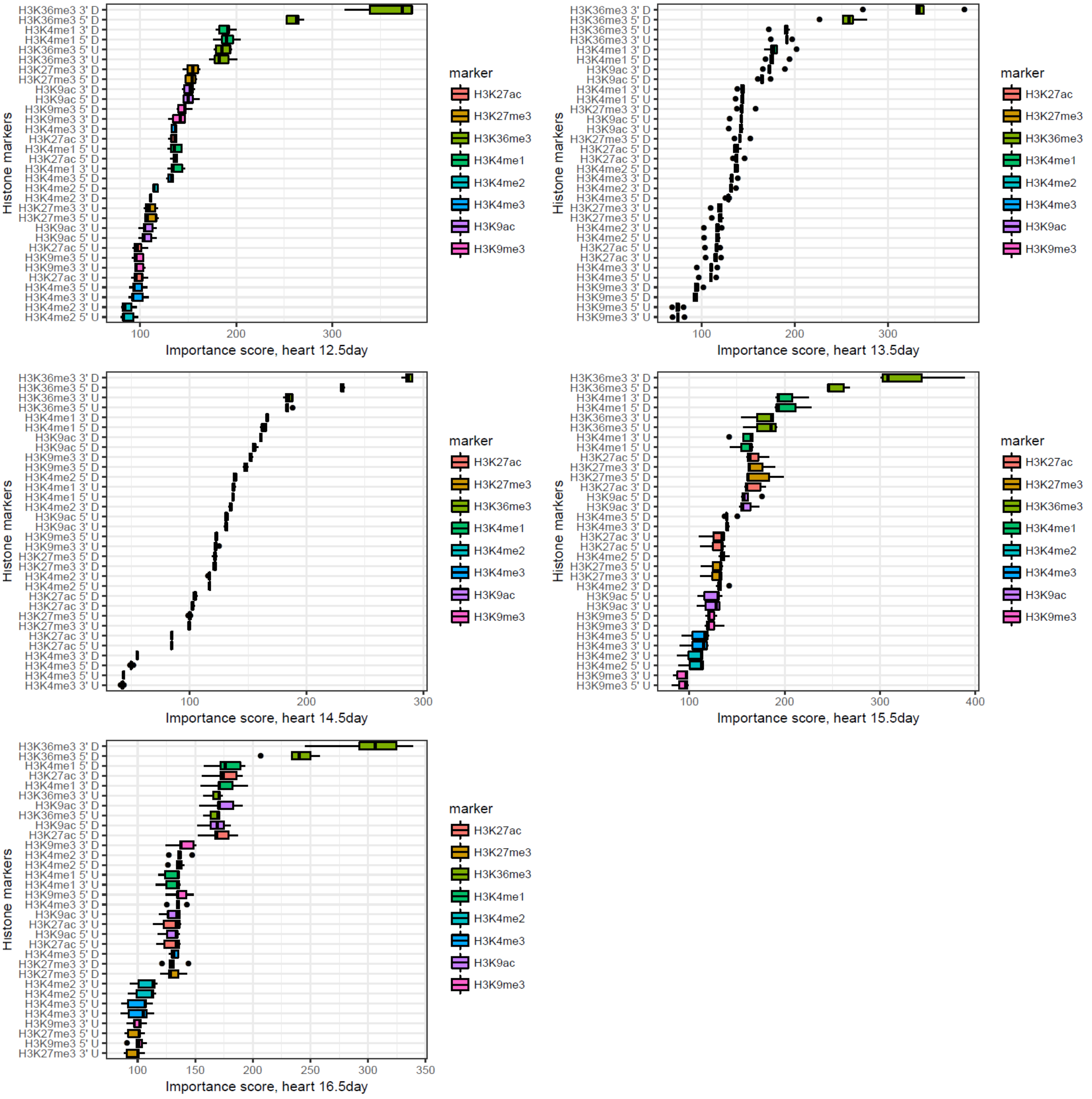

Supplemental figure 40: Boxplot of important score generated by random forest model in heart at different time points for isoform selected high/low shows several types of hPTMs are key predictors. Importance score is calculated based on 5-fold cross validation.

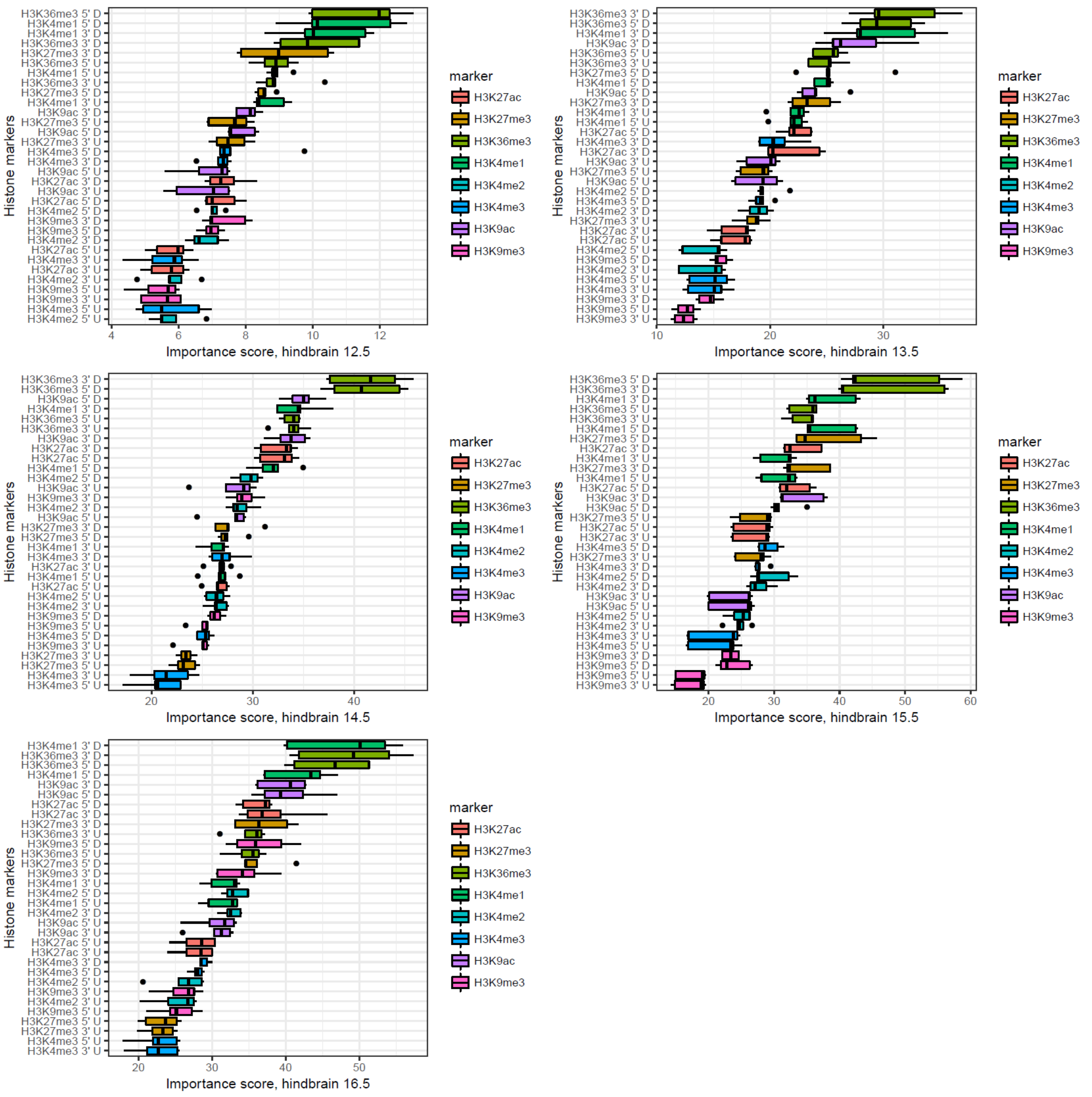

Supplemental figure 41: Boxplot of important score generated by random forest model in hindbrain at different time points for developmental gain/loss shows several types of hPTMs are key predictors. Importance score is calculated based on 5-fold cross validation.

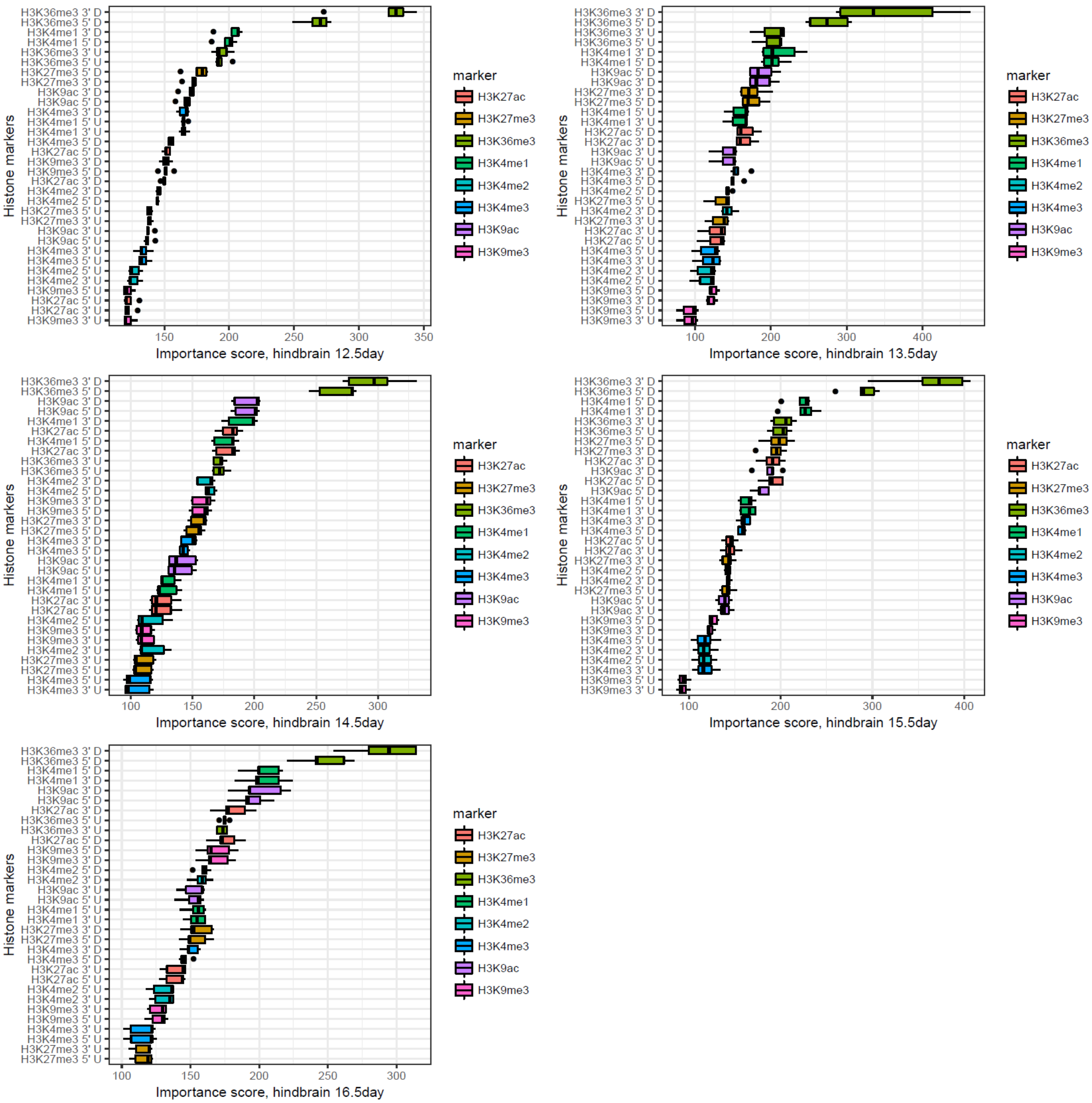

Supplemental figure 42: Boxplot of important score generated by random forest model in hindbrain at different time points for isoform selected high/low shows several types of hPTMs are key predictors. Importance score is calculated based on 5-fold cross validation.

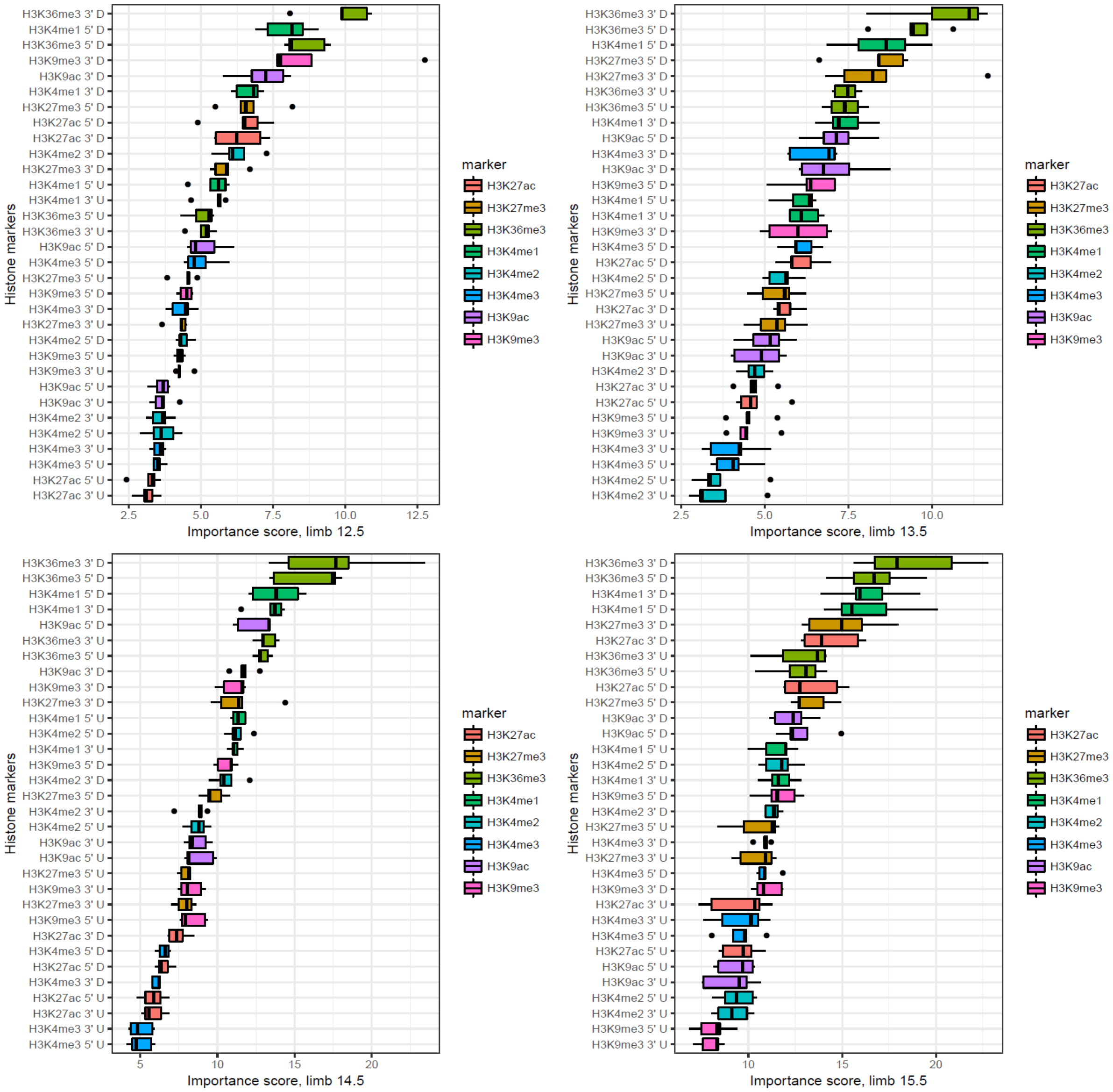

Supplemental figure 43: Boxplot of important score generated by random forest model in limb at different time points for developmental gain/loss shows several types of hPTMs are key predictors. Importance score is calculated based on 5-fold cross validation.

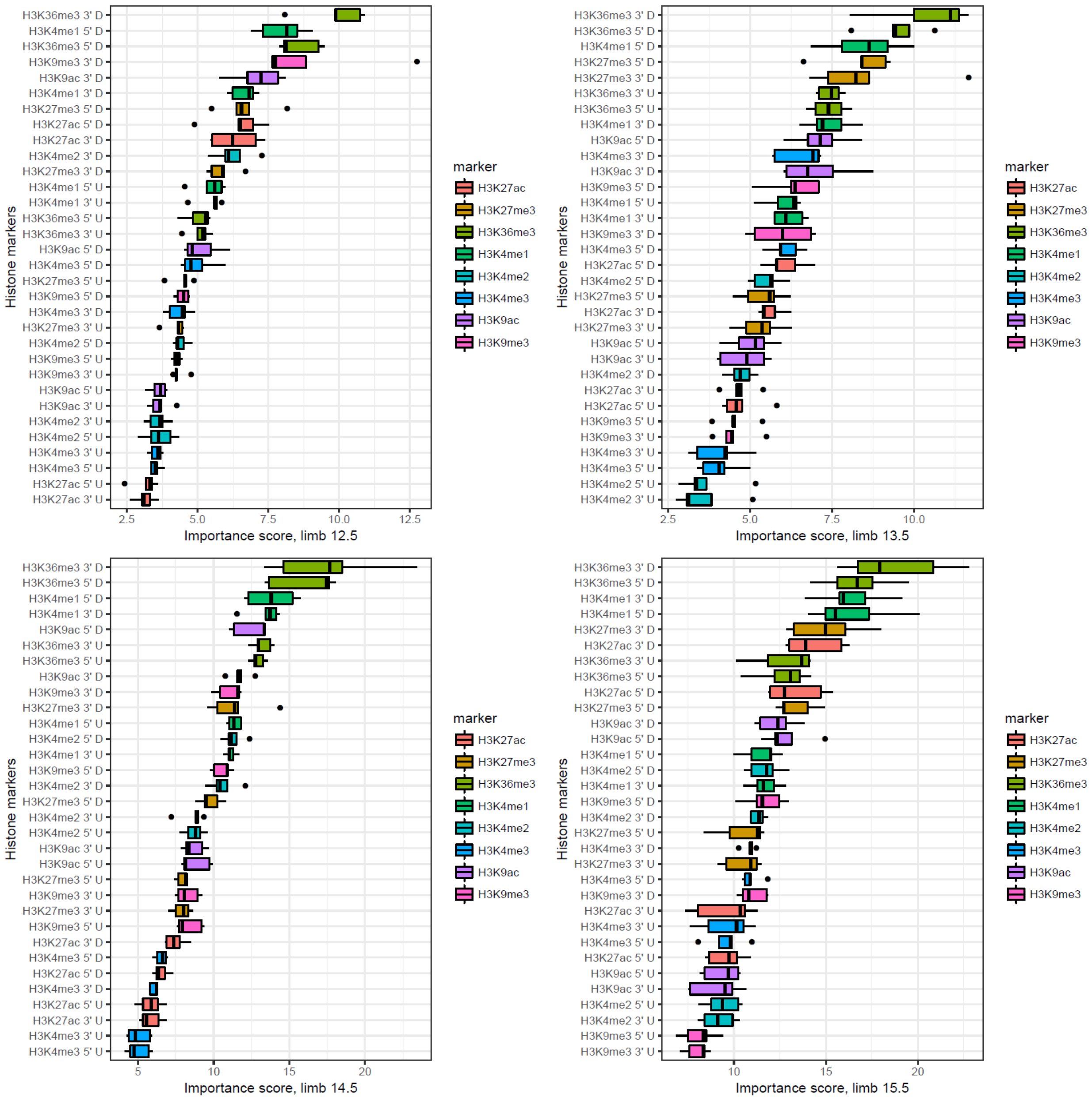

Supplemental figure 44: Boxplot of important score generated by random forest model in limb at different time points for isoform selected high/low shows several types of hPTMs are key predictors. Importance score is calculated based on 5-fold cross validation.

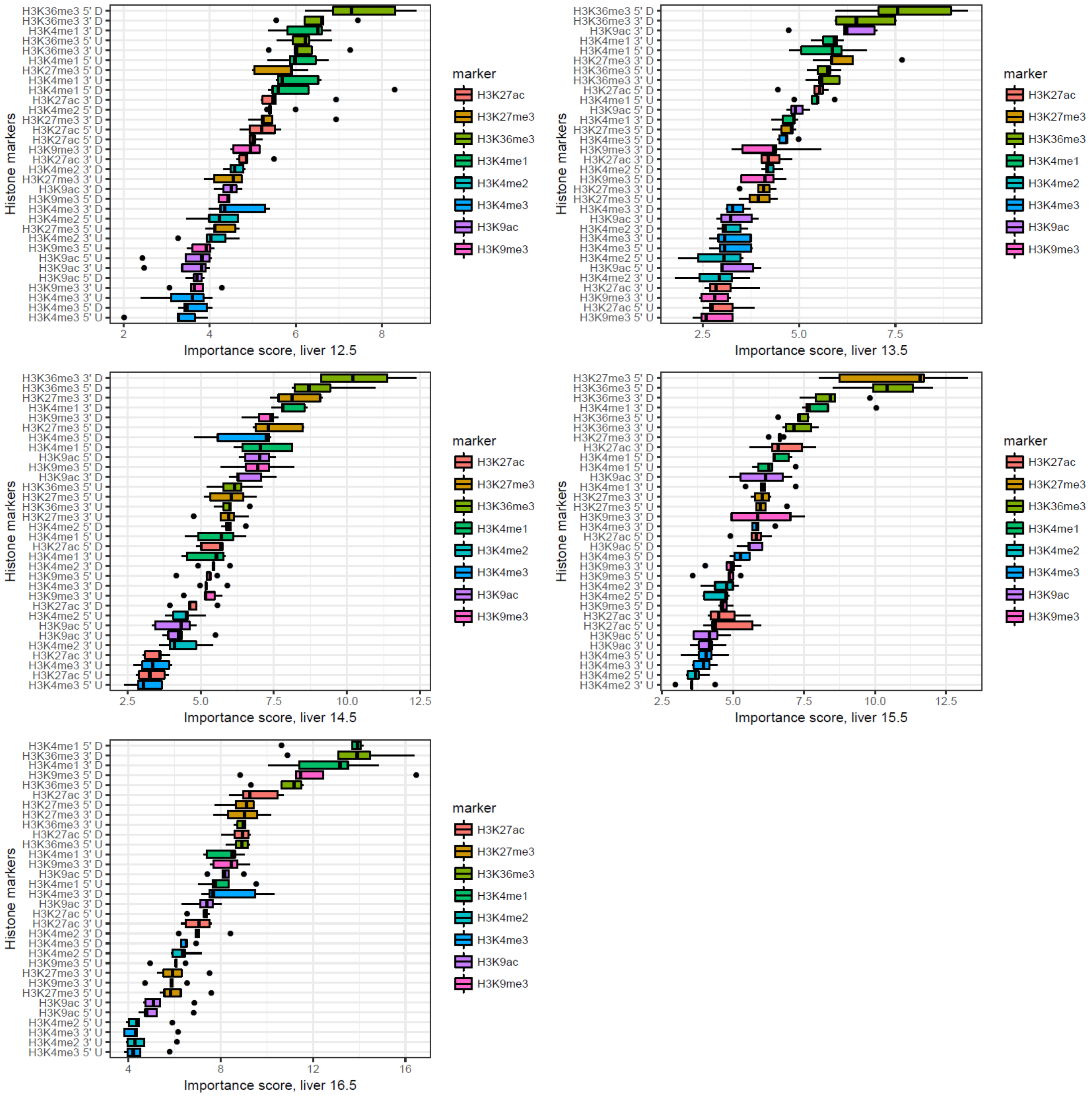

Supplemental figure 45: Boxplot of important score generated by random forest model in liver at different time points for developmental gain/loss shows several types of hPTMs are key predictors. Importance score is calculated based on 5-fold cross validation.

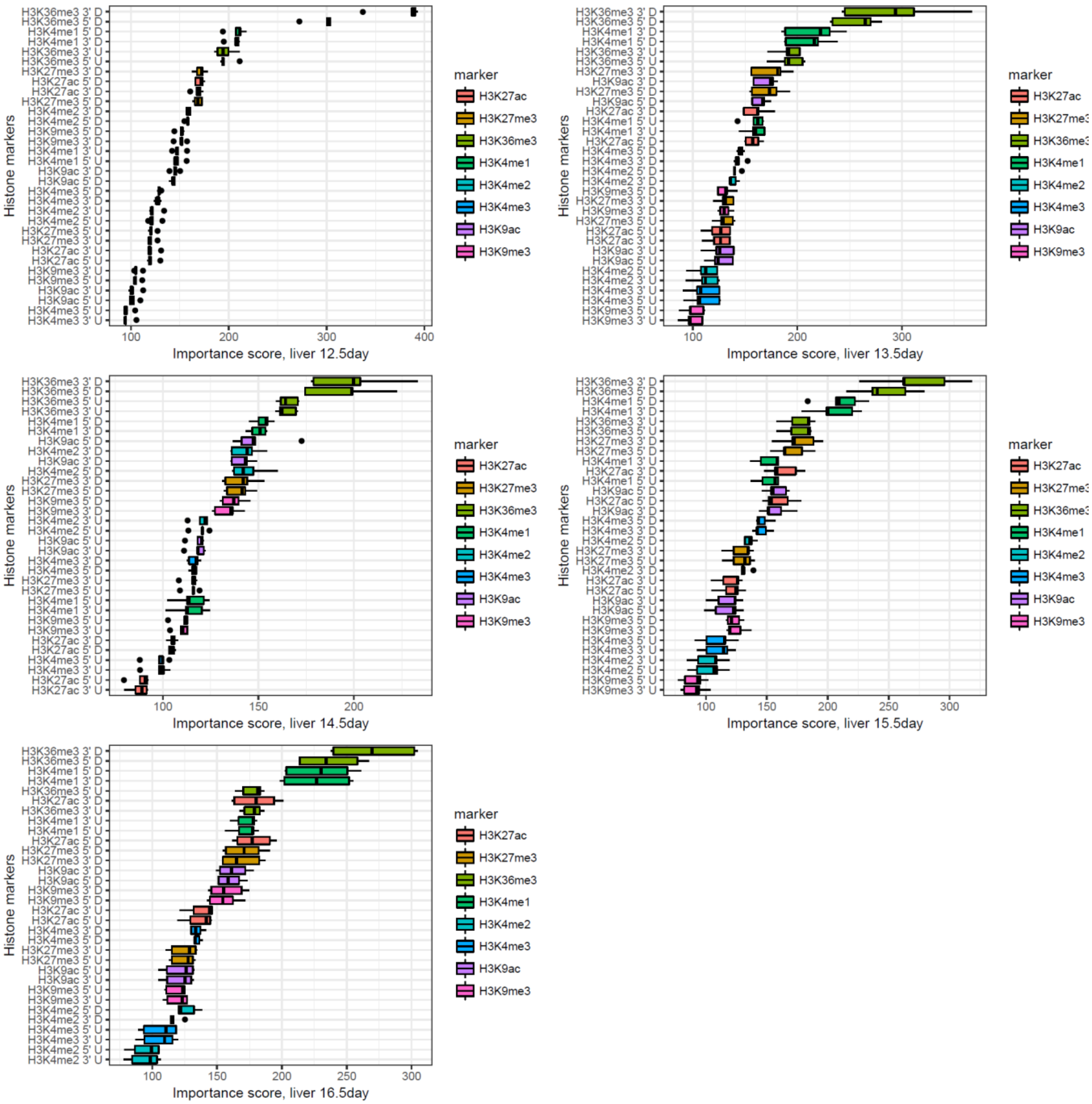

Supplemental figure 46: Boxplot of important score generated by random forest model in liver at different time points for isoform selected high/low shows several types of hPTMs are key predictors. Importance score is calculated based on 5-fold cross validation.

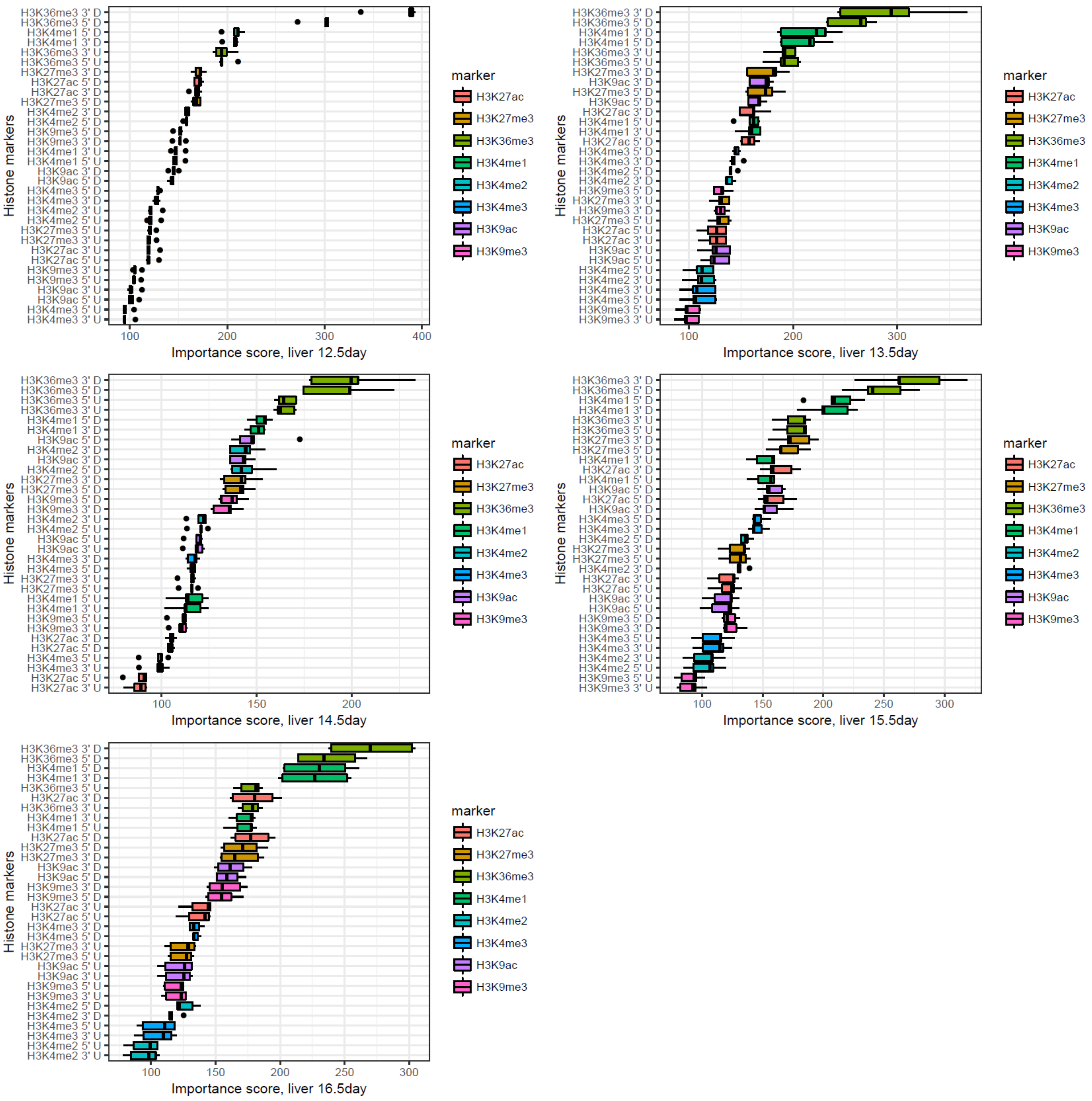

Supplemental figure 47: Boxplot of important score generated by random forest model in liver at different time points for developmental gain/loss shows several types of hPTMs are key predictors. Importance score is calculated based on 5-fold cross validation.

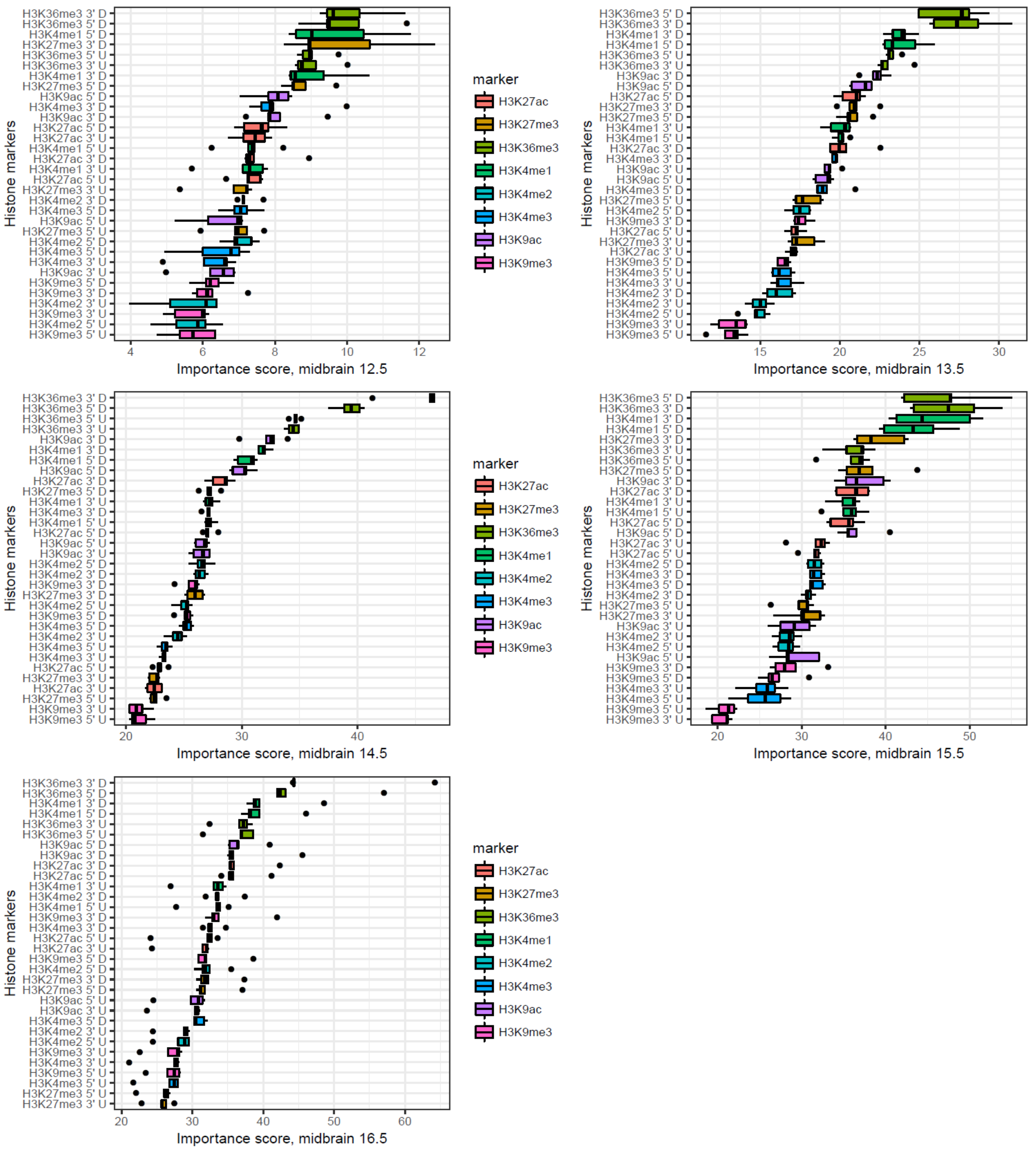

Supplemental figure 48: Boxplot of important score generated by random forest model in midbrain at different time points for isoform selected high/low shows several types of hPTMs are key predictors. Importance score is calculated based on 5-fold cross validation.

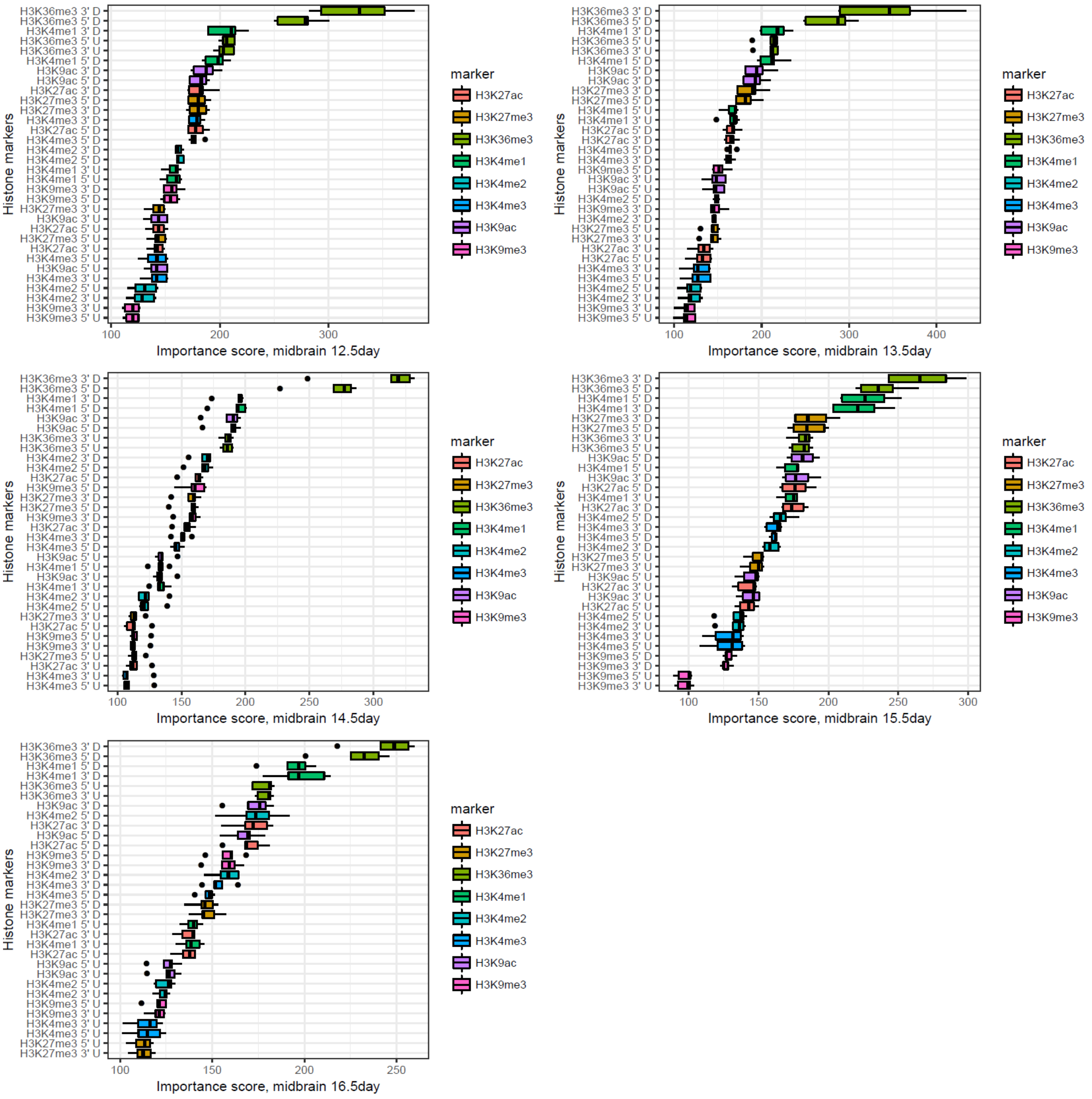

Supplemental figure 49: Boxplot of important score generated by random forest model in midbrain at different time points for developmental gain/loss shows several types of hPTMs are key predictors. Importance score is calculated based on 5-fold cross validation.

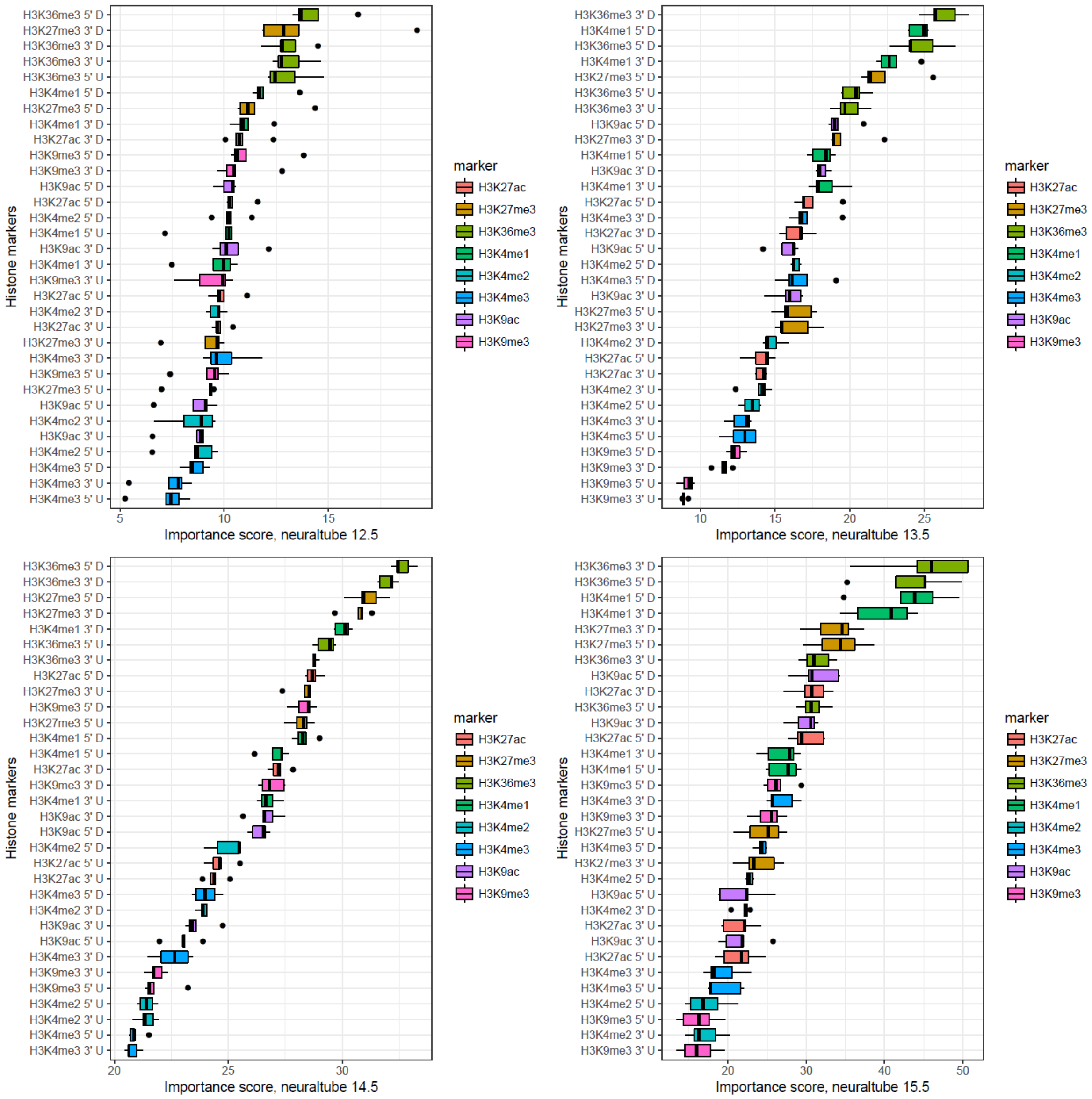

Supplemental figure 50: Boxplot of important score generated by random forest model in neuraltube at different time points for isoform selected high/low shows several types of hPTMs are key predictors. Importance score is calculated based on 5-fold cross validation.

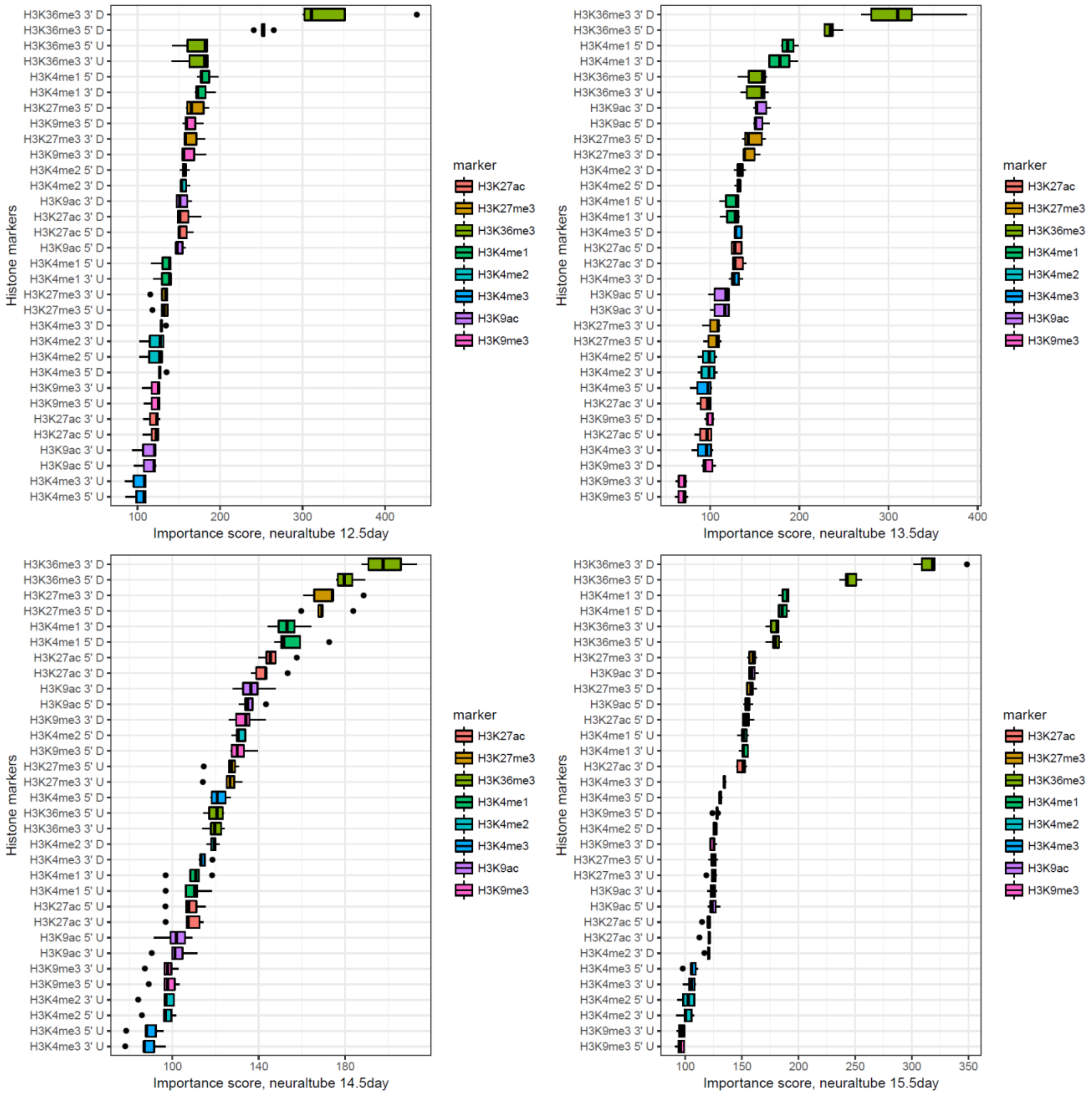

Supplemental figure 51: Boxplot of important score generated by random forest model in neuraltube at different time points for developmental gain/loss shows several types of hPTMs are key predictors. Importance score is calculated based on 5-fold cross validation.

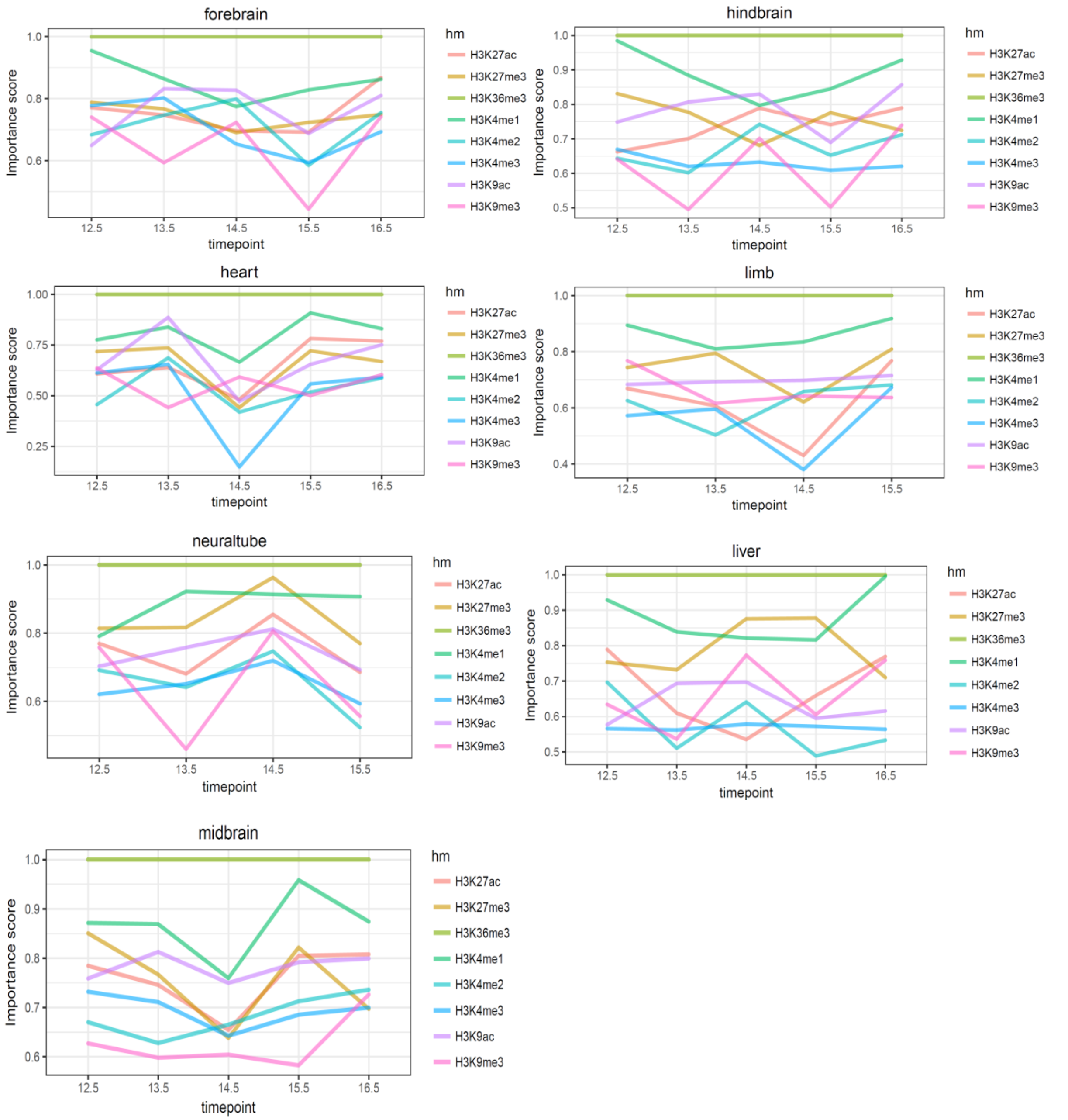

Supplemental figure 52: Contributions of different types of hPTMs to differentiate developmental gain versus developmental loss over time in 7 tissues.

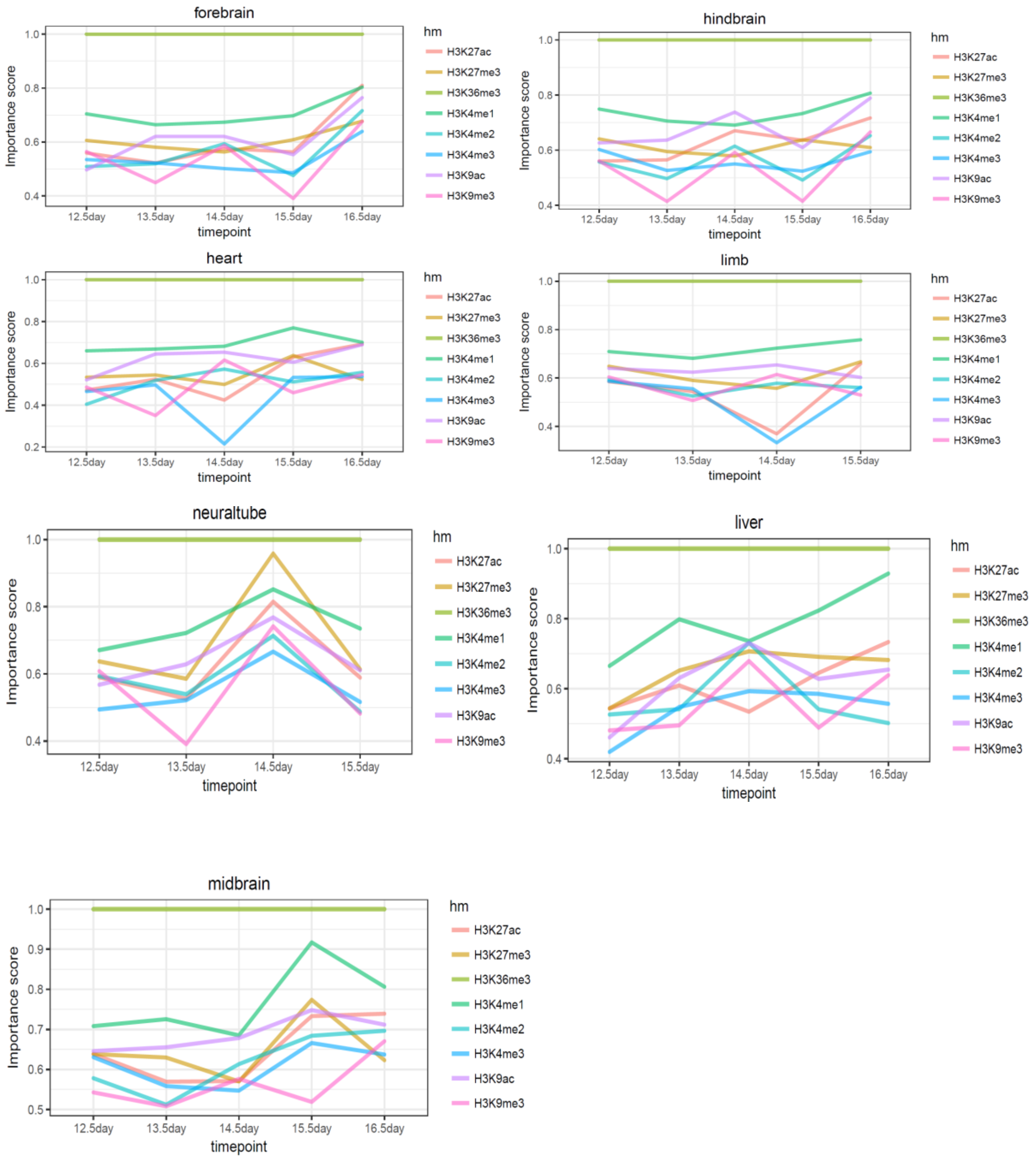

Supplemental figure 53: Contributions of different types of hPTMs to differentiate developmental gain versus developmental loss over time in 7 tissues.

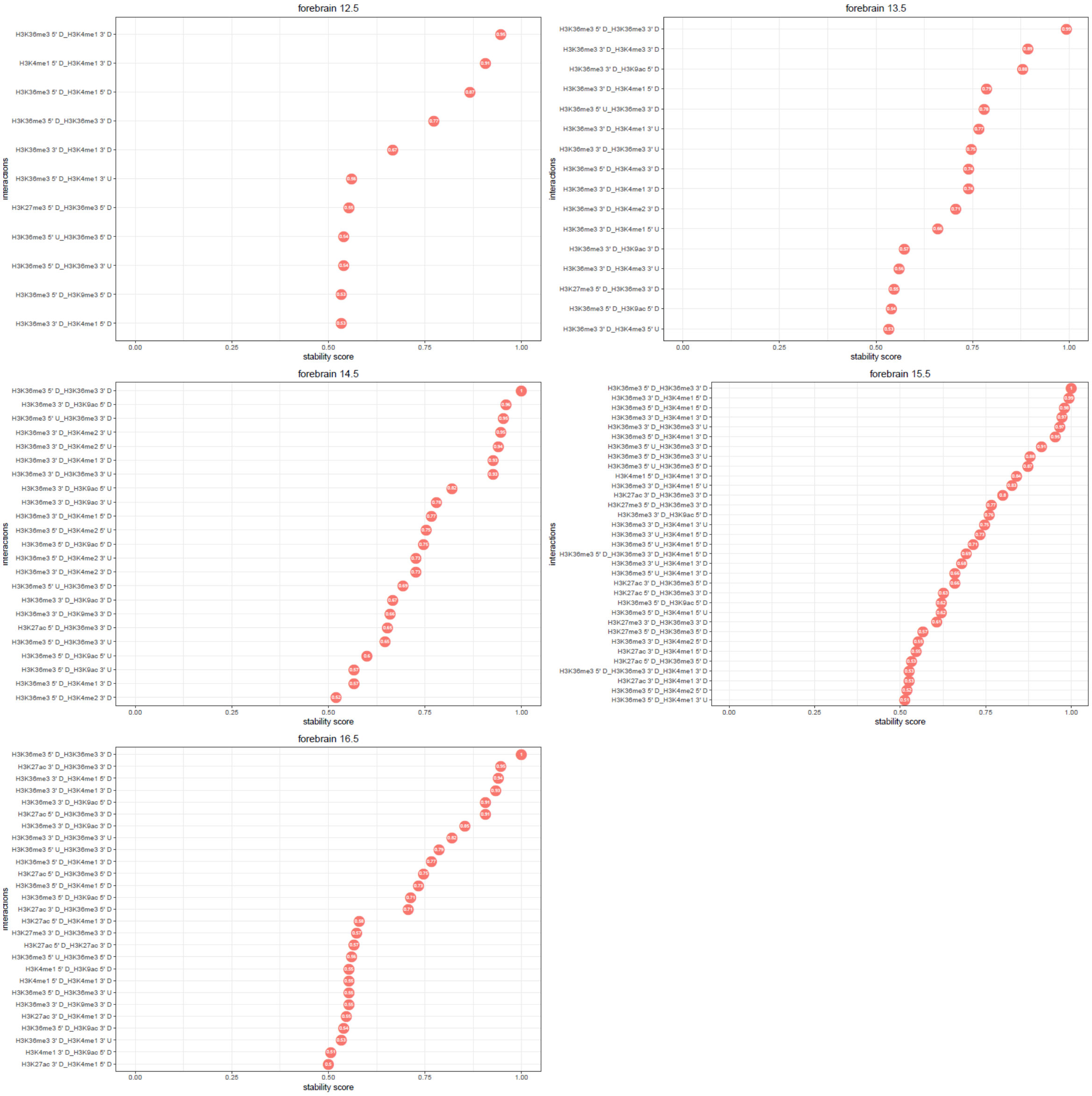

Supplemental figure 54: Contributions of different types of hPTMs to differentiate developmental gain versus developmental loss over time in forebrain.

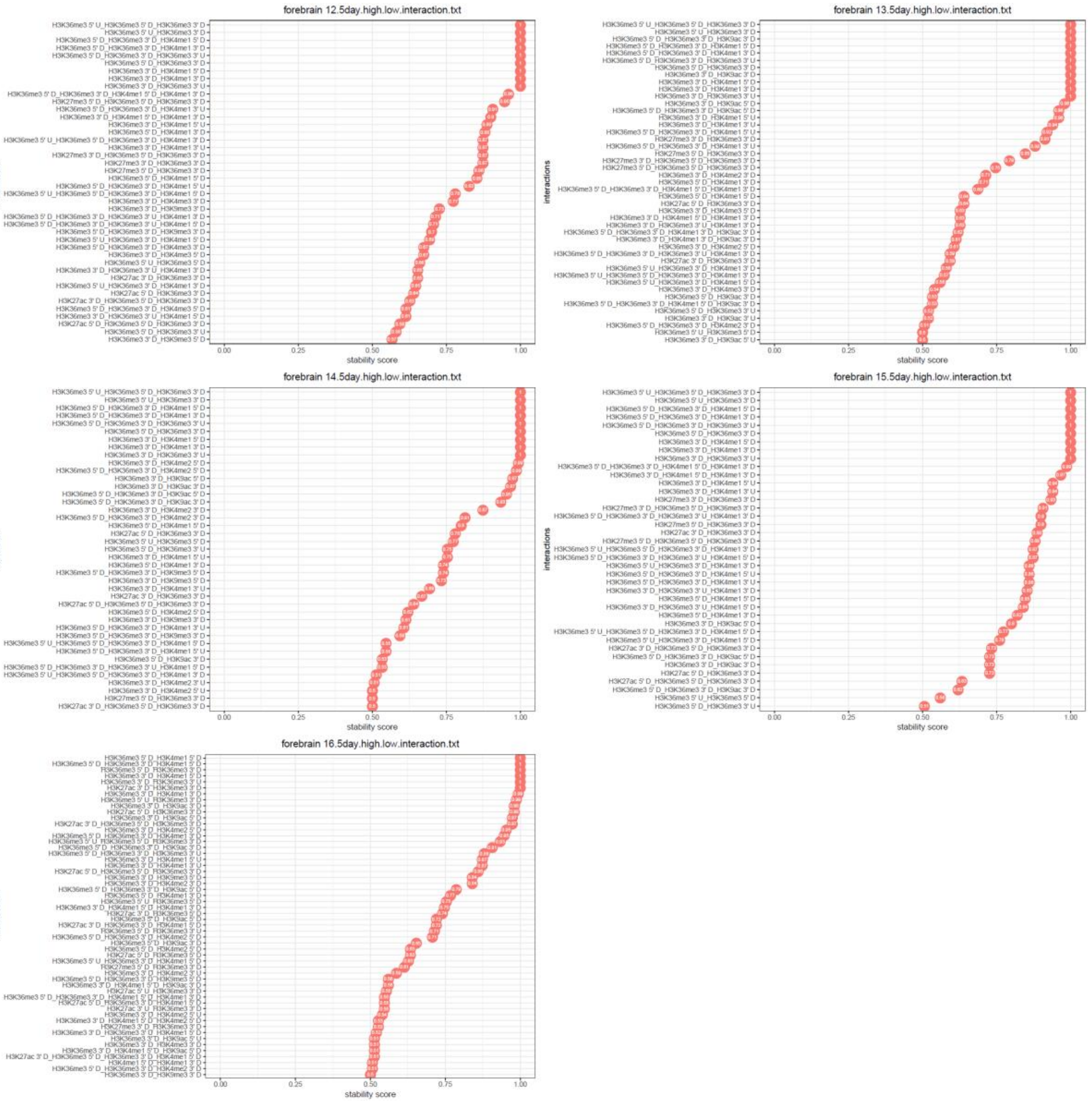

Supplemental figure 55: Contributions of different types of hPTMs to differentiate isoform selected high versus low over time in forebrain.

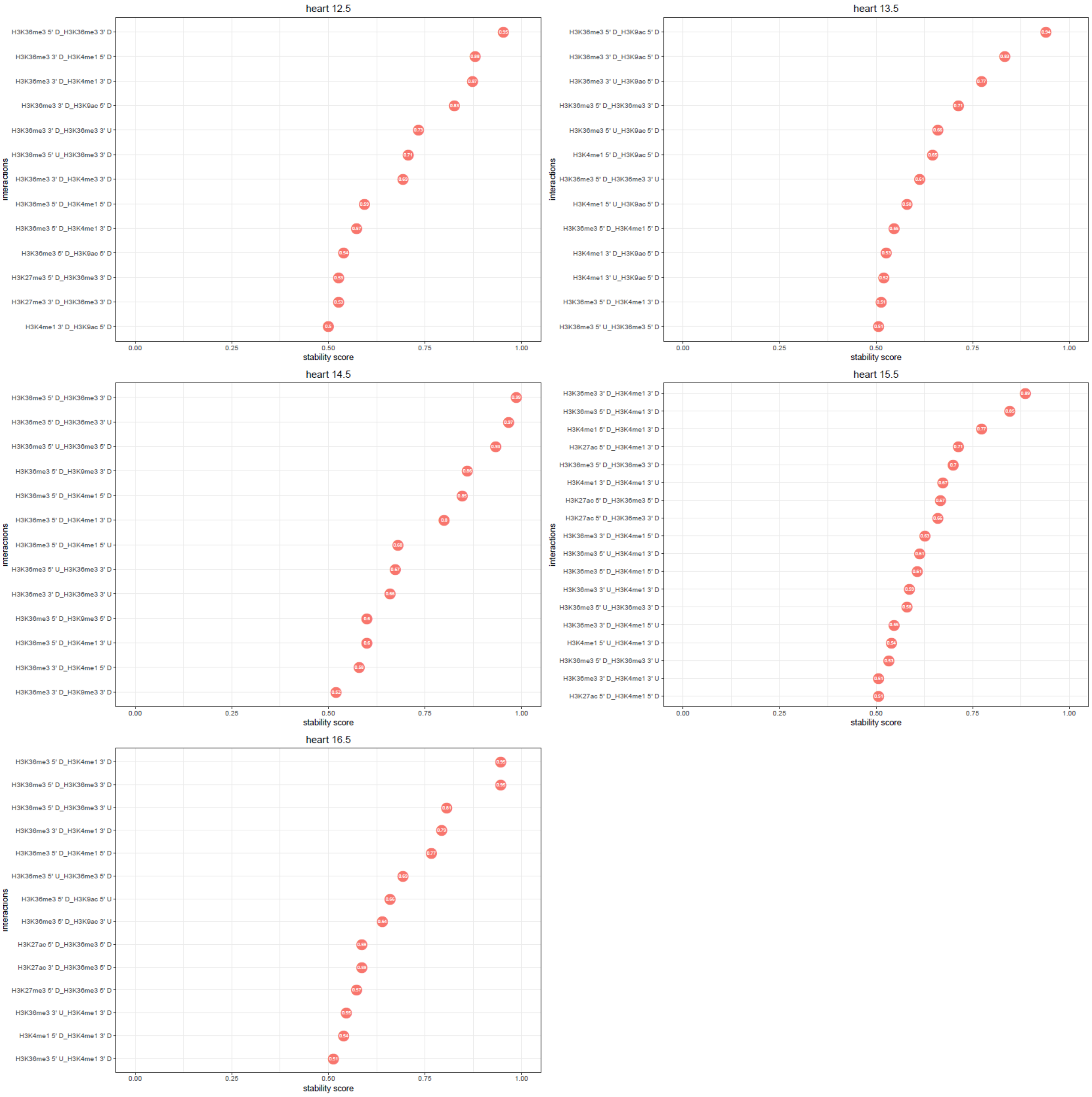

Supplemental figure 56: Contributions of different types of hPTMs to differentiate developmental gain versus loss over time in heart.

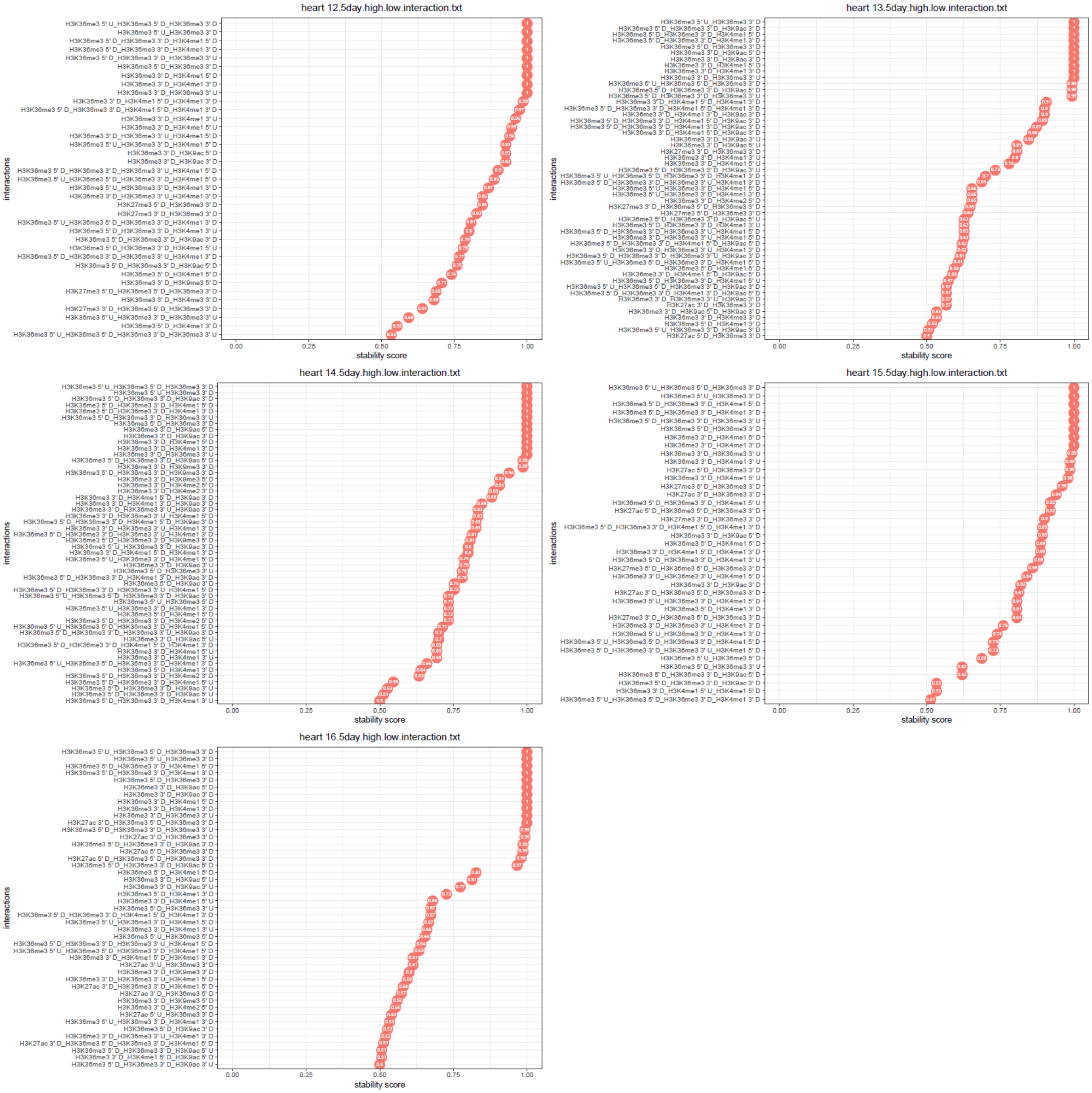

Supplemental figure 57: Contributions of different types of hPTMs to differentiate isoform selected high versus low over time in heart.

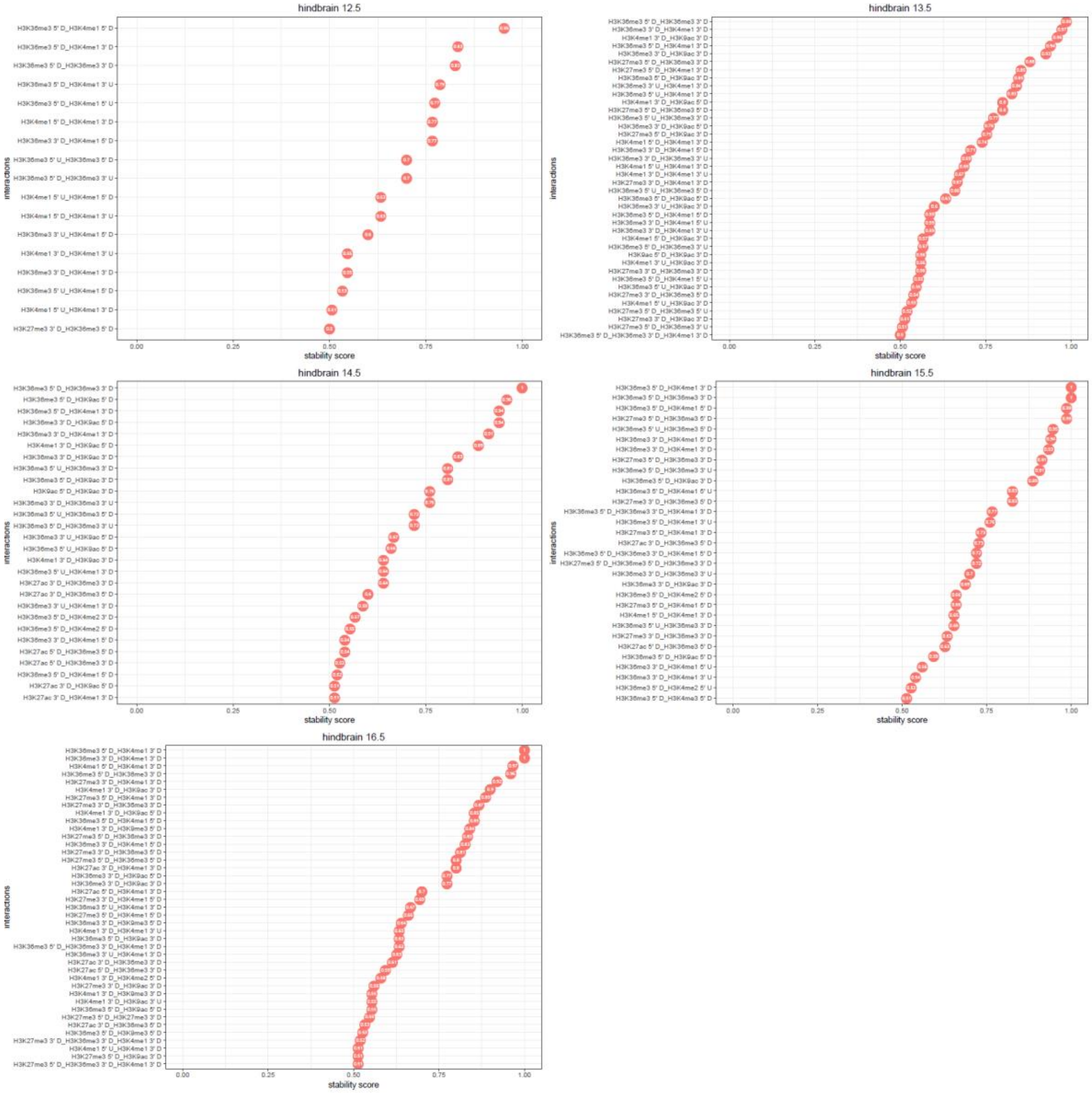

Supplemental figure 58: Contributions of different types of hPTMs to differentiate developmental gain versus loss over time in hindbrain.

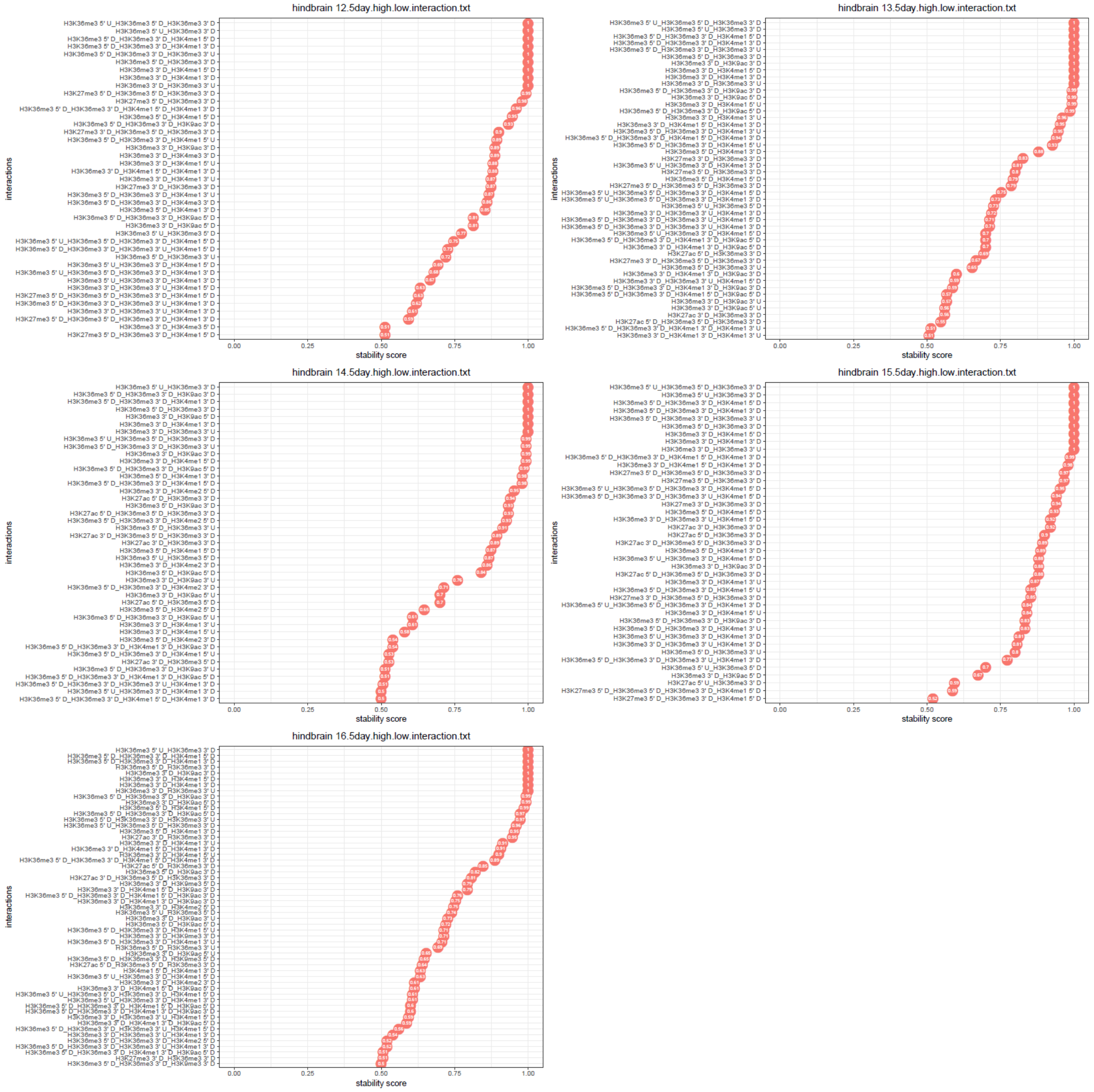

Supplemental figure 59: Contributions of different types of hPTMs to differentiate isoform selected high/low over time in hindbrain.

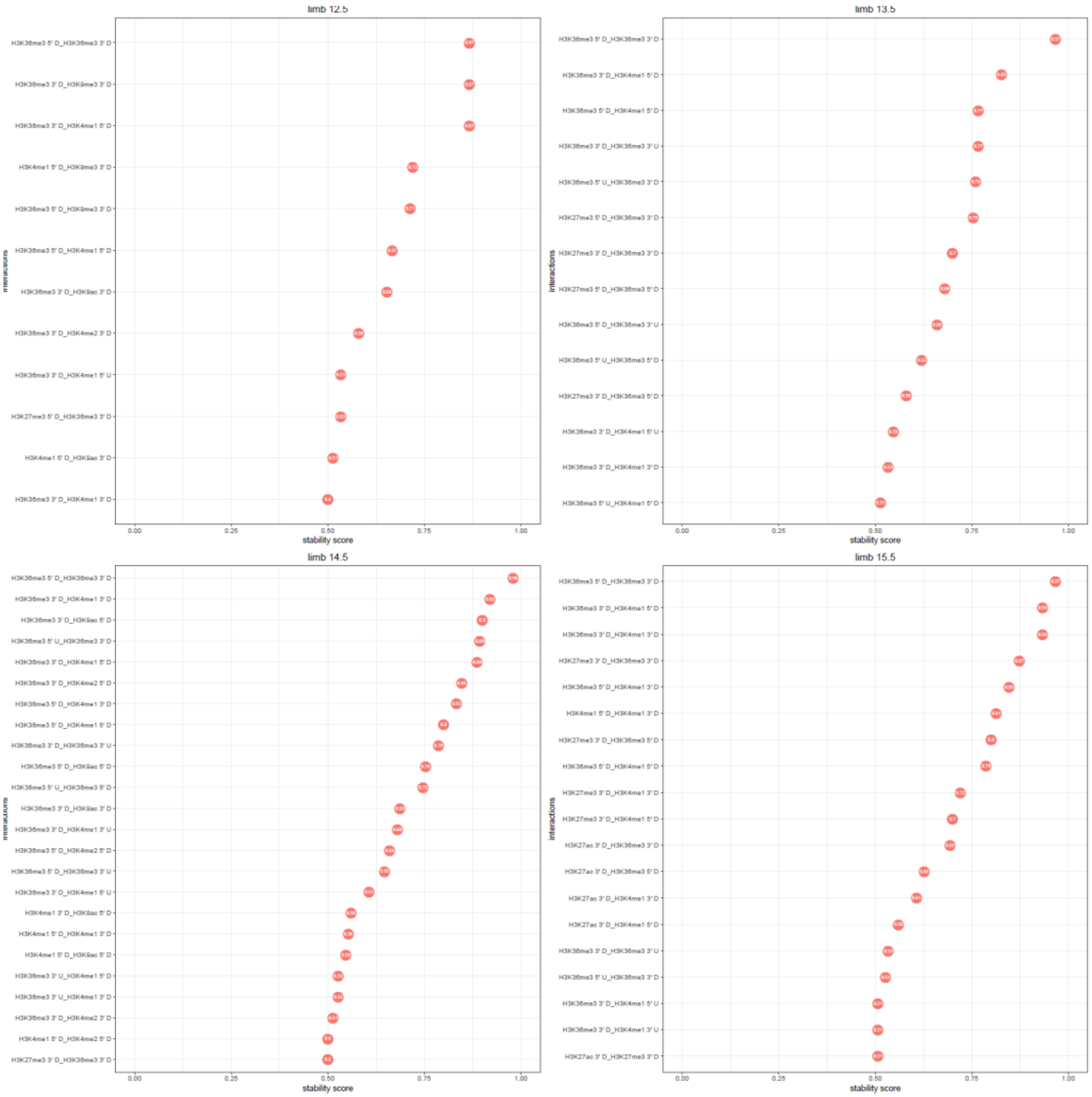

Supplemental figure 60: Contributions of different types of hPTMs to differentiate developmental gain versus loss over time in limb.

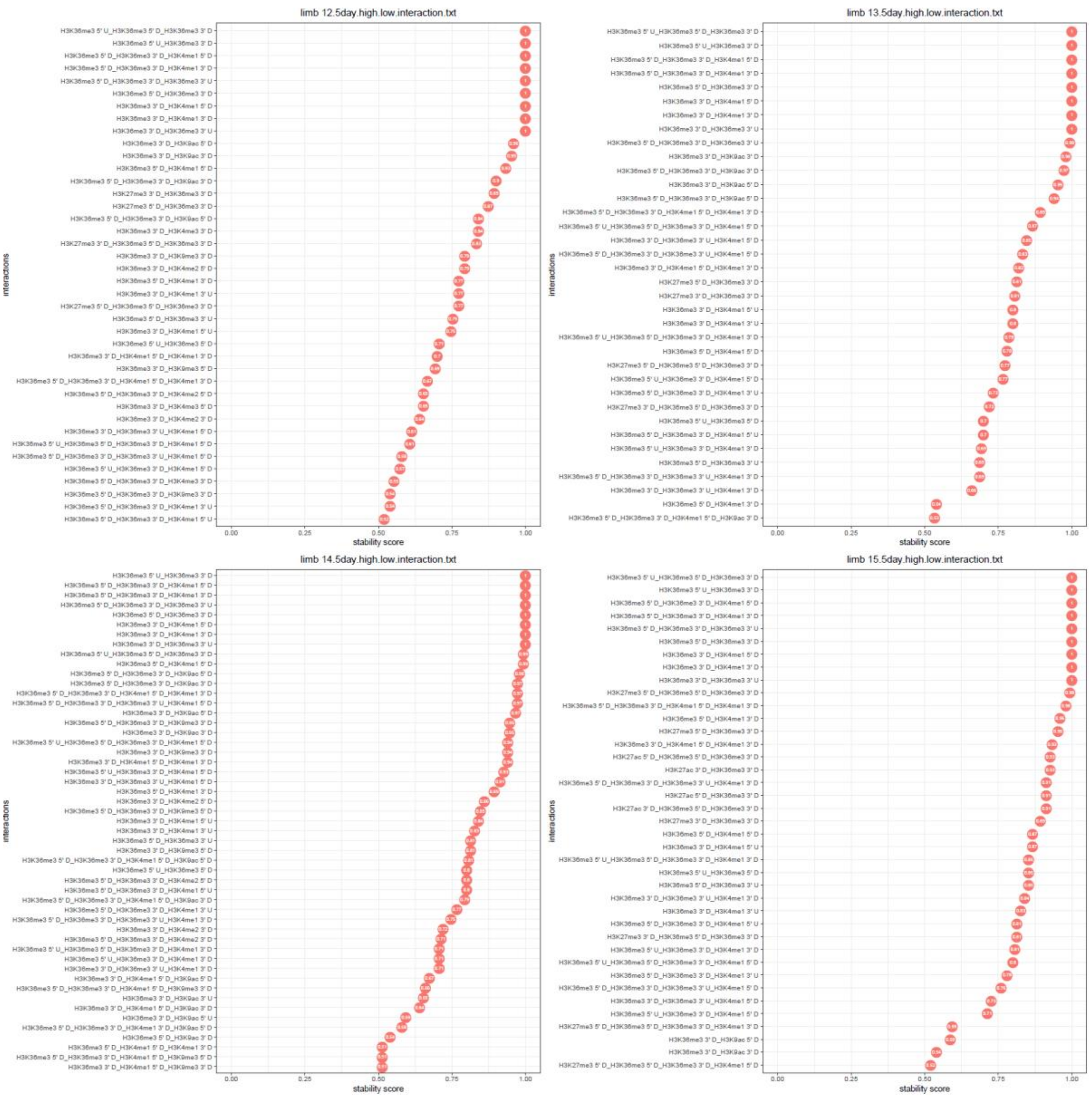

Supplemental figure 61: Contributions of different types of hPTMs to differentiate isoform selected high/low over time in limb.

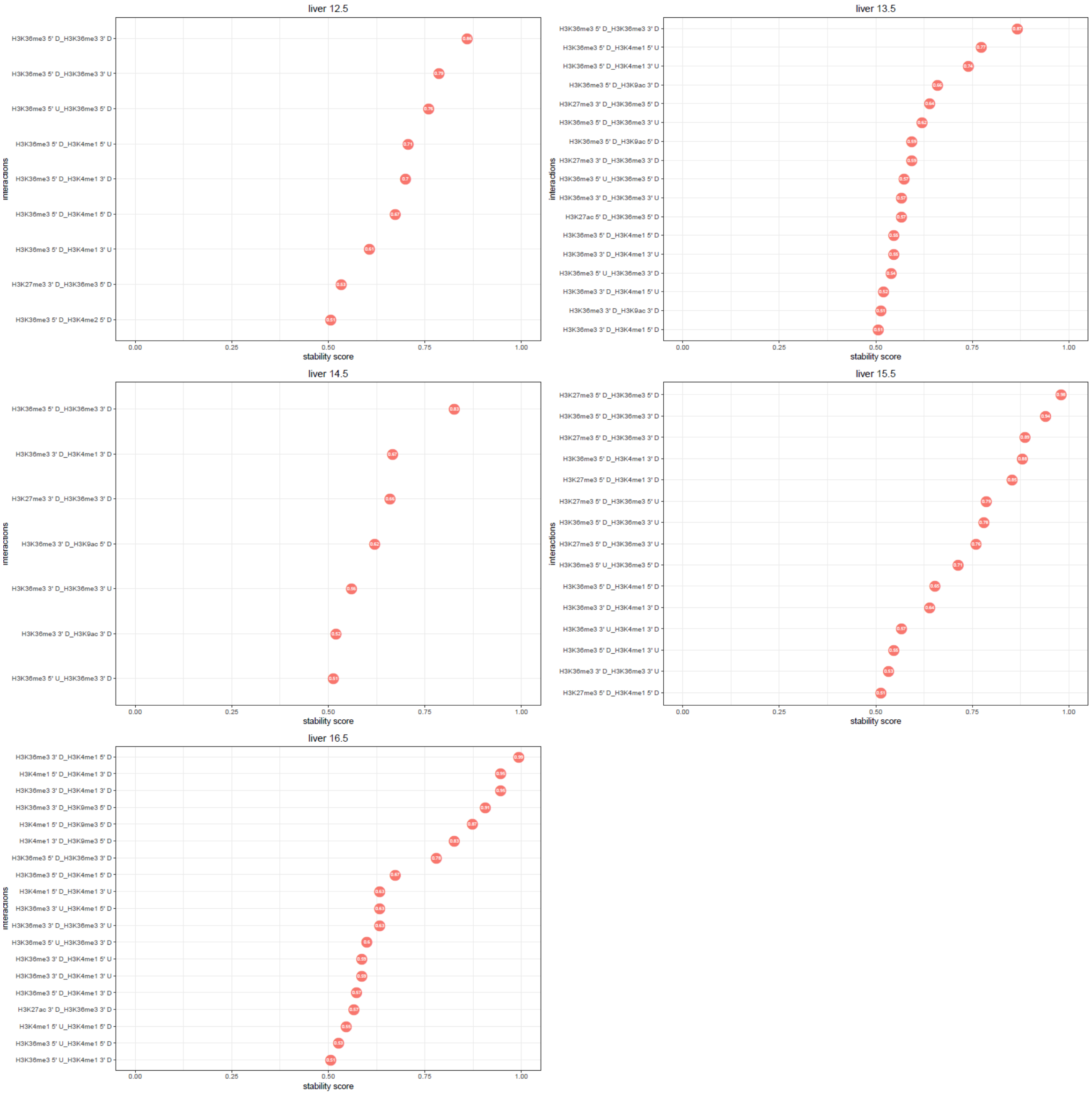

Supplemental figure 62: Contributions of different types of hPTMs to differentiate developmental gain versus loss over time in liver.

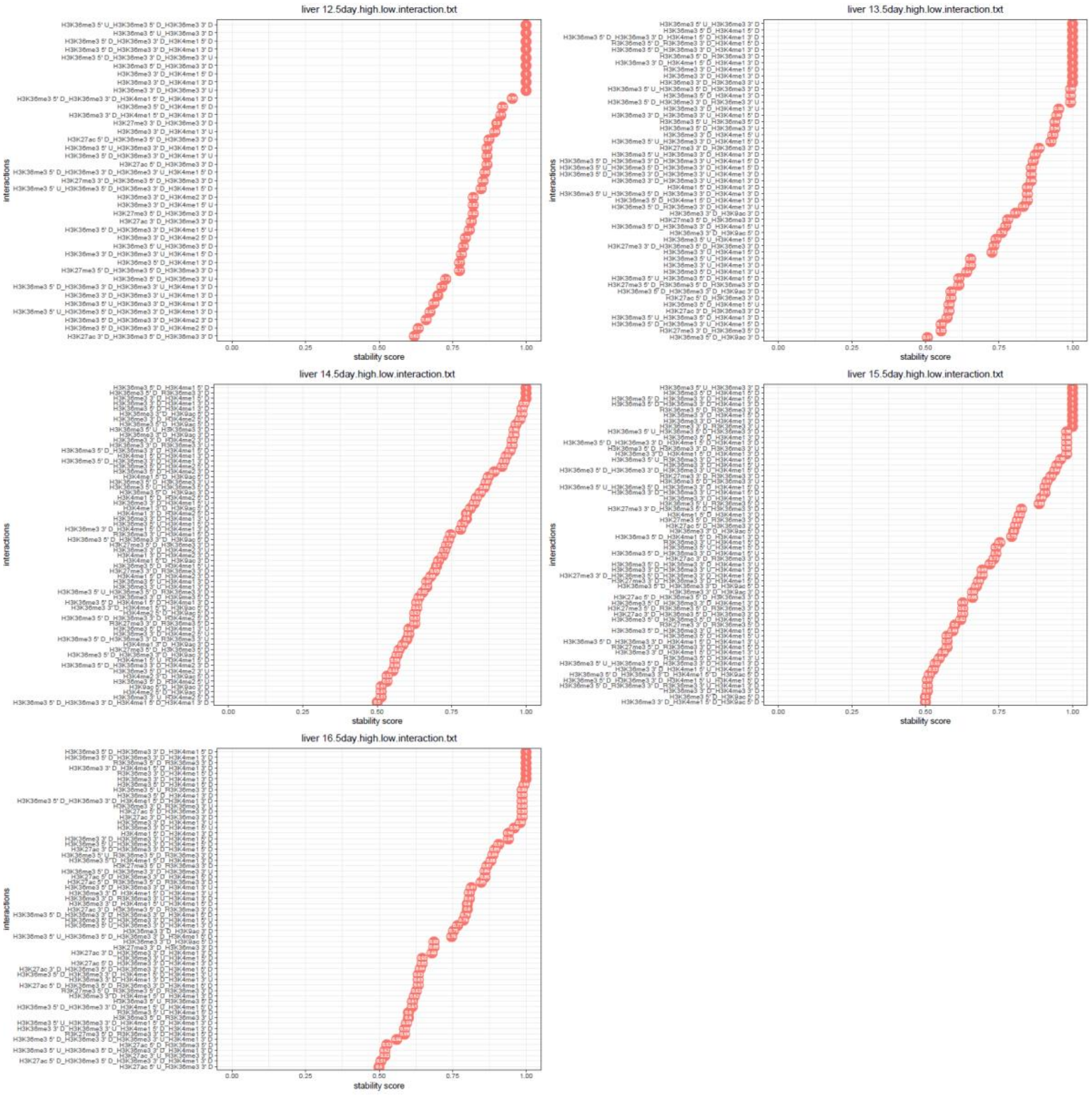

Supplemental figure 63: Contributions of different types of hPTMs to differentiate isoform selected high/low over time in liver.

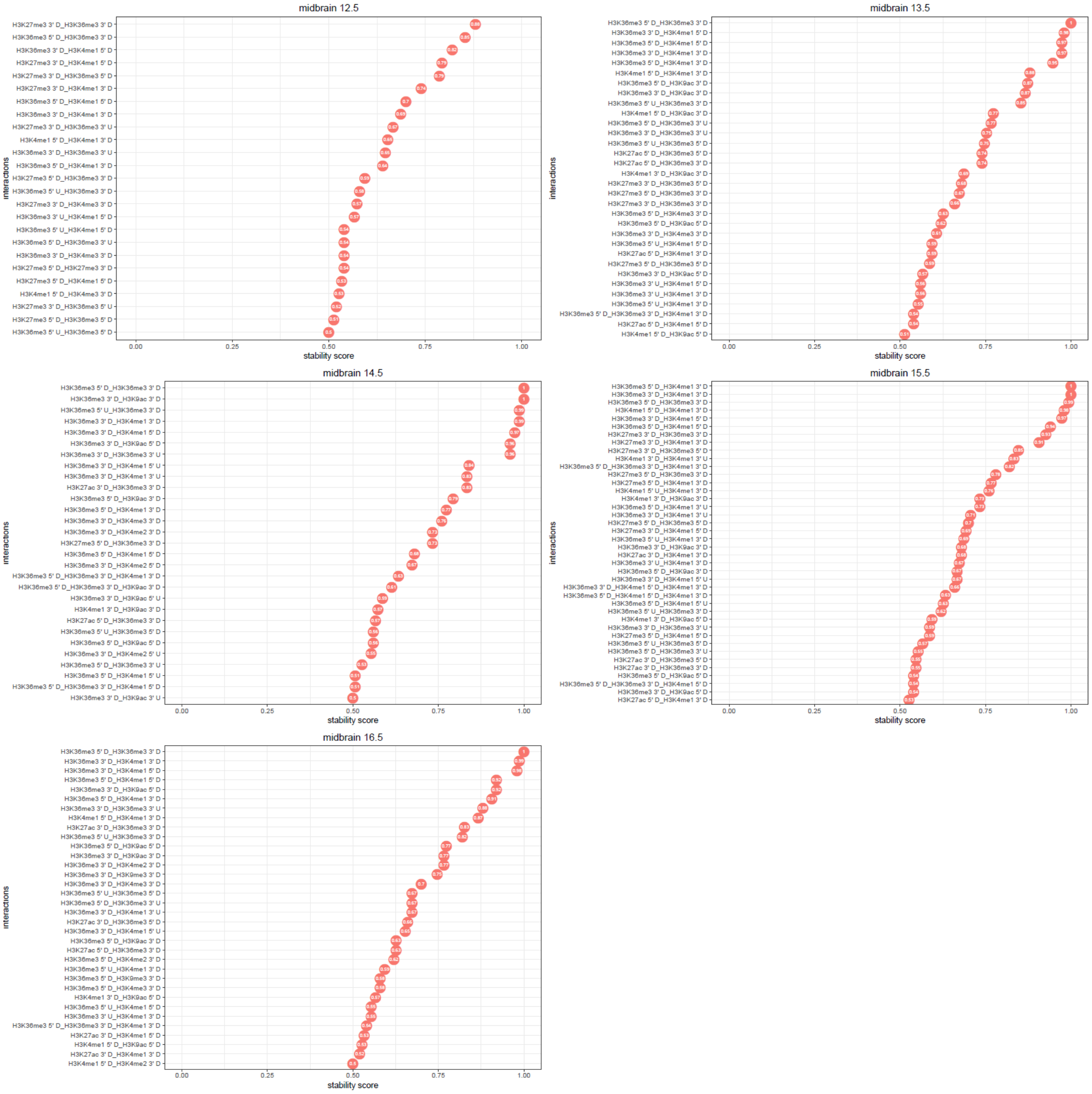

Supplemental figure 64: Contributions of different types of hPTMs to differentiate developmental gain versus loss over time in midbrain.

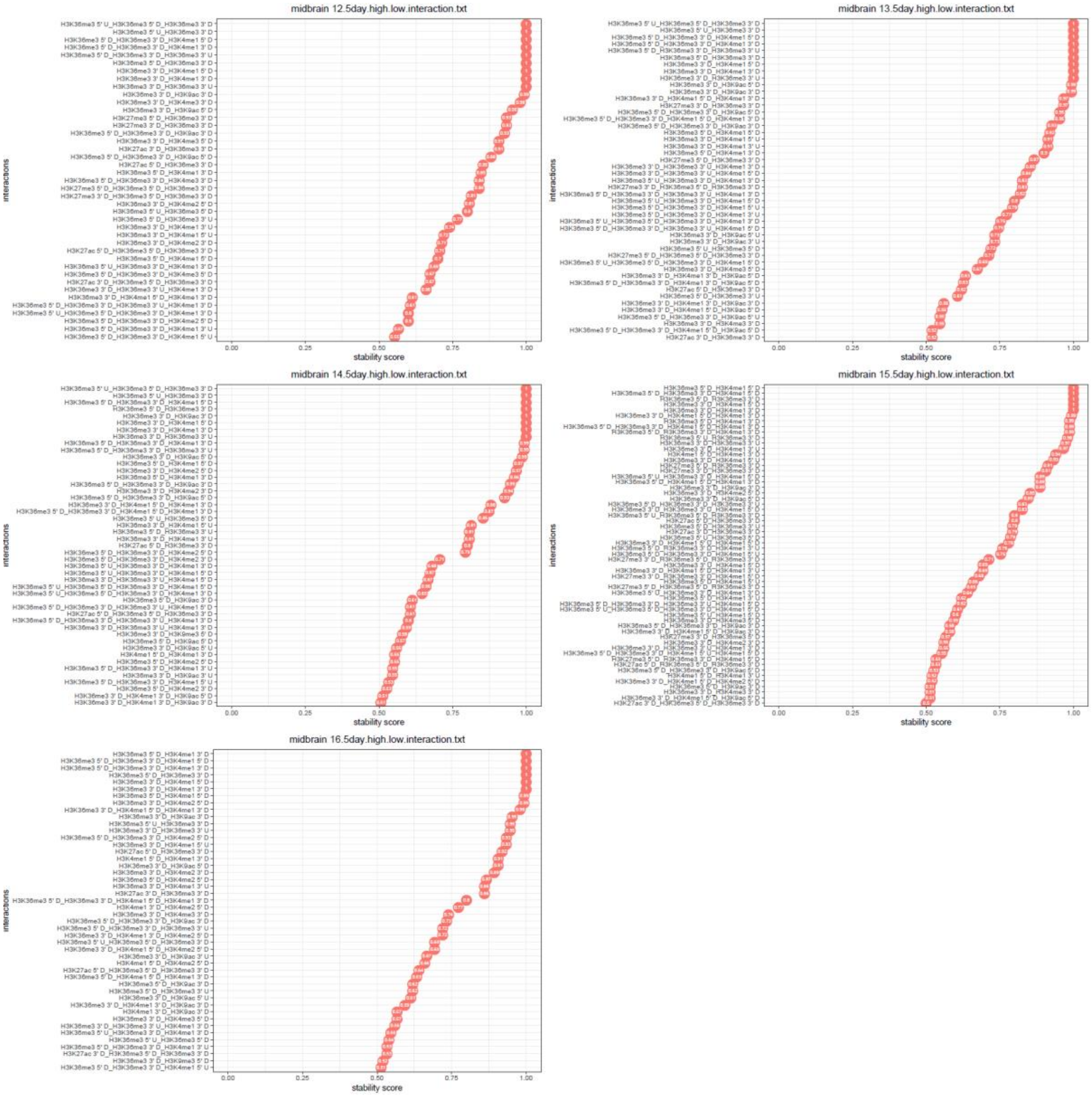

Supplemental figure 65: Contributions of different types of hPTMs to differentiate isoform selected high/low over time in midbrain.

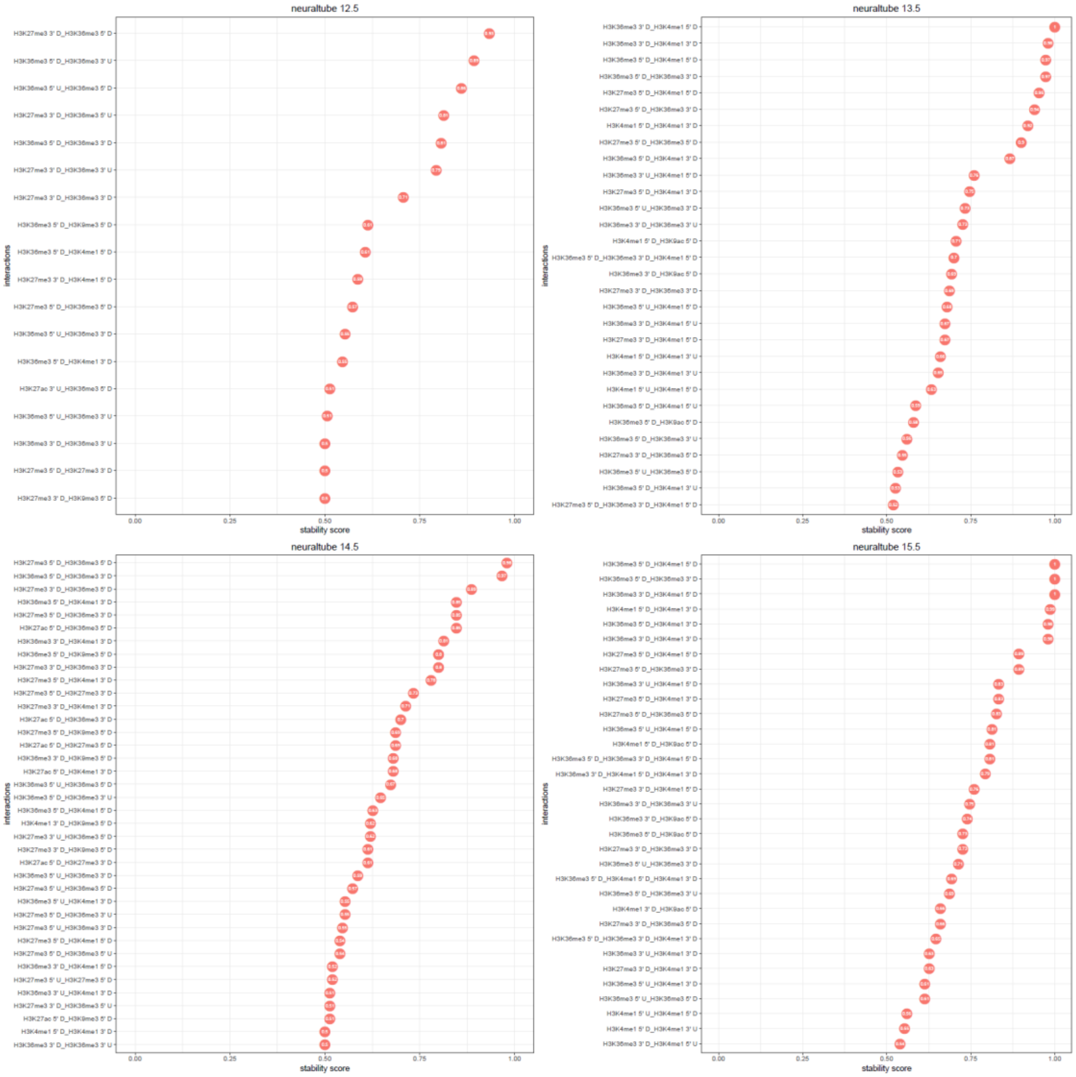

Supplemental figure 66: Contributions of different types of hPTMs to differentiate developmental gain versus loss over time in neuraltube.

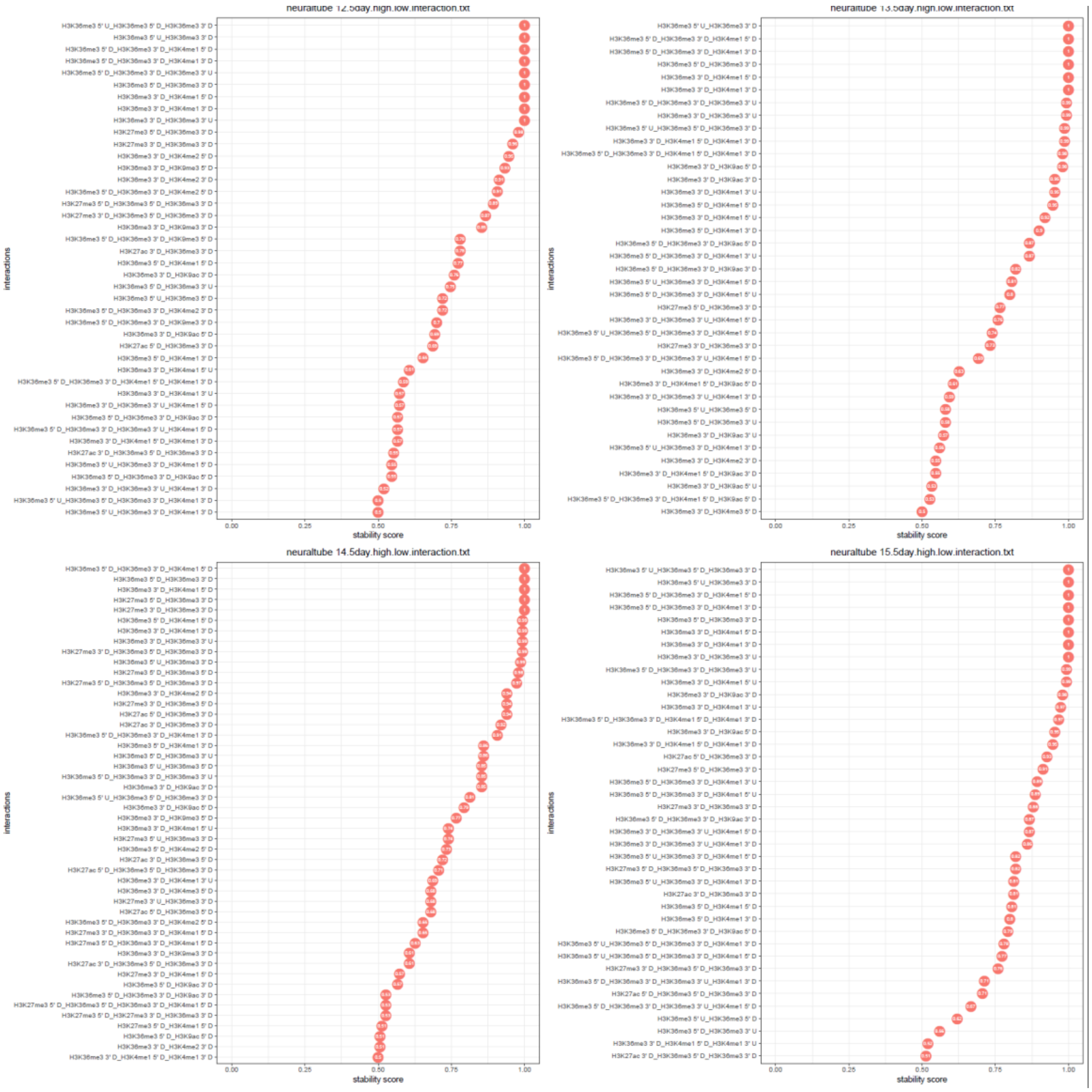

Supplemental figure 67: Contributions of different types of hPTMs to differentiate isoform selected high/low over time in neuraltube.

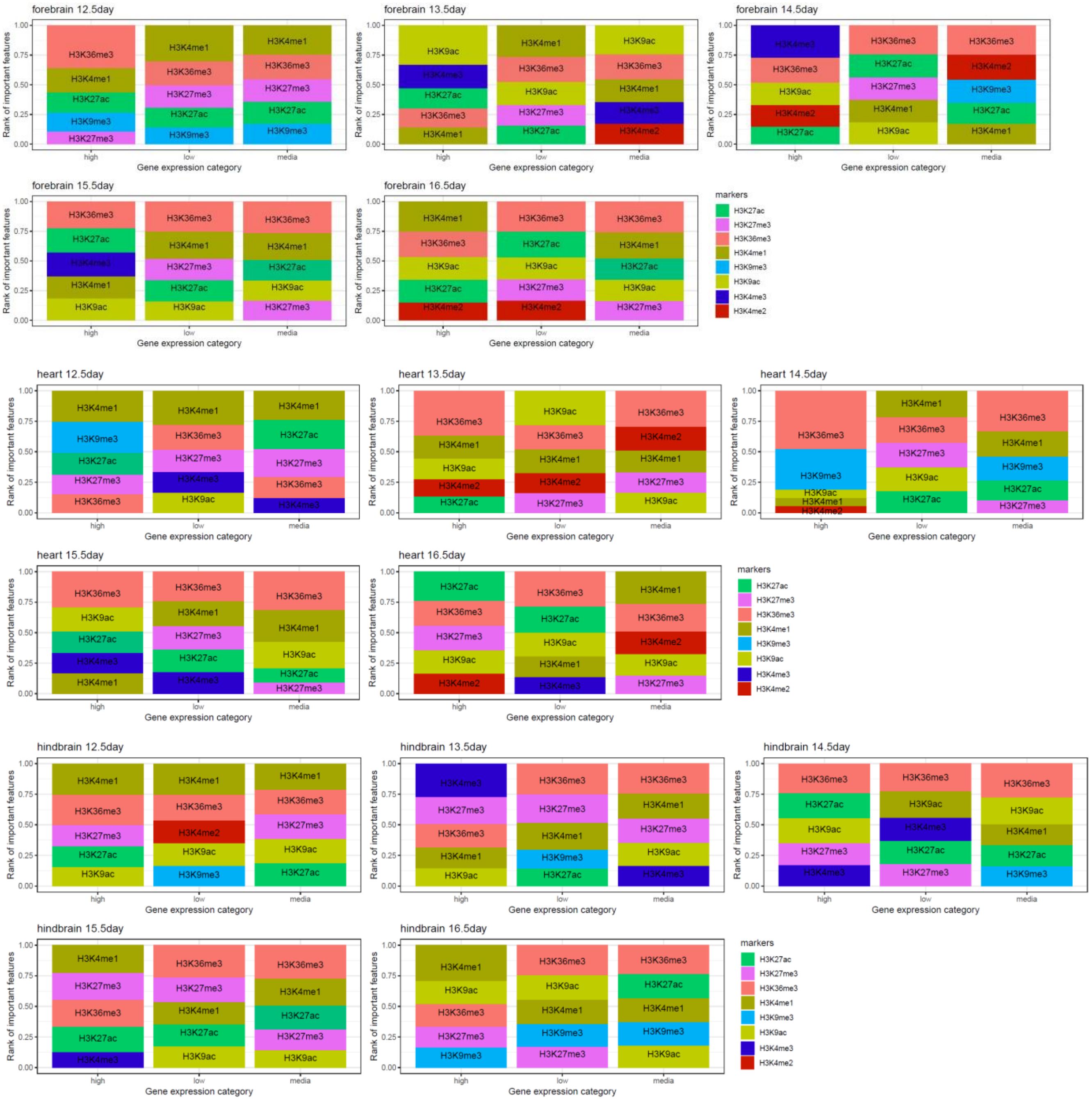

Supplemental figure 68: Contributions of top 5 markers after stratifying gene expression in forebrain, heart and hindbrain at different timepoints in developmental gain/loss exons.

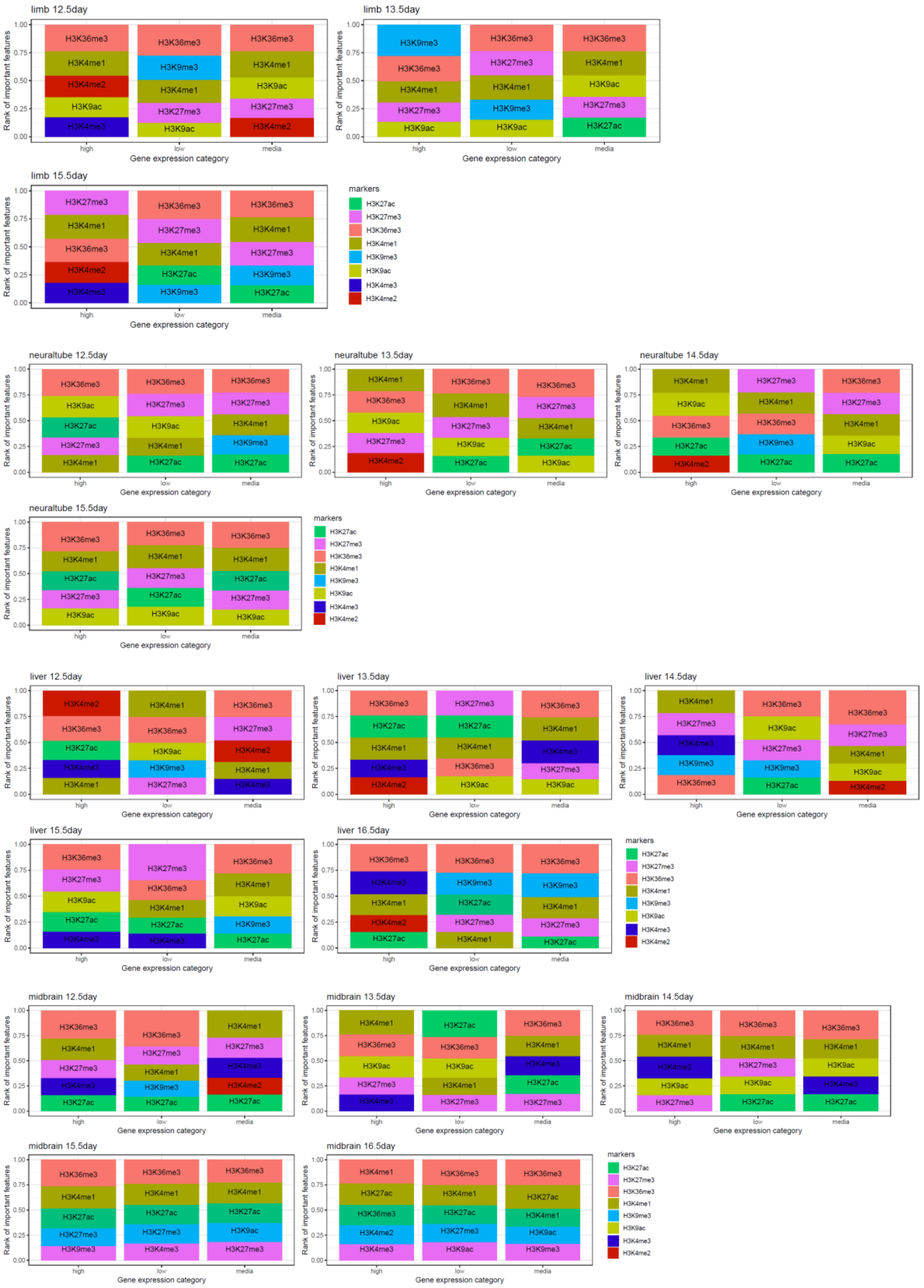

Supplemental figure 69: Contributions of top 5 markers after stratifying gene expression in limb, neuraltube, liver and midbrain at different timepoints in developmental gain/loss exons

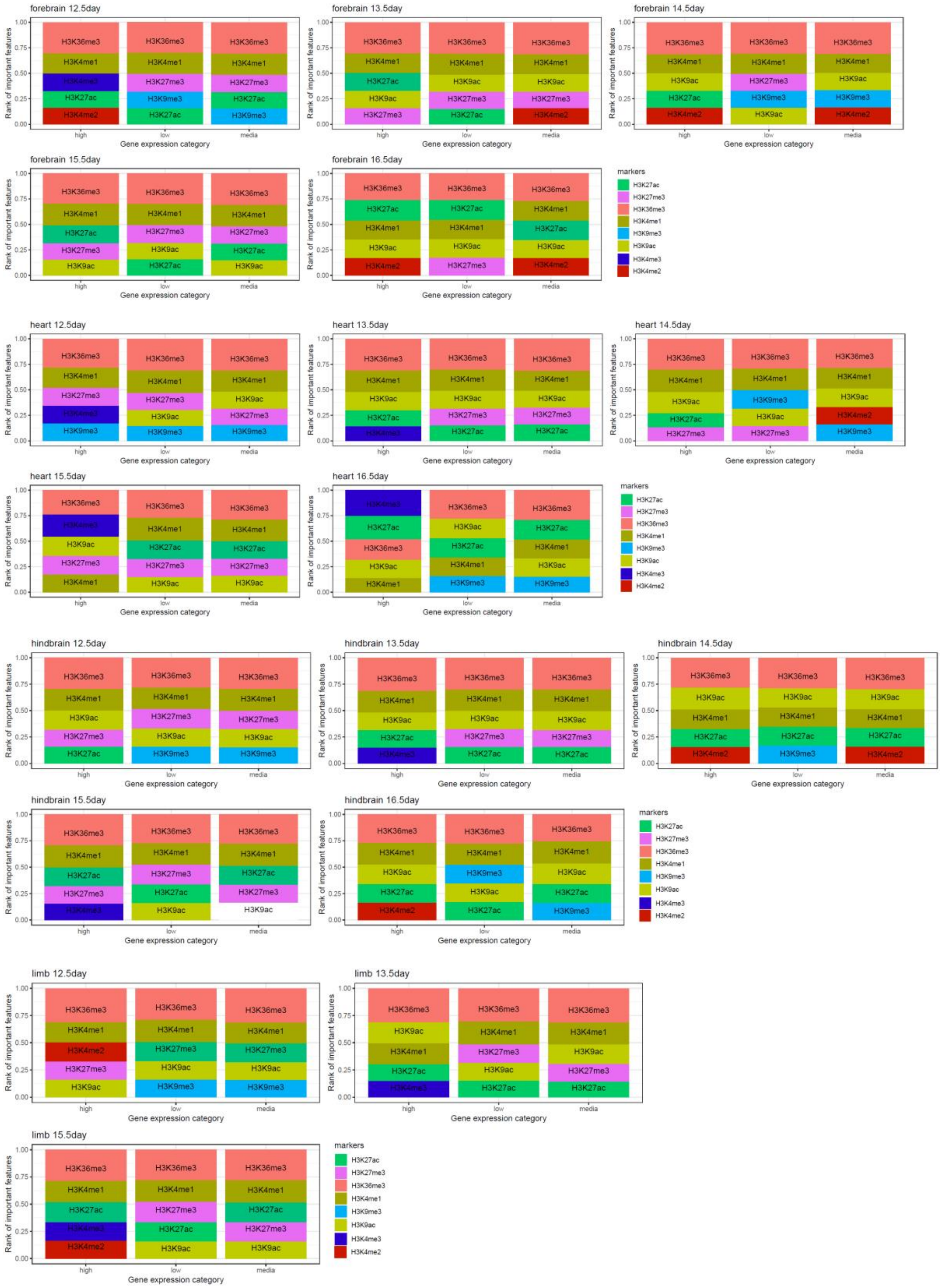

Supplemental figure 70: Contributions of top 5 markers after stratifying gene expression in forebrain, heart, hindbrain and limb at different timepoints in isoform selected high/low exons

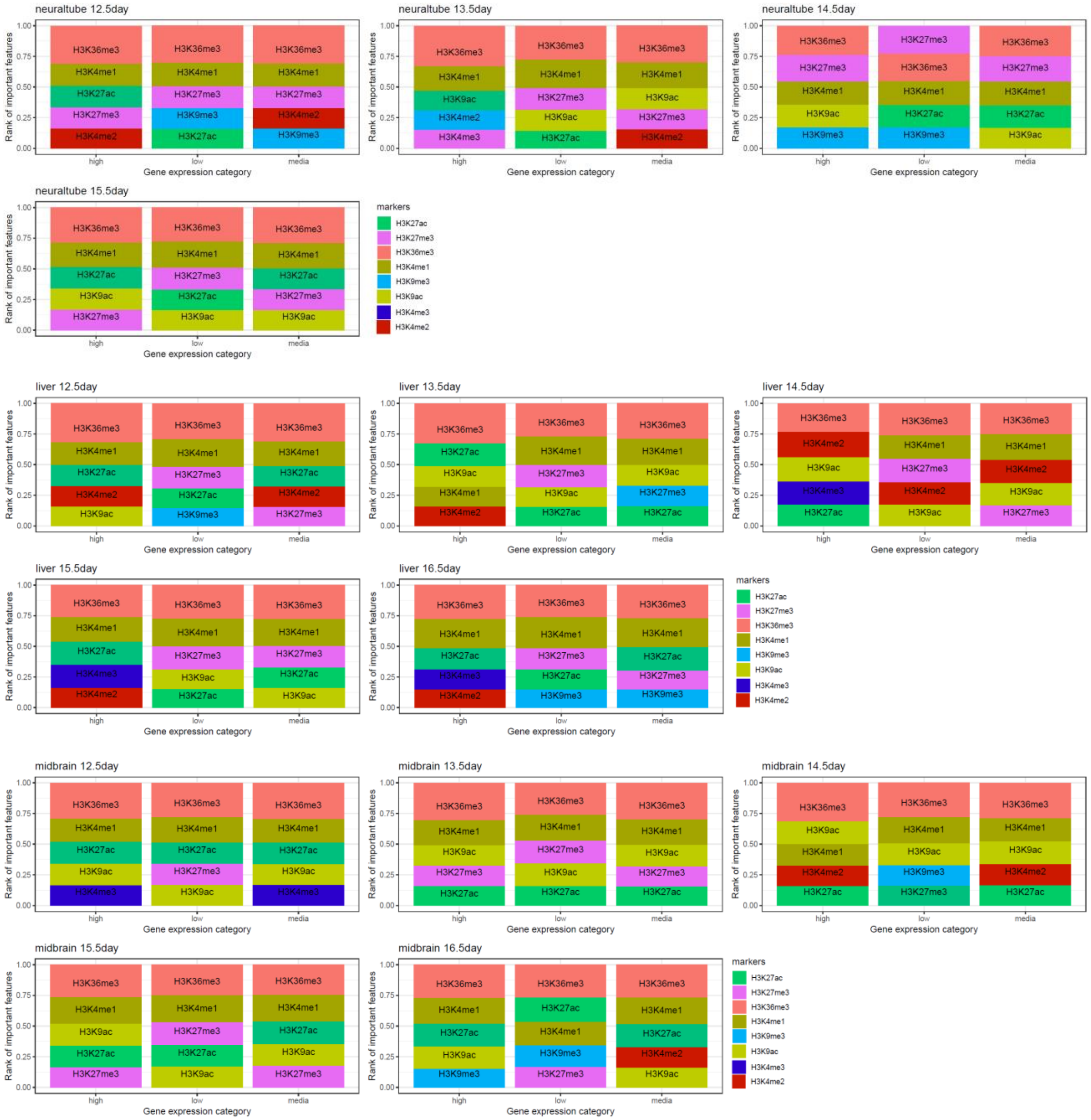

Supplemental figure 71: Contributions of top 5 markers after stratifying gene expression in neuraltube, heart, liver and midbrain at different timepoints in isoform selected high/low exons

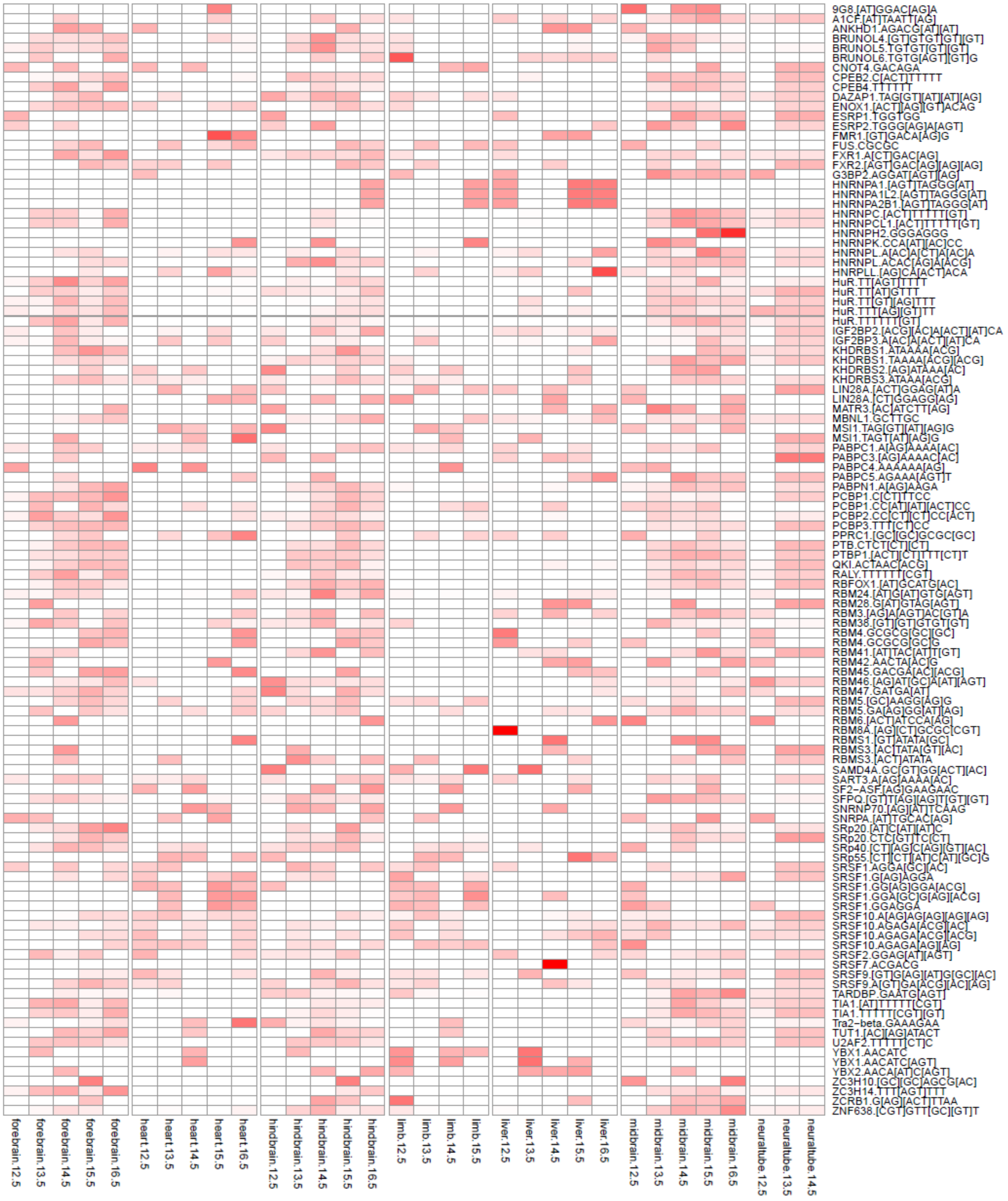

Supplemental figure 72: Enrichment of motifs in the flanking regions of developmental gain/loss exons in different tissues across developmental timepoints. Enrichment score is calculated as -logPvale generated by MAPS2 (http://rmaps.cecsresearch.org/MTool/).

A

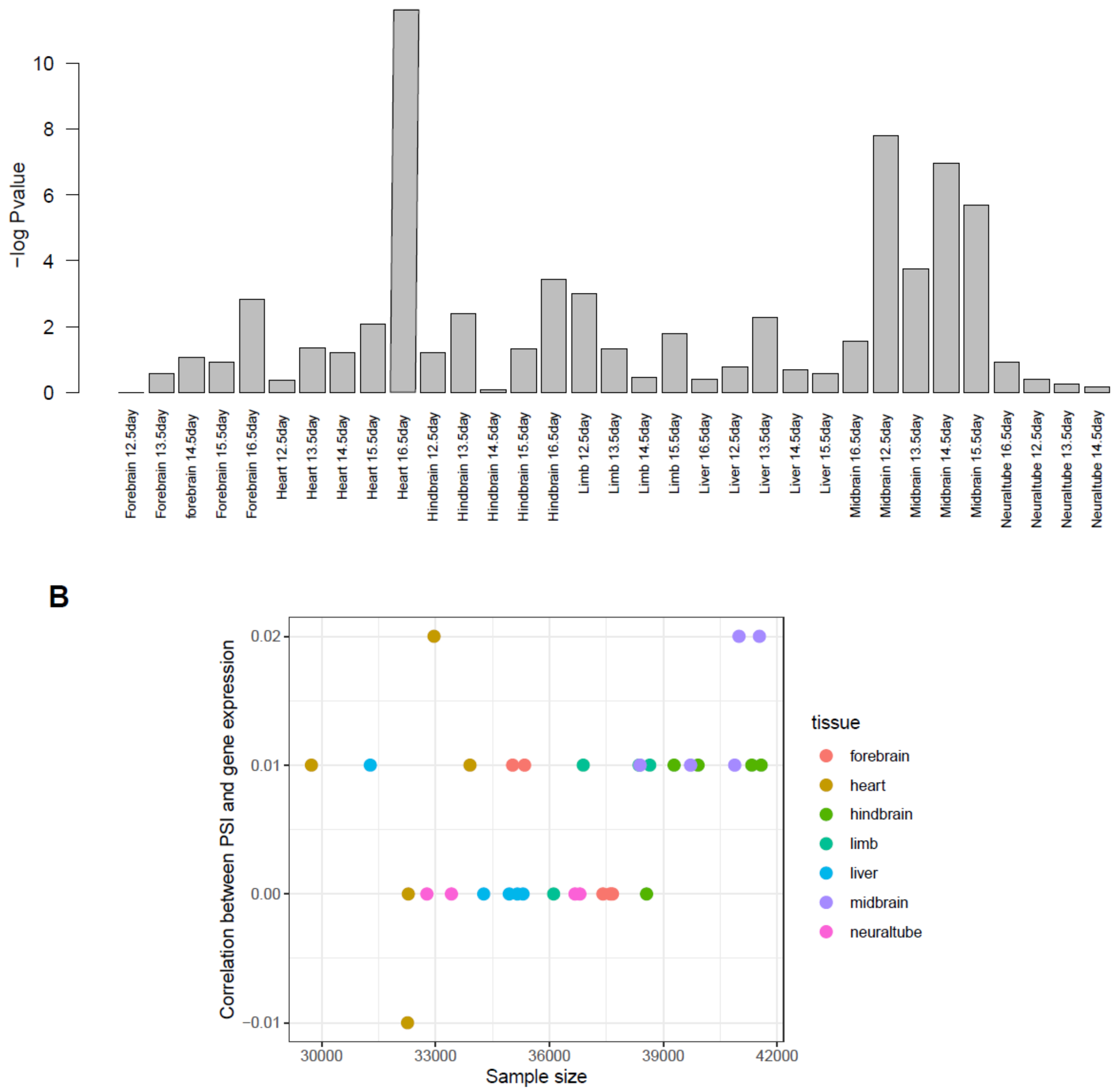

Supplemental figure 73: Correlation between PSI and gene expression across different tissues. A: Distribution of -log Pvalues across tissues. P-values were calculated based on correlation test between PSI values and gene expression. B: scatter plot shows the distribution of correlation and sample size.

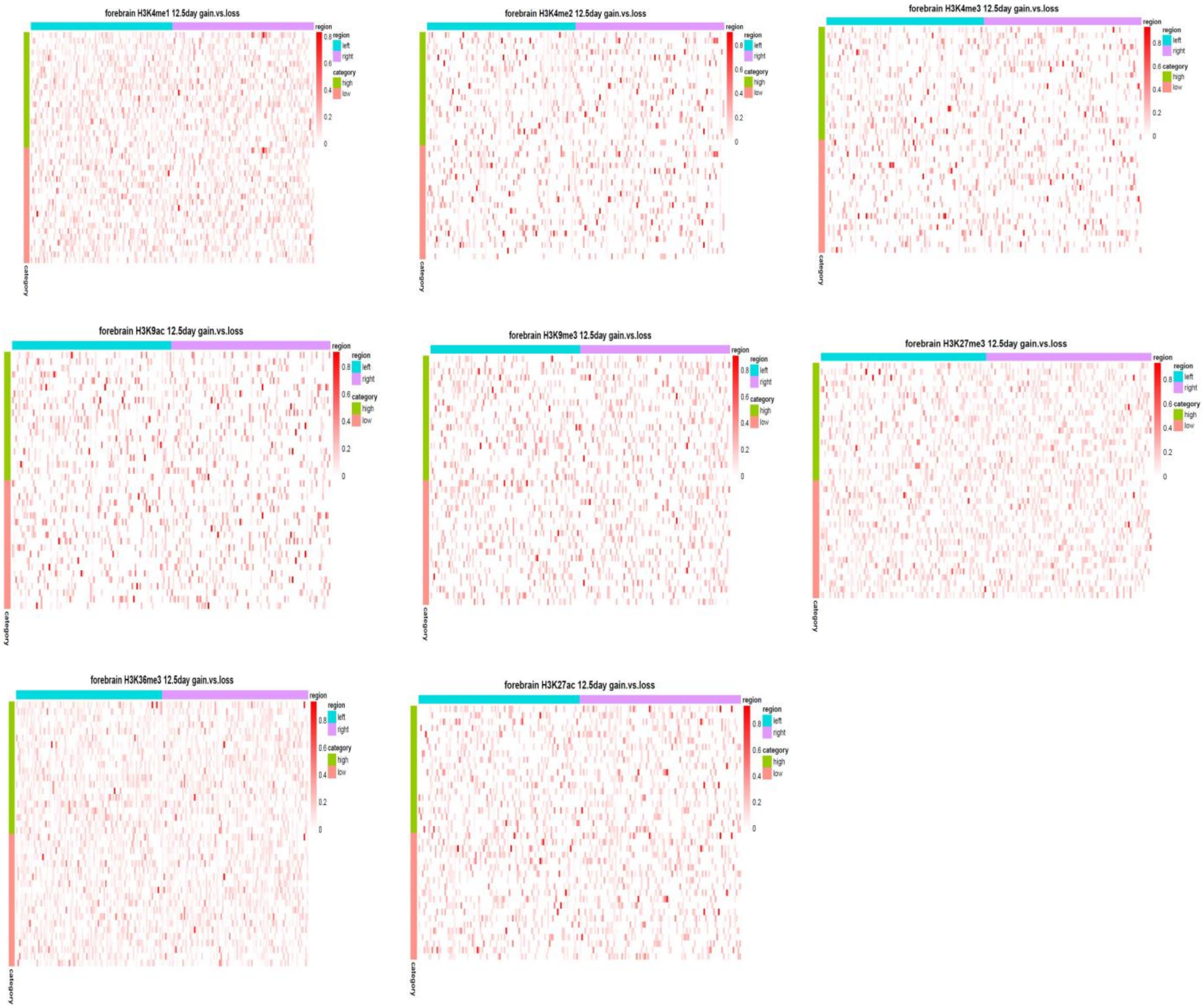

Supplemental figure 74: Heatmap of Chip-seq coverage in the flanking region of sampled splicing exons in forebrain.
